# Supplementary material for: Development of summary indices of antenatal care service quality in Haiti, Malawi and Tanzania
Source: BMJ Open. 2019 Dec 2;9(12):e032558. doi: 10.1136/bmjopen-2019-032558 (PMC7003378; doi:10.1136/bmjopen-2019-032558)
Supplement: Supplementary data [file bmjopen-2019-032558supp001.pdf]

SUPPLEMENTARY TABLES

Supplementary Table 1: Summary of Literature Using SPA Data to Create ANC Quality of Care Indices

Author	Year	Country	Type of index and number of items	Item selection	Item combination
Diamond-Smith et al	2016	Kenya, Namibia	Facility readiness: 8 items	DHS tracer indicators of ANC quality	Simple additive
Do et al	2017	Kenya, Namibia	Facility readiness: 14 items Provision of care: 7 items	Donabedian framework and the WHO SARA indicators	Factor analysis
Kruk et al	2017	Kenya, Malawi, Namibia, Rwanda, Senegal, Uganda, Tanzania	Provision of care: 8 items	Clinical guidelines from the WHO	Simple additive
Lee et al	2016	Kenya	Facility readiness and provision of care: 14 items	WHO definition of quality of care	Weighted additive
Sharma et al	2017	Kenya	Facility readiness: 15 items Provision of care: 40 items	Donabedian framework, IOM definition of quality of care, and Focused ANC Model Checklist	Simple additive
Sipsma et al	2012	Rwanda	Provision of care: 8 items	Recommended practices derived from clinical guidelines	Simple additive

Sheffel A, *et al. BMJ Open* 2019; 9:e032558. doi: 10.1136/bmjopen-2019-032558

Supplementary Table 2: List of Items Included in the Facility Readiness Indices, by Domain

#	Item Name	Core set	Expert survey	Maximum set
	EQUIPMENT AND SUPPLIES *Availability of…*			
1	Blood pressure apparatus	X	X	X
2	Examination light	X		X
3	Stethoscope	X	X	X
4	Adult weighing scale	X		X
5	Tape measure for fundal height	X		X
6	Examination bed	X	X	X
7	Latex gloves		X	X
8	Single use syringes	X	X	X
9	Soap and water OR alcohol-based hand rub		X	X
10	Disinfectant		X	X
11	Appropriate storage of sharps waste (sharps box)			X
12	Appropriate storage of infectious waste (pedal bin with lid and liner)			X
13	Safe final disposal of sharps (incineration)			X
14	Safe final disposal of infectious wastes (incineration)			X
15	Medical masks			X
16	Gowns			X
17	Eye protection			X
	DIAGNOSTICS *Availability on-site to test for…*			
18	Hemoglobin	X	X	X
19	Urine dipstick- protein	X	X	X
20	Urine dipstick- glucose	X	X	X
21	Grouping and Rhesus factor	X		X
22	Syphilis RDT / RPR	X	X	X
23	HIV testing / RDT	X	X	X
	MEDICINES AND COMMODITIES *Availability on-site of…*			
24	Iron tablets	X	X	X

Sheffel A, *et al. BMJ Open* 2019; 9:e032558. doi: 10.1136/bmjopen-2019-032558

#	Item Name	Core set	Expert survey	Maximum set
25	Folic acid tablets	X	X	X
26	Tetanus toxoid vaccine	X	X	X
27	De-worming drugs (mebendazole / albendazole)	X		X
	BASIC AMENITIES<br>*Availability of…*			
28	Power			X
29	Improved water source		X	X
30	Room with auditory and visual privacy	X	X	X
31	Sanitation facilities		X	X
32	Communication equipment			X
33	Computer with email/internet access			X
34	Emergency transportation			X
	HUMAN RESOURCES<br>*Availability of…*			
35	Guidelines for ANC available at the facility	X		X
36	Proportion of health facility staff providing ANC services trained in ANC in the last two years	X	X	X
37	Proportion of health facility staff providing ANC services who received supervision in the last six months	X		X
38	Proportion of health facility staff providing ANC services who report availability of opportunities for promotion in their current job			X

Sheffel A, *et al. BMJ Open* 2019; 9:e032558. doi: 10.1136/bmjopen-2019-032558

Supplementary Table 3: List of Items Included in the Provision of Care Indices, by Domain

#	Item Name	Corresponding Readiness Domain
	HISTORY-TAKING AND CLIENT EDUCATION/COUNSELLING<br>*During ANC visit the provider asks about/counsels the client on…*	
1	Personal history: client age	Human Resources
2	Personal history: medications client is taking	Human Resources
3	Personal history: date last menstrual period began	Human Resources
4	Personal history: any prior pregnancy	Human Resources
5	Past medical history for prior pregnancies: still birth	Human Resources
6	Past medical history for prior pregnancies: infant died in the first week of life	Human Resources
7	Past medical history for prior pregnancies: heavy bleeding during or after delivery	Human Resources
8	Past medical history for prior pregnancies: previous assisted delivery	Human Resources
9	Past medical history for prior pregnancies: previous spontaneous abortion	Human Resources
10	Past medical history for prior pregnancies: multiple pregnancies	Human Resources
11	Past medical history for prior pregnancies: prolonged labor	Human Resources
12	Past medical history for prior pregnancies: pregnancy-induced hypertension	Human Resources
13	Past medical history for prior pregnancies: pregnancy related convulsions	Human Resources
14	Past medical history for prior pregnancies: high fever or infection during prior pregnancy	Human Resources
15	History of complaints in current pregnancy: vaginal bleeding	Human Resources
16	History of complaints in current pregnancy: fever	Human Resources
17	History of complaints in current pregnancy: headache or blurred vision	Human Resources
18	History of complaints in current pregnancy: swollen face or hands or extremities	Human Resources
19	History of complaints in current pregnancy: tiredness or breathlessness	Human Resources
20	History of complaints in current pregnancy: fetal movement (loss of, excessive, normal)	Human Resources
21	History of complaints in current pregnancy: cough or difficulty breathing for three weeks or longer	Human Resources
22	Client education/counselling: Process of pregnancy and its complications	Human Resources
23	Client education/counselling: Diet and nutrition	Human Resources
24	Client education/counselling: Danger signs in pregnancy	Human Resources

Sheffel A, *et al. BMJ Open* 2019; 9:e032558. doi: 10.1136/bmjopen-2019-032558

#	Item Name	Corresponding Readiness Domain
25	Client education/counselling: Voluntary counselling and testing for HIV	Human Resources
26	Client education/counselling: Breastfeeding	Human Resources
27	Client education/counselling: Plans of delivery (emergency preparedness, place of delivery, transportation, financial arrangements)	Human Resources
	EXAMINATION *During ANC visit the provider examines the client for…*	
28	Oedema	Human Resources
29	Blood pressure	Human Resources, Equipment
30	Weight	Human Resources, Equipment
31	Palpating the client's abdomen for fundal height	Human Resources, Equipment
	DIAGNOSTICS *During ANC visit the provider performs or refers the client to test for…*	
32	Hemoglobin	Human Resources, Diagnostics
33	Grouping and rhesus factor	Human Resources, Diagnostics
34	Syphilis / RPR	Human Resources, Diagnostics
35	HIV	Human Resources, Diagnostics
36	Urine – protein, glucose	Human Resources, Diagnostics
	PREVENTATIVE TREATMENT *During ANC visit the provider administers or prescribes…*	
37	Iron and/or folic acid	Human Resources, Medicines
38	Tetanus toxoid	Human Resources, Medicines
	CLIENT EXPERIENCE *During ANC visit…*	
39	Client is able to discuss problems or concerns with provider	Human Resources
40	Client satisfied with the amount of explanation received about the problem or treatment	Human Resources

Sheffel A, *et al. BMJ Open* 2019; 9:e032558. doi: 10.1136/bmjopen-2019-032558

#	Item Name	Corresponding Readiness Domain
41	Client satisfied with how the staff treated them	Human Resources
42	Privacy from having others see the consultation	Human Resources, Basic Amenities
43	Privacy from having others hear the consultation	Human Resources, Basic Amenities
44	Client satisfied with the wait time	Human Resources
45	Client satisfied with the number of days services are available at the facility	Human Resources
46	Client satisfied with the hours of service at the facility	Human Resources
47	Client satisfied with the cost for services or treatments	Human Resources
48	Client satisfied with the availability of medicines at the facility	Human Resources, Medicines
49	Client satisfied with the cleanliness of the facility	Human Resources

Sheffel A, *et al. BMJ Open* 2019; 9:e032558. doi: 10.1136/bmjopen-2019-032558

Supplementary Table 4: Expert Survey Results (mean, sd, min, max, n)

Item Name	Mean	Standard Deviation	Min.	Max.	N
EQUIPMENT AND SUPPLIES *Availability of…*					
Blood pressure apparatus	3.933	0.258	3	4	15
Examination light	2.600	0.828	1	4	15
Fetal stethoscope	3.733	0.594	2	4	15
Stethoscope	3.467	0.834	2	4	15
Adult weighing scale	3.000	0.845	2	4	15
Thermometer	3.533	0.834	1	4	15
Tape measure for fundal height	3.333	0.724	2	4	15
Height board	2.692	0.947	1	4	13
Speculum	3.429	0.646	2	4	14
Examination bed	3.429	0.938	1	4	14
Latex gloves	3.600	0.632	2	4	15
Single use syringes	3.643	0.745	2	4	14
Soap and water OR alcohol-based hand rub	3.733	0.704	2	4	15
Disinfectant	3.533	0.640	2	4	15
Appropriate storage of sharps waste (sharps box)	3.400	0.910	1	4	15
Appropriate storage of infectious waste (pedal bin with lid and liner)	3.333	0.976	1	4	15
Safe final disposal of sharps (incineration)	3.200	0.941	1	4	15
Safe final disposal of infectious wastes (incineration)	3.133	0.990	1	4	15
Medical masks	2.200	0.775	1	4	15
Gowns	2.000	0.845	1	4	15
Eye protection	2.067	0.884	1	4	15
Gum boots	1.667	0.724	1	3	15
DIAGNOSTICS *Availability on-site to test for…*					
Hemoglobin	3.800	0.414	3	4	15
Urine dipstick- protein	3.733	0.458	3	4	15

Sheffel A, *et al. BMJ Open* 2019; 9:e032558. doi: 10.1136/bmjopen-2019-032558

Item Name	Mean	Standard Deviation	Min.	Max.	N
Urine dipstick- glucose	3.467	0.743	2	4	15
Grouping and Rhesus factor	3.357	0.745	2	4	14
Syphilis RDT / RPR	3.667	0.488	3	4	15
HIV testing / RDT	3.667	0.488	3	4	15
MEDICINES AND COMMODITIES *Availability on-site of…*					
Iron tablets	3.800	0.414	3	4	15
Folic acid tablets	3.667	0.617	2	4	15
Tetanus toxoid vaccine	3.800	0.414	3	4	15
Intermittent Preventative Treatment (IPT) drug	3.667	0.488	3	4	15
Insecticide treated nets (ITNs) or vouchers	3.267	0.799	2	4	15
De-worming drugs (mebendazole / albendazole)	3.000	0.784	2	4	14
BASIC AMENITIES *Availability of…*					
Power	3.400	0.737	2	4	15
Improved water source	3.667	0.617	2	4	15
Room with auditory and visual privacy	3.533	0.640	2	4	15
Sanitation facilities	3.600	0.507	3	4	15
Communication equipment	3.267	0.884	1	4	15
Computer with email/internet access	2.267	1.033	1	4	15
Emergency transportation	3.400	0.986	1	4	15
HUMAN RESOURCES *Availability of…*					
Guidelines for ANC available at the facility	3.267	0.884	1	4	15
Proportion of health facility staff providing ANC services trained in ANC in the last two years	3.400	0.737	2	4	15
Proportion of health facility staff providing ANC services who received supervision in the last six months	3.267	0.799	2	4	15

Sheffel A, *et al. BMJ Open* 2019; 9:e032558. doi: 10.1136/bmjopen-2019-032558

Item Name	Mean	Standard Deviation	Min.	Max.	N
Proportion of health facility staff providing ANC services who report availability of opportunities for promotion in their current job	2.500	0.760	2	4	14
HISTORY-TAKING *During ANC visit the provider asks about…*					
Personal history: client age	3.533	0.640	2	4	15
Personal history: medications client is taking	3.733	0.458	3	4	15
Personal history: date last menstrual period began	3.933	0.258	3	4	15
Personal history: any prior pregnancy	3.867	0.352	3	4	15
Family history	3.200	0.862	2	4	15
Social history	3.067	0.704	2	4	15
Past medical history for prior pregnancies: still birth	3.867	0.352	3	4	15
Past medical history for prior pregnancies: infant died in the first week of life	3.600	0.632	2	4	15
Past medical history for prior pregnancies: heavy bleeding during or after delivery	3.733	0.458	3	4	15
Past medical history for prior pregnancies: previous assisted delivery	3.733	0.458	3	4	15
Past medical history for prior pregnancies: previous spontaneous abortion	3.467	0.834	2	4	15
Past medical history for prior pregnancies: multiple pregnancies	3.600	0.737	2	4	15
Past medical history for prior pregnancies: prolonged labor	3.600	0.632	2	4	15
Past medical history for prior pregnancies: pregnancy-induced hypertension	4.000	0.000	4	4	15
Past medical history for prior pregnancies: pregnancy related convulsions	4.000	0.000	4	4	15

Sheffel A, *et al. BMJ Open* 2019; 9:e032558. doi: 10.1136/bmjopen-2019-032558

Item Name	Mean	Standard Deviation	Min.	Max.	N
Past medical history for prior pregnancies: high fever or infection during prior pregnancy	3.533	0.640	2	4	15
History of complaints in current pregnancy: vaginal bleeding	4.000	0.000	4	4	15
History of complaints in current pregnancy: fever	3.867	0.352	3	4	15
History of complaints in current pregnancy: headache or blurred vision	4.000	0.000	4	4	15
History of complaints in current pregnancy: swollen face or hands or extremities	4.000	0.000	4	4	15
History of complaints in current pregnancy: tiredness or breathlessness	3.667	0.816	1	4	15
History of complaints in current pregnancy: fetal movement (loss of, excessive, normal)	3.933	0.258	3	4	15
History of complaints in current pregnancy: cough or difficulty breathing for three weeks or longer	3.733	0.458	3	4	15
History of complaints in current pregnancy: amniotic leakage	3.786	0.579	2	4	14
EXAMINATION<br>*During ANC visit the provider examines the client for…*					
Head to toe (whole body)	2.600	1.121	1	4	15
Pallor	3.400	0.632	2	4	15
Oedema	3.733	0.458	3	4	15
Breast	2.533	0.990	1	4	15
Lungs and heart	2.600	1.056	1	4	15
OBSERVATION AND CLINICAL INVESTIGATION<br>*During ANC visit the provider assesses the client's…*					
Blood pressure	4.000	0.000	4	4	15
Weight	3.133	0.834	2	4	15
Temperature	3.333	0.900	1	4	15
Pulse	3.357	0.929	1	4	14

Sheffel A, *et al. BMJ Open* 2019; 9:e032558. doi: 10.1136/bmjopen-2019-032558

Item Name	Mean	Standard Deviation	Min.	Max.	N
OBSTETRIC COMPLICATIONS *During ANC visit the provider assesses the client for potential obstetric complications by…*					
Palpating the client's abdomen for fundal height	3.667	0.617	2	4	15
Palpating the client's abdomen for fetal presentation	3.600	0.632	2	4	15
Listening to the client's abdomen for fetal heartbeat	3.800	0.561	2	4	15
Conducting an ultrasound/ referring client for ultrasound/ look at recent ultrasound report	2.600	0.986	1	4	15
PELVIC EXAMINATION *During ANC visit the provider conducts a pelvic exam with…*					
Soft tissue assessment/ Vaginal examination	2.154	1.214	1	4	13
Bony pelvic assessment	2.571	1.342	1	4	14
LABORATORY INVESTIGATIONS *During ANC visit the provider performs or refers the client to test for…*					
Hemoglobin	3.733	0.458	3	4	15
Grouping and rhesus factor	3.357	0.633	2	4	14
Syphilis / RPR	3.667	0.488	3	4	15
HIV	3.867	0.352	3	4	15
Urine – protein	3.667	0.617	2	4	15
Urine – glucose	3.400	0.828	2	4	15
Urine – acetone	2.500	1.087	1	4	12
DRUG ADMINISTRATION AND IMMUNIZATION *During ANC visit the provider administers or prescribes…*					
Iron and/or folic acid	3.800	0.414	3	4	15
Antimalarials	3.429	0.756	2	4	14
Tetanus toxoid	3.600	0.737	2	4	15
CLIENT EDUCATION AND COUNSELLING *During ANC visit the provider counsels the client on…*					
Process of pregnancy and its complications	3.733	0.594	2	4	15
Diet and nutrition	3.600	0.632	2	4	15

Sheffel A, *et al. BMJ Open* 2019; 9:e032558. doi: 10.1136/bmjopen-2019-032558

Item Name	Mean	Standard Deviation	Min.	Max.	N
Rest and exercise in pregnancy	2.933	0.704	2	4	15
Personal hygiene	2.867	0.990	1	4	15
Danger signs in pregnancy	3.933	0.258	3	4	15
Use of drugs in pregnancy	3.400	0.828	2	4	15
Effects of STI/HIV/AIDS	3.467	0.834	2	4	15
Voluntary counselling and testing for HIV	3.667	0.617	2	4	15
Care of breasts	2.867	0.743	2	4	15
Breastfeeding	3.867	0.352	3	4	15
Symptoms/signs of labor	3.867	0.516	2	4	15
Plans of delivery (emergency preparedness, place of delivery, transportation, financial arrangements)	3.733	0.458	3	4	15
Plans for postpartum care	3.400	0.632	2	4	15
Family planning	3.533	0.640	2	4	15
Harmful habits (e.g. smoking, drug abuse, alcoholism)	3.533	0.640	2	4	15
Schedule of return visit	3.867	0.352	3	4	15
CLIENT EXPERIENCE *During ANC visit…*					
Client is able to discuss problems or concerns with provider	3.867	0.352	3	4	15
Client satisfied with the amount of explanation received about the problem or treatment	3.600	0.632	2	4	15
Client satisfied with how the staff treated them	3.667	0.617	2	4	15
Privacy from having others see the consultation	3.533	0.640	2	4	15
Privacy from having others hear the consultation	3.533	0.640	2	4	15
Client satisfied with the wait time	3.133	0.834	2	4	15
Client satisfied with the number of days services are available at the facility	3.000	0.756	2	4	15

Sheffel A, *et al. BMJ Open* 2019; 9:e032558. doi: 10.1136/bmjopen-2019-032558

Item Name	Mean	Standard Deviation	Min.	Max.	N
Client satisfied with the hours of service at the facility	3.067	0.704	2	4	15
Client satisfied with the cost for services or treatments	3.333	0.617	2	4	15
Client satisfied with the availability of medicines at the facility	3.467	0.516	3	4	15
Client satisfied with the cleanliness of the facility	3.533	0.516	3	4	15

Sheffel A, *et al. BMJ Open* 2019; 9:e032558. doi: 10.1136/bmjopen-2019-032558
