# Supplementary material for: Development of summary indices of antenatal care service quality in Haiti, Malawi and Tanzania
Source: BMJ Open. 2019 Dec 2;9(12):e032558. doi: 10.1136/bmjopen-2019-032558 (PMC7003378; doi:10.1136/bmjopen-2019-032558)
Supplement: Supplementary data [file bmjopen-2019-032558supp002.pdf]

Expert Survey: Antenatal Care Service Quality

INTRODUCTION:
We invite you to participate in a brief online survey as part of a study aiming to assess the relationship between structural quality and process quality for antenatal care (ANC) services in low- and middle-income countries being conducted at the Johns Hopkins Bloomberg School of Public Health. You have been identified as an expert in maternal health with experience working in low- and middle-income countries and as such we would greatly appreciate your contribution to this work.

In order to explore the relationship between structural quality and process quality for ANC services, we first need to develop ANC quality of care metrics. While there are many definitions and frameworks for defining quality of care, there is a lack of consensus on indicators for measuring the quality of antenatal care. The goal of this survey is to elicit expert opinion on the items that are important to include in measures of ANC service quality. We expect that the results of this work will be published in a dissertation, and eventually a journal article.

The survey can be accessed at the following link:
https://tinyurl.com/ExpertSurvey-ANC

The survey should take approximately 20-30 minutes to complete. We would greatly appreciate if you can respond to the survey by 10 September 2018. If you have any questions, please feel free to contact Ashley Sheffel (asheffel@jhu.edu) or Melinda Munos (mmunos@jhu.edu).

Many thanks in advance for your time and contribution.

CONSENT:
By checking “Yes” I agree to participate in the survey. I understand the purpose and nature of this activity and I am participating voluntarily. I understand that I can stop taking the survey at any time, without any penalty or consequences. I understand that any personal information collected through this survey will remain confidential.

YES ☐ → continue NO ☐ → end survey

BACKGROUND:
In order to create a comprehensive list of items related to ANC service quality, a review of the Service Availability and Readiness Assessment Indicators and the WHO Focused Antenatal Care Guidelines was conducted along with a review of the data available in the Service Provision Assessment questionnaire, which is the main data source for this research. The identified items have been organized according to the dimensions of quality of care as proposed by the WHO Quality of Care Framework for Maternal and Newborn Health: essential physical resources, competent and motivated human resources, provision of care, and experience of care. Brief descriptions of each dimension are provided below.

DIMENSION DESRIPTIONS:
Physical resources: This dimension includes items related to the availability of an appropriate physical environment, equipment, supplies, medicines, and diagnostics required to provide ANC services.

Sheffel A, *et al. BMJ Open* 2019; 9:e032558. doi: 10.1136/bmjopen-2019-032558

Human resources: This dimension includes items related to the availability of competent, motivated human resources to provide ANC services.

Provision of care: This dimension includes items related to use of evidence-based practices, information systems in which record- keeping allows review, and functioning systems for referral between different levels of care.

Experience of care: This dimension includes items related to effective communication with women and their families about the care provided, their expectations and their rights; care with respect and preservation of dignity; and access to the social and emotional support of their choice.

INSTRUCTIONS: For each dimension, a set of items has been identified that are related to delivery of ANC. The goal is to identify the items which are most important for delivery of a high-quality ANC service ***within each dimension***. For each item, please provide a rating of importance of the item to ANC service quality based on your expert opinion. Circle only one response option per item. If you are unfamiliar with an item or don't know how to rate the item, please select "Don't know".

RATINGS:
1 = Unimportant/Non-informative for measuring ANC service quality
2= Somewhat important for measuring ANC service quality
3 = Very important for measuring ANC service quality
4 = Essential for measuring ANC service quality
98 = Don't know

Sheffel A, *et al. BMJ Open* 2019; 9:e032558. doi: 10.1136/bmjopen-2019-032558

#	Item Name	Response options				
		Unimportant / Non-informative	Somewhat important	Very important	Essential	Don't Know
ESSENTIAL PHYSICAL RESOURCES						
EQUIPMENT AND SUPPLIES<br>*Availability of…*						
1	Blood pressure apparatus	1	2	3	4	98
2	Examination light	1	2	3	4	98
3	Fetal stethoscope	1	2	3	4	98
4	Stethoscope	1	2	3	4	98
5	Adult weighing scale	1	2	3	4	98
6	Thermometer	1	2	3	4	98
7	Tape measure for fundal height	1	2	3	4	98
8	Height board	1	2	3	4	98
9	Speculum	1	2	3	4	98
10	Examination bed	1	2	3	4	98
11	Latex gloves	1	2	3	4	98
12	Single use syringes	1	2	3	4	98
13	Soap and water OR alcohol based hand rub	1	2	3	4	98
14	Disinfectant	1	2	3	4	98
15	Appropriate storage of sharps waste (sharps box)	1	2	3	4	98
16	Appropriate storage of infectious waste (pedal bin with lid and liner)	1	2	3	4	98
17	Safe final disposal of sharps (incineration)	1	2	3	4	98
18	Safe final disposal of infectious wastes (incineration)	1	2	3	4	98
19	Medical masks	1	2	3	4	98
20	Gowns	1	2	3	4	98
21	Eye protection	1	2	3	4	98
22	Gum boots	1	2	3	4	98
DIAGNOSTICS<br>*Availability on-site to test for…*						
23	Hemoglobin	1	2	3	4	98
24	Urine dipstick- protein	1	2	3	4	98
25	Urine dipstick- glucose	1	2	3	4	98
26	Grouping and Rhesus factor	1	2	3	4	98
27	Syphilis RDT / RPR	1	2	3	4	98

Sheffel A, *et al. BMJ Open* 2019; 9:e032558. doi: 10.1136/bmjopen-2019-032558

#	Item Name	Response options				
		Unimportant / Non-informative	Somewhat important	Very important	Essential	Don't Know
28	HIV testing / RDT	1	2	3	4	98
MEDICINES AND COMMODITIES<br>*Availability on-site of…*						
29	Iron tablets	1	2	3	4	98
30	Folic acid tablets	1	2	3	4	98
31	Tetanus toxoid vaccine	1	2	3	4	98
32	Intermittent Preventative Treatment (IPT) drug	1	2	3	4	98
33	Insecticide treated nets (ITNs) or vouchers	1	2	3	4	98
34	De-worming drugs (mebendazole / albendazole)	1	2	3	4	98
BASIC AMENITIES<br>*Availability of…*						
35	Power	1	2	3	4	98
36	Improved water source	1	2	3	4	98
37	Room with auditory and visual privacy	1	2	3	4	98
38	Sanitation facilities	1	2	3	4	98
39	Communication equipment	1	2	3	4	98
40	Computer with email/internet access	1	2	3	4	98
41	Emergency transportation	1	2	3	4	98
COMPETENT, MOTIVATED HUMAN RESOURCES						
42	Guidelines for ANC available at the facility	1	2	3	4	98
43	At least one health facility staff member trained in ANC in the last two years	1	2	3	4	98
44	At least 75% of staff offering ANC at the facility have received supervision in the last six months	1	2	3	4	98
45	At least 75% of staff offering ANC at the facility report availability of opportunities for promotion in their current job	1	2	3	4	98
PROVISION OF CARE						
HISTORY TAKING<br>*During ANC visit the provider asks about…*						
46	Personal history: client age	1	2	3	4	98
47	Personal history: medications client is taking	1	2	3	4	98
48	Personal history: date last menstrual period began	1	2	3	4	98
49	Personal history: any prior pregnancy	1	2	3	4	98
50	Family history	1	2	3	4	98

Sheffel A, *et al. BMJ Open* 2019; 9:e032558. doi: 10.1136/bmjopen-2019-032558

#	Item Name	Response options				
		Unimportant / Non-informative	Somewhat important	Very important	Essential	Don’t Know
51	Social history	1	2	3	4	98
52	Past medical history for prior pregnancies: still birth	1	2	3	4	98
53	Past medical history for prior pregnancies: infant died in the first week of life	1	2	3	4	98
54	Past medical history for prior pregnancies: heavy bleeding during or after delivery	1	2	3	4	98
55	Past medical history for prior pregnancies: previous assisted delivery	1	2	3	4	98
56	Past medical history for prior pregnancies: previous spontaneous abortion	1	2	3	4	98
57	Past medical history for prior pregnancies: multiple pregnancies	1	2	3	4	98
58	Past medical history for prior pregnancies: prolonged labor	1	2	3	4	98
59	Past medical history for prior pregnancies: pregnancy-induced hypertension	1	2	3	4	98
60	Past medical history for prior pregnancies: pregnancy related convulsions	1	2	3	4	98
61	Past medical history for prior pregnancies: high fever or infection during prior pregnancy	1	2	3	4	98
62	History of complaints in current pregnancy: vaginal bleeding	1	2	3	4	98
63	History of complaints in current pregnancy: fever	1	2	3	4	98
64	History of complaints in current pregnancy: headache or blurred vision	1	2	3	4	98
65	History of complaints in current pregnancy: swollen face or hands or extremities	1	2	3	4	98
66	History of complaints in current pregnancy: tiredness or breathlessness	1	2	3	4	98
67	History of complaints in current pregnancy: fetal movement (loss of, excessive, normal)	1	2	3	4	98
68	History of complaints in current pregnancy: cough or difficulty breathing for three weeks or longer	1	2	3	4	98
69	History of complaints in current pregnancy: amniotic leakage	1	2	3	4	98
EXAMINATION<br>*During ANC visit the provider examines the client for…*						
70	Head to toe (whole body)	1	2	3	4	98
71	Pallor	1	2	3	4	98
72	Oedema	1	2	3	4	98
73	Breast	1	2	3	4	98
74	Lungs and heart	1	2	3	4	98
OBSERVATION AND CLINICAL INVESTIGATION<br>*During ANC visit the provider assesses the client’s…*						

Sheffel A, *et al. BMJ Open* 2019; 9:e032558. doi: 10.1136/bmjopen-2019-032558

#	Item Name	Response options				
		Unimportant / Non-informative	Somewhat important	Very important	Essential	Don't Know
75	Blood pressure	1	2	3	4	98
76	Weight	1	2	3	4	98
77	Temperature	1	2	3	4	98
78	Pulse	1	2	3	4	98
OBSTETRIC COMPLICATIONS<br>*During ANC visit the provider assesses the client for potential obstetric complications by…*						
79	Palpating the client's abdomen for fundal height	1	2	3	4	98
80	Palpating the client's abdomen for fetal presentation	1	2	3	4	98
81	Listening to the client's abdomen for fetal heartbeat	1	2	3	4	98
82	Conducting an ultrasound/ referring client for ultrasound/ look at recent ultrasound report	1	2	3	4	98
PELVIC EXAMINATION<br>*During ANC visit the provider conducts a pelvic exam with…*						
83	Soft tissue assessment/ Vaginal examination	1	2	3	4	98
84	Bony pelvic assessment	1	2	3	4	98
LABORATORY INVESTIGATIONS<br>*During ANC visit the provider performs or refers the client to test for…*						
85	Hemoglobin	1	2	3	4	98
86	Grouping and rhesus factor	1	2	3	4	98
87	Syphilis / RPR	1	2	3	4	98
88	HIV	1	2	3	4	98
89	Urine – protein	1	2	3	4	98
90	Urine -sugar	1	2	3	4	98
91	Urine- acetone	1	2	3	4	98
DRUG ADMINISTRATION AND IMMUNIZATION<br>*During ANC visit the provider administers or prescribes…*						
92	Iron and/or folic acid	1	2	3	4	98
93	Antimalarials	1	2	3	4	98
94	Tetanus toxoid	1	2	3	4	98
CLIENT EDUCATION AND COUNSELING<br>*During ANC visit the provider counsels the client on…*						
95	Process of pregnancy and its complications	1	2	3	4	98
96	Diet and nutrition	1	2	3	4	98
97	Rest and exercise in pregnancy	1	2	3	4	98
98	Personal hygiene	1	2	3	4	98

Sheffel A, *et al. BMJ Open* 2019; 9:e032558. doi: 10.1136/bmjopen-2019-032558

#	Item Name	Response options				
		Unimportant / Non-informative	Somewhat important	Very important	Essential	Don't Know
99	Danger signs in pregnancy	1	2	3	4	98
100	Use of drugs in pregnancy	1	2	3	4	98
101	Effects of STI/HIV/AIDS	1	2	3	4	98
102	Voluntary counselling and testing for HIV	1	2	3	4	98
103	Care of breasts	1	2	3	4	98
104	Breastfeeding	1	2	3	4	98
105	Symptoms/signs of labor	1	2	3	4	98
106	Plans of delivery (emergency preparedness, place of delivery, transportation, financial arrangements)	1	2	3	4	98
107	Plans for postpartum care	1	2	3	4	98
108	Family planning	1	2	3	4	98
109	Harmful habits (e.g. smoking, drug abuse, alcoholism)	1	2	3	4	98
110	Schedule of return visit	1	2	3	4	98
EXPERIENCE OF CARE						
111	Client is able to discuss problems or concerns with provider	1	2	3	4	98
112	Client satisfied with the amount of explanation received about the problem or treatment	1	2	3	4	98
113	Client satisfied with how the staff treated them	1	2	3	4	98
114	Privacy from having others see the consultation	1	2	3	4	98
115	Privacy from having others hear the consultation	1	2	3	4	98
116	Client satisfied with the wait time	1	2	3	4	98
117	Client satisfied with the number of days services are available at the facility	1	2	3	4	98
118	Client satisfied with the hours of service at the facility	1	2	3	4	98
119	Client satisfied with the cost for services or treatments	1	2	3	4	98
120	Client satisfied with the availability of medicines at the facility	1	2	3	4	98
121	Client satisfied with the cleanliness of the facility	1	2	3	4	98

Are there any items you feel are essential for delivering a high-quality ANC service that do not appear in this questionnaire?

____

____

Sheffel A, *et al. BMJ Open* 2019; 9:e032558. doi: 10.1136/bmjopen-2019-032558

____
____
____

RESPONDENT INFORMATION:
Please answer the following question about yourself and your current role. This information will only be used for internal purposes and will not be disseminated.

1. Name: ___________________________
2. Email address: ___________________________
3. Organization: ___________________________
4. Position title: ___________________________
5. Years of experience in maternal health: ___________________________

The survey is now complete. Thank you for helping us with our research. We greatly appreciate your time and insights.

Sheffel A, *et al. BMJ Open* 2019; 9:e032558. doi: 10.1136/bmjopen-2019-032558
