# Supplementary material for: Development of summary indices of antenatal care service quality in Haiti, Malawi and Tanzania
Source: BMJ Open. 2019 Dec 2;9(12):e032558. doi: 10.1136/bmjopen-2019-032558 (PMC7003378; doi:10.1136/bmjopen-2019-032558)
Supplement: Author's manuscript [file bmjopen-2019-032558.draft_revisions.pdf]

BMJ Open is committed to open peer review. As part of this commitment we make the peer review history of every article we publish publicly available.

When an article is published we post the peer reviewers' comments and the authors' responses online. We also post the versions of the paper that were used during peer review. These are the versions that the peer review comments apply to.

The versions of the paper that follow are the versions that were submitted during the peer review process. They are not the versions of record or the final published versions. They should not be cited or distributed as the published version of this manuscript.

BMJ Open is an open access journal and the full, final, typeset and author-corrected version of record of the manuscript is available on our site with no access controls, subscription charges or pay-per-view fees (http://bmjopen.bmj.com).

If you have any questions on BMJ Open's open peer review process please email info.bmjopen@bmj.com

BMJ Open

Development of Summary Indices of Antenatal Care Service Quality in Haiti, Malawi, and Tanzania

Journal:	*BMJ Open*
Manuscript ID	bmjopen-2019-032558
Article Type:	Research
Date Submitted by the Author:	24-Jun-2019

Complete List of Authors:	Sheffel, Ashley; Johns Hopkins University Bloomberg School of Public Health, Department of International Health<br>Munos, Melinda; Johns Hopkins University Bloomberg School of Public Health, Department of International Health<br>Heidkamp, Rebecca; Johns Hopkins University Bloomberg School of Public Health, International Health<br>Zeger, Scott; Johns Hopkins University Bloomberg School of Public Health, Department of Biostatistics; Johns Hopkins University Bloomberg School of Public Health, Department of International Health

Keywords:	Maternal medicine < OBSTETRICS, International health services < HEALTH SERVICES ADMINISTRATION & MANAGEMENT, Quality in health care < HEALTH SERVICES ADMINISTRATION & MANAGEMENT, PUBLIC HEALTH, STATISTICS & RESEARCH METHODS

SCHOLARONE™ Manuscripts

Title: Development of Summary Indices of Antenatal Care Service Quality in Haiti, Malawi, and Tanzania

Authors: Ashley Sheffel[1], Scott Zeger[1,2], Rebecca Heidkamp[1], Melinda Munos[1]

Affiliations:

[1]Department of International Health, Johns Hopkins Bloomberg School of Public Health, Baltimore, MD, USA

[2]Department of Biostatistics, Johns Hopkins Bloomberg School of Public Health, Baltimore, MD, USA

Author contributions: AS, MM, RH, and SZ contributed to conceptualizing the paper and analysis; AS prepared the manuscript; MM, RH, and SZ critically reviewed and revised the manuscript.

Corresponding author:

Ashley Sheffel

Johns Hopkins Bloomberg School of Public Health

615 N Wolfe St. Room E8009

Baltimore, MD 21205 USA

asheffel@jhu.edu

Keywords: Maternal medicine, International health services, Quality in health care, Public health, Statistics & research methods

Word count: 4,961

Reference count: 32

Table/Figure count: 5 tables, 3 figures

For peer review only

For peer review only - http://bmjopen.bmj.com/site/about/guidelines.xhtml

Funding: This work was supported by the Improving Measurement and Program Design grant (OPP1172551) from the Bill & Melinda Gates Foundation.

Abbreviations:

ANC – Antenatal Care

CoV – Coefficient of Variation

DHS – Demographic and Health Survey

FANC – Focused Antenatal Care

IQR – Inter-Quartile Range

LMIC – Low-Income and Middle- Income Countries

PCA – Principal Components Analysis

SPA – Service Provision Assessment

USAID – US Agency for International Development

For peer review only

ABSTRACT

Introduction: Measuring quality of care in low-income and middle-income countries is complicated by the lack of a standard, universally-accepted definition for "quality" for any particular service, as well as limited guidance on which indicators to include in measures of quality of care, and how to incorporate those indicators into summary indices. The aim of this paper is to develop, characterize, and compare a set of antenatal care (ANC) indices for facility readiness and provision of care.

Methods: We created nine indices for facility readiness using three methods for selecting items and three methods for combining items. In addition, we created three indices for provision of care using one method for selecting items and three methods for combining items. For each index, we calculated descriptive statistics, categorized the continuous index scores using tercile cut points to assess comparability of facility classification, examined the variability and distribution of scores, assessed floor and ceiling effects, and assessed the inclusion of items with a range of variability.

Results: Our results showed that within a country the indices were quite similar in terms of mean index score, facility classification, coefficient of variation, floor and ceiling effects, and the inclusion of items in an index with a range of variability. Notably, the indices created using PCA to combine the items were the most different from the other

For peer review only - http://bmjopen.bmj.com/site/about/guidelines.xhtml

indices. In addition, the core weighted index had lower agreement with the other indices when looking at facility classification.

Conclusions: As improving quality of care becomes integral to global efforts to produce better health outcomes, demand for guidance on creating standardized measures of service quality will grow. This study provides health systems researchers with a comparison of methodologies commonly used to create summary indices of ANC service quality and it highlights the similarities and differences between methods.

For peer review only

For peer review only - http://bmjopen.bmj.com/site/about/guidelines.xhtml

Strengths and Limitations of this Study

- To our knowledge, this is the first study that uses multiple methodologies to develop and compare indices for ANC facility readiness and provision of care.
- Our approaches to item selection and combination were based on the most commonly used approaches in the literature and we included those most likely to be useful to LMICs.
- The analysis was conducted using data from multiple countries which strengthens our ability to generalize the findings to other similar contexts.
- The SPA survey, while quite comprehensive, does not capture every aspect of quality of care that may be important to include in quality of care metrics. However, the SPA represents the most comprehensive information on quality of care in LMICS that is currently available.
- This analysis does not provide a formal validation of any one ANC quality of care index. However, it does characterize various indices of ANC summary indices and provides valuable information on the similarities and differences between indices.

For peer review only

For peer review only - http://bmjopen.bmj.com/site/about/guidelines.xhtml

INTRODUCTION

Reducing maternal morbidity and mortality has been a global priority over the last several decades. While progress has been made in both improving coverage and reducing mortality, there remains an unacceptably high number of deaths of women and children globally [1]. In many countries, considerable increases in the coverage of health services have not translated into sufficient reductions in mortality [2]. This finding suggests that quality of care may play a critical role in producing better health outcomes. Reducing maternal mortality will require not only increasing the coverage of health interventions, but also ensuring that those interventions are delivered with a high level of quality [3 4]. These interventions include not only intrapartum and postpartum care, but also antenatal care (ANC), which is important to maintaining a healthy pregnancy [5-7], and promotes safe delivery, postnatal attendance, and is positively associated with an increase in facility-based deliveries [8-10].

For low-income and middle-income countries (LMICs) to systematically and comprehensively measure quality of care, they require a definition of quality of care and clear, specific indicators to operationalize the definition. Many quality of care frameworks exist, most of which build from Donabedian's 1988 framework characterizing quality at three levels: structure, commonly called facility readiness (the setting in which care occurs including material resources, human resources, and organizational structure), process (commonly called provision of care — the quality of

For peer review only - http://bmjopen.bmj.com/site/about/guidelines.xhtml
For peer review only

medical advice delivered by providers to clients, as well as interpersonal relationships between the provider and the client), and outcome (the effects of care on the health status and behaviors of patients, as well as improvements in patient knowledge and the degree of satisfaction with care) [11 12]. While there may be growing consensus on the core components of a framework for describing quality of care, measuring quality of care is complicated by lack of a standard, universally accepted definition for "quality" for any one particular service [13-15]. In addition, there is limited guidance on which indicators to include in measures of quality of care and how to incorporate those indicators into summary indices.

Studies on quality of care in LMICs largely rely on data from health facility surveys that collect data on facility readiness and provision of care. These studies often use summary indices to provide an overall description of service quality. However, there is little consistency between studies in terms of the items included in summary metrics of quality of care, or in how the metrics are created. ***Supplementary Table 1*** shows studies that used data from the Service Provision Assessment (SPA) to create quality of care metrics for ANC, each using a different approach. Item selection was guided by different sources including the Donabedian quality of care framework, clinical guidelines, and various definitions of quality of care. The number of items that were included in an index varied from a minimum of seven to a maximum of forty, and various methods were used to combine items into indices. While many of these studies

For peer review only - http://bmjopen.bmj.com/site/about/guidelines.xhtml

had some overlap in the items selected for inclusion in their ANC quality of care index, the quality of care indices varied greatly in the items selected for inclusion in the indices and in the methodology utilized for combining items into a summary measure.

As quality of care indices are increasingly used by countries, global agencies, and researchers to quantify the quality of health services and estimate effective coverage, there is a need to standardize the methodology for creating these measures. To date, few studies have compared the methodologies used to create indices of ANC service quality. The objective of this paper is to develop, characterize, and compare a set of facility readiness and provision of care indices for ANC.

METHODS

Data

Service Provision Assessment (SPA)

The Service Provision Assessment (SPA) is a health facility assessment widely used in LMICs to generate nationally representative data on health service delivery [16]. SPA data are publicly available through the Demographic and Health Survey (DHS) program. The SPA includes a standard set of survey instruments: a facility inventory questionnaire, health worker interviews, observation of ANC consultations, and exit interviews with ANC clients.

In this analysis we included data from all SPA surveys conducted in the last five years (i.e. since 2013) that included observations of ANC consultations (two surveys

For peer review only - http://bmjopen.bmj.com/site/about/guidelines.xhtml

excluded) and for which recode data files were available (three surveys excluded). The included surveys are from Haiti (2013), Malawi (2013/14), and Tanzania (2014/15). The SPA final country reports contain comprehensive information on the survey methodology and questionnaires [17-19]. Briefly described, the survey in Tanzania was a nationally representative sample of health facilities. Health facilities were selected using stratified systematic probability sampling with stratification by region and facility type (with oversampling of some facility types such as hospitals). In Haiti and Malawi, the survey was a census of all health facilities in the country. In all three countries, all surveyed facilities completed the facility inventory module. In addition, within each facility, up to eight health workers were interviewed including all health workers whose consultations were observed and those who provided information for any section of the facility questionnaire. ANC clients were selected for observation using systematic sampling, based on the number of clients present at each service site on the day of the visit. At facilities where the number of ANC clients expected on the day of the survey could not be predetermined, the sample was opportunistic since clients were selected as they arrived. Observation was completed for a minimum of five clients per service provider, with a maximum of 15 observations in any given facility for each service. Client exit interviews were conducted with every client whose visit was observed.

Maternal Health Expert Survey

For peer review only

For peer review only - http://bmjopen.bmj.com/site/about/guidelines.xhtml

To identify elements of facility readiness and the provision of ANC considered by experts to be the most important to ANC service quality, we conducted a survey of maternal health experts who had experience working in LMICs. We reviewed the SARA indicators and the WHO Focused Antenatal Care (FANC) guidelines, as well as the SPA questionnaire. This process identified a total of 121 items organized according to the dimensions of quality of care proposed by the WHO Quality of Care Framework for maternal and newborn health: essential physical resources (41 items), competent and motivated human resources (four items), provision of care (65 items), and experience of care (11 items) [20].

A group of 50 experts in maternal health with experience working in LMICs were asked to complete the online Google survey. Respondents rated each item based on overall importance. Importance ratings ranged from one (item was unimportant) to four (item was essential). Experts also provided a list of items they felt were important for delivering high-quality ANC services, but were missing from the survey instrument. Fifteen maternal health experts completed the survey, with respondents representing academic institutions, donor agencies, United Nations agencies, global health implementing organizations, and Ministries of Health in LMICs.

Analysis

For the facility ready analysis, we limited this analysis to facilities offering ANC services and with at least one first visit ANC client observation. In order to standardize

For peer review only - http://bmjopen.bmj.com/site/about/guidelines.xhtml

expected clinical actions, we limited the provision of care analysis to women attending the health facility for their first ANC visit. Finally, we excluded cases with incomplete data for any variables of interest.

Facility Readiness and Provision of Care Indices

We created nine facility readiness indices using three methods for selecting items (a "core set" of items, "expert survey" set of items, and "maximum" set of items) and three methods for combining items (simple additive, weighted additive, and PCA) (***Figure 1***). Data for these indices comes from the facility audit questionnaire and all items were created as facility-level indicators.

We created three indices for provision of care using one method for selecting items ("expert survey" set of items) and three methods for combining items (simple additive, weighted additive, and PCA) (***Figure 1***). Data for these indices comes from the client/provider observation and client exit interview questionnaires and all items were created as client-level indicators.

Item Selection

All items eligible for inclusion in the indices were derived from the comprehensive list of items related to ANC service quality developed for the maternal health expert survey. Items that were not collected across SPA surveys in all three countries and items that are not required for a first ANC visit were excluded from all indices. For each readiness item, a binary score was created based on whether the criteria for availability were met on the day of the survey (1=available, 0=not available).

For peer review only

For peer review only - http://bmjopen.bmj.com/site/about/guidelines.xhtml

For human resources items, each item is a proportion ranging from 0 to 1. For provision of care items, a binary score was created based on whether or not the activity was conducted during the ANC visit (1=conducted, 0=not conducted) or whether or not the client had a problem with the item during the ANC visit (1=no problem, 0=major or minor problem). The list of items included in each readiness and provision of care index can be found in ***Supplementary Table 2 and Supplementary Table 4***, respectively.

Three methods were used to select items to include in the facility readiness indices. The first method identified the **core set** of readiness items required to deliver ANC services. This set of items was identified by reviewing the provision of care items required for an ANC visit based on the WHO FANC guidelines and by determining the human resources, equipment and supplies, medicines, and diagnostics required to deliver each specific item. In creating the core set of items for facility readiness, we mapped each provision of care item to the facility readiness items required to deliver the specific service component. We found that of the 49 provision of care items, 36 items required only human resources and 13 items required human resources plus equipment, diagnostics, medicines, or basic amenities. The core index did not include standard precautions for infection prevention items because these are not explicitly required for any one provision of care item. A total of 21 items were selected for the core set readiness index.

For peer review only - http://bmjopen.bmj.com/site/about/guidelines.xhtml

The second method utilized results from the **expert survey** to identify the set of readiness items maternal health experts identified as essential to deliver ANC services. Mean ratings were calculated for each readiness item (see ***Supplementary Table 3*** for results). Items rated by the expert group with a mean importance of >3.4 out of 4 were selected for the expert survey index. The threshold for inclusion was determined by examining the distribution of scores and identifying a natural break point which separated the top-rated items from the rest. In addition, items were selected so that at least one item per domain (human resources, equipment and supplies, diagnostics, medicines, basic amenities) was included in the index. Of the 45 facility readiness items identified for inclusion in the expert survey, only four were related to human resources and of those, only one was highly rated by the expert group as essential for high quality service delivery. The expert survey respondents mentioned a number of readiness items they felt were essential for high quality care, but were missing from our survey and from SPA datasets. A total of 19 items were selected, representing 42% of the total items for the expert survey readiness index.

The third method identified the **maximum set** of readiness items used to deliver ANC services. This index includes all items identified in the SPA related to ANC readiness across the following domains: human resources, equipment and supplies, medicines, diagnostics, and basic amenities. A total of 38 items were selected for the maximum set readiness index.

For peer review only - http://bmjopen.bmj.com/site/about/guidelines.xhtml

We utilized a single method to select items to include in the provision of care index. The method utilized results from the **expert survey** to select the set of provision of care items maternal health experts identified as essential to deliver ANC services. We chose this method because maternal health experts were the best source for determining which processes are essential to high quality ANC consultations. Mean ratings were calculated for each provision of care item (see ***Supplementary Table 3*** for results). Items rated by the expert group with a mean importance of ≥3.0 out of 4 were selected for the provision of care index. The threshold for inclusion was decided by examining the distribution of scores which showed a bimodal distribution; then, selecting all items in the first mode. The expert survey respondents mentioned a number of provision of care items, largely related to experience of care, that they felt were essential for high quality care, but were missing from our survey and from SPA datasets. A total of 49 items were selected representing 64% of the total items for the provision of care index.

Item Combination

Three methods were used to combine items to create the facility readiness and provision of care indices — simple additive, weighted additive, and principal components analysis (PCA).

Simple additive: The simple additive index score was calculated by taking a sum of the items available divided by the total number of items in the index. The score was

For peer review only - http://bmjopen.bmj.com/site/about/guidelines.xhtml

scaled to zero to 100 by multiplying by 100. The simple additive index weighted all items in the index equally.

Weighted additive: The weighted additive index was also calculated as a sum of items, but instead of assuming equal weights for all items, the weighted additive index accounted for the number of items within each domain. Readiness items were first grouped into five domains: human resources, equipment and supplies, medicines, diagnostics, and basic amenities. For the provision of care index, items were initially grouped into five domains: history and counselling, examination, diagnostics, preventative treatment, and client experience. We then computed a domain score by adding the items within each domain and dividing by the total number of items in the domain. Finally, we averaged the domain scores and multiplied by 100 to scale the score from zero to 100.

Principal components analysis (PCA): To create the PCA index score, we conducted an unrotated, unweighted PCA using a correlation matrix and used the factor loadings from the first principal component to create the index score. We rescaled the score obtained from the first component of the PCA to a range of zero to 100 for comparability with other indices.

Statistical Analysis

We calculated descriptive statistics on each of the indices including mean, median, minimum, maximum, and range. In addition, for the indices created using PCA

For peer review only - http://bmjopen.bmj.com/site/about/guidelines.xhtml
For peer review only

to combine items, we calculated factor loadings, the eigenvalue, the percent of total variance explained by the first component, and Cronbach's alpha as a measure of reliability. Next, to compare scores from the nine facility readiness indices, we categorized the continuous index scores into low, medium, and high readiness categories using tercile cut points. We then assessed the comparability of the facility classification across the indices by calculating the percent agreement and Cohen's kappa, which accounts for the possibility of the agreement occurring by chance, between each combination of indices. Cohen's kappa ranges from -1 to 1 and can be interpreted as follows: < 0 no agreement, 0–0.20 slight, 0.21–0.40 fair, 0.41–0.60 moderate, 0.61–0.80 good, and 0.81–1 almost perfect agreement [21].

Next, we examined the variability and distribution of the index scores. The coefficient of variation (CoV) was used to measure the variation captured by each index, with a higher CoV indicative of more variation captured in the index. Distribution of the scores was examined by assessment of floor and ceiling effects. Floor and ceiling effects were considered to be present if more than 15% of facilities achieved the lowest or highest possible index score (0% or 100%) respectively [22]. The presence of floor and ceiling effects indicate that indices have limited ability to differentiate facilities at very low and very high readiness levels. Finally, each index was assessed in terms of inclusion of items with a range of variability. It was preferable for indices to have more items that were less frequently available and fewer items that are more frequently available. For

For peer review only - http://bmjopen.bmj.com/site/about/guidelines.xhtml

each index, we tallied the number of items that were available in <30% of facilities, <40% of facilities, and >90% of facilities.

All statistical analyses were carried out using R version 3.5.1 [23]. The institutional review board at the Johns Hopkins Bloomberg School of Public Health determined this analysis to be exempt from human subjects review.

Patient and Public Involvement

It was not appropriate or possible to involve patients or the public in this work.

RESULTS

The final analytic sample consisted of 358 health facilities and 779 ANC clients in Haiti, 253 health facilities and 815 ANC clients in Malawi, and 632 health facilities and 1,681 ANC clients in Tanzania. The mean readiness score varied by index from 56.6 to 64.1 in Haiti, 52.3 to 69.6 in Malawi, and 61.2 to 75.9 in Tanzania (***Table 1, Figure 2***). Across all countries, there were no indices for which any facilities received the minimum score of zero and all countries had at least three indices for which some facilities received the maximum score of 100. In addition, in each country, the three indices that contained the same items — but used different methodologies for combining the items — generally had a similar median. For example, the core simple, core weighted, and core PCA median scores in Tanzania were 73.5, 72.1, and 73.9, respectively. Finally, the indices created using PCA resulted in a larger inter-quartile range (IQR), particularly for the core and expert item indices, across all three countries.

For peer review only - http://bmjopen.bmj.com/site/about/guidelines.xhtml

The mean provision of care score varied by index from 38.9 to 85.9 in Haiti, 35.8 to 53.1 in Malawi, and 40.4 to 58.0 in Tanzania (***Table 1, Figure 3***). The expert PCA index in Haiti was the only index in which any clients received the minimum score of zero and no countries had an index for which any clients received the maximum score of 100. The expert simple and expert weighted indices had similar median provision of care scores; however, the expert weighted index scores were higher than the simple index scores in all three countries. The expert PCA index scores had median provision of care scores which were very different from both the expert simple and expert weighted scores. For example, in Malawi the expert simple mean provision of care score was 46.9, the expert weighted mean provision of care score was 53.7, and the expert PCA mean provision of care score was 33.9.

Principal Components Analysis (PCA)

The results from the PCA analysis are presented in ***Table 2***. Across all countries, and all indices for facility readiness, the items with the highest loadings (absolute value greater than 0.4) differed by index and country. For the core item indices, the items that loaded the highest were related to diagnostics and human resources. For the expert item indices, the items that loaded the highest were related to diagnostics. For the maximum item indices, the items that loaded the highest were related to diagnostics and basic amenities. For all facility readiness indices in all countries, the percent of variance explained by the first principal component was low, ranging from 17.21% for

For peer review only - http://bmjopen.bmj.com/site/about/guidelines.xhtml

the core and maximum indices in Haiti to 19.49% and 20.38% for the expert set of items in Tanzania and Haiti, respectively. In addition, we found that some items such as tetanus toxoid vaccine, de-worming medications, and syphilis testing had negative loadings.

Across all countries for provision of care, the items with the highest loadings (absolute value greater than 0.4) differed by country. For Haiti, the items that loaded the highest were related to client experience. For Malawi and Tanzania, the items that loaded the highest were related to history-taking and counselling; although for each country, the highest loadings were largely on different items. For provision of care indices in all countries, the percent of variance explained by the first principal component was low, ranging from 9.87% in Malawi to 19.49% in Haiti. In addition, we found that items such as history-taking, client education and counselling on danger signs in pregnancy, and hemoglobin testing had negative loadings.

Comparability of the Facility Classification

Table 3 presents the results of the percent agreement and Cohen's kappa coefficient among the nine facility readiness indices. Across all countries, all readiness indices had fair or better agreement. In Haiti, there were three index combinations that had fair agreement (core weighted/core PCA; core weighted/expert PCA; core weighted/maximum PCA) while the remainder of the indices had moderate or better

For peer review only - http://bmjopen.bmj.com/site/about/guidelines.xhtml

agreement. In Malawi, there was one index combination that had fair agreement (core weighted/maximum simple) while the remainder of the indices had moderate or better agreement. In Tanzania, there were two index combinations that had fair agreement (core weighted/expert PCA; core weighted/maximum PCA) while the remainder of the indices had moderate or better agreement.

Variability and Distribution of the Index

The variability and distribution of the nine facility readiness indices are presented in ***Table 4***. In Haiti, the CoV ranged from 21.12 (maximum simple readiness index) to 35.62 (expert PCA). In Malawi, the CoV ranged from 18.96 (expert PCA) to 32.09 (core PCA). In Tanzania, the CoV ranged from 19.11 (core simple) to 23.62 (expert PCA). Across all countries and all indices, there were no floor effects. Ceiling effects were limited and far below the 15% threshold. The highest percentage of ceiling effects was 3.33% which was found in Tanzania in three indices (expert simple, expert weighted, and expert PCA).

Inclusion of Items Across a Range of Frequency

Table 5 presents the percentage of facilities in which each index item was available in order to assess the inclusion of items across a range of frequency. Across all countries, the maximum item index contained the greatest number of items available in less than 40% of facilities (10 items Haiti, 12 items Malawi, and nine items in Tanzania). In Tanzania, the expert index did not include any items available in less than 40% of facilities. Within countries, the core, expert, and maximum indices had a similar number

For peer review only - http://bmjopen.bmj.com/site/about/guidelines.xhtml

of items that were available in over 90% of facilities (four to five in Haiti, six to eight in Malawi, seven to nine in Tanzania).

Discussion

To our knowledge, this is the first study that uses multiple methodologies to develop and compare indices for ANC facility readiness and provision of care. Our results showed that, within a country, indices containing different items combined using different approaches were quite similar in terms of mean index score, facility classification, coefficient of variation, floor and ceiling effects, and inclusion of items with a range of variability. Although similar overall, we found that the indices created using PCA were the most different from the other indices. In addition, the core weighted index had lower agreement with the other indices when looking at facility classification. This may be the result of some domains in the core weighted index having few items, making each of those items more influential in the overall index score.

Our analysis highlighted the importance of competent, motivated human resources to all aspects of delivering high quality ANC services. Our approach to defining the core set of items highlighted the lack of metrics for human resources captured by facility surveys, as well as the inconsistency between what inputs are required for provision of care and how facility readiness is often defined. This is especially important considering there is a health workforce crisis globally which is particularly pronounced in LMICs [24]. Many countries are suffering from an absolute

For peer review only - http://bmjopen.bmj.com/site/about/guidelines.xhtml

shortage of health care workers. The health workers they do have are often poorly distributed within a country and many workers are deficient in skill mix and core competencies [25 26].

This study's findings also suggest that to provide more comprehensive measures of ANC quality some items could be added to SPAs and other facility surveys. The expert survey respondents mentioned a number of items that they felt were essential for high quality care, but were missing from our survey and from SPA datasets; these items included: ensuring that clients are treated with respect and without discrimination, having the option for clients to invite their partner to participate in the consultation, discussing identification of a birth companion of choice with the client, including family members in counselling sessions, providing counselling on newborn care practices, and ensuring clients understand the counselling information they receive. While the global community is moving toward more person-centered care, data collected through health facility assessments is generally deficient in experience of care measures [27]. This may be due to the lack of validated instruments available for measuring the experience of maternity care [28]. In addition, measures of patient experience from health facility assessments are limited and may be subject to courtesy bias which has been found to be particularly problematic for subjective questions regarding items such as treatment by staff and consultation quality, items of interest for measuring experience of care [29]. We therefore cannot be certain if these measures are actually representative of people's

For peer review only - http://bmjopen.bmj.com/site/about/guidelines.xhtml

experiences or if other types of questions would better capture experience of care. However, a number of recent studies have generated validated tools for measuring respectful maternity care; these may well provide a starting point for incorporating improved experience of care metrics in health facility assessments such as the SPA [30 31].

We also found that the indices differed in terms of their ease of construction and interpretation. The simple additive and weighted additive approaches were straightforward to construct because they required taking averages of items, while the PCA necessitated more complex analysis. In addition, the simple additive approach was the easiest to interpret since facilities with more items available had a higher quality score. The weighted additive approach was also relatively easy to interpret. However, item weighting whereby each domain receives an equal weight was based on a conceptual framework for quality which has not been formally validated. The PCA approach was perhaps the most difficult to interpret as the PCA score represents the linear combination of variables that explained the most variance in the data. As the loadings of each variable on the first component were used as the weights, and some items were negatively correlated and consequently had negative loadings; having all items present in a facility did not always result in the highest PCA score. In addition, the weights assigned by PCA reflected the variation in the data which was different across countries. As a result, PCA did not produce the same weights across different contexts. As we were working with a common definition for what an ANC visit should include

For peer review only

across country contexts, this variability in loadings resulted in concerns regarding the face validity and construct validity of the PCA indices. Overall, the low percent of total variance explained by the first principle component, diverging items with high loadings between countries, negatively correlated items, and the complexity in creating and interpreting the measure highlights concerns for the use of PCA for creating quality of care indices [32].

We note several limitations of the analysis. First, this analysis was conducted using data from three countries, two in Africa and one in Latin America, which may limit generalizability of findings to low-income countries globally. However, the findings were relatively consistent across the three countries and the two African countries are likely representative of Southern and Eastern Africa. Second, the SPA survey, while quite comprehensive, does not capture every aspect of quality of care that may be important to include in quality of care metrics. However, the SPA represents the most comprehensive information on quality of care in LMICS that is currently available. Third, our approaches to item selection and combination were based on the most commonly used approaches in the literature. However, there are other methods that may be helpful in determining which items to include in a quality of care index, such as latent class analysis, that we did not implemented. The selection of methods for this analysis included those most likely to be useful to LMICs. Finally, this analysis does not provide a formal validation of any one ANC quality of care index. However, it does characterize

For peer review only

For peer review only - http://bmjopen.bmj.com/site/about/guidelines.xhtml

various indices of ANC summary indices and provides valuable information on the similarities and differences between indices.

Conclusion

While the goal of this study was not to identify the best index for measuring quality of care, we did endeavor to characterize the various indices to make more information available in order to assist health systems researchers in choosing a methodology for creating quality of care indices. In addition, we found that different methodologies may be better suited for different purposes. For example, the simple additive approach using the maximum number of items may be easiest for Ministries of Health in LMICs to understand and implement. However, if there is particular interest in using facility readiness as a proxy for provision of care, it might be conceptually important to ensure that items align well between facility readiness and provision of care thus making the core set of items a good choice. In addition, while the weighted additive method aims to give equal weights to domains of facility readiness and provision of care, the domains do not have equal numbers of items. As such, if there are domains with few items measured, these items will have more influence than items in domains with many items measured, which may not reflect the underlying construct well.

Future research on the association between facility readiness and provision of care would be helpful to further characterize the quality of care indices and inform

For peer review only - http://bmjopen.bmj.com/site/about/guidelines.xhtml

selection of an index. As quality of care becomes integral to global efforts to improve health outcomes, demand for guidance on creating standardized measures of service quality will grow. This study provides health systems researchers with a comparison of methodologies commonly used to create summary indices of ANC service quality and highlights the similarities and differences between methods.

For peer review only

Acknowledgements

The authors wish to acknowledge the Bill & Melinda Gates Foundation for their support of this project. In addition, we would like to thank the maternal health experts who participated in the expert survey; we appreciate your time and willingness to share your expertise.

Declaration of Interests

We declare no competing interests.

Role of the Funding Source

The funder had no role in the study design, data collection, analysis and interpretation of data, or manuscript writing.

For peer review only

For peer review only - http://bmjopen.bmj.com/site/about/guidelines.xhtml

REFERENCES

1. Countdown to 2015: Maternal Newborn and Child Survival. Fulfilling the Health Agenda for Women and Children: The 2014 Report, 2014.
2. Souza JP, Gulmezoglu AM, Vogel J, et al. Moving beyond essential interventions for reduction of maternal mortality (the WHO Multicountry Survey on Maternal and Newborn Health): a cross-sectional study. *Lancet* 2013;381(9879):1747-55. doi: 10.1016/s0140-6736(13)60686-8 [published Online First: 2013/05/21]
3. Sobel HL, Huntington D, Temmerman M. Quality at the centre of universal health coverage. *Health Policy Plan* 2016;31(4):547-9. doi: 10.1093/heapol/czv095 [published Online First: 2015/10/01]
4. Kruk ME, Larson E, Twum-Danso NAY. Time for a quality revolution in global health. *The Lancet Global Health* 2016;4(9) doi: 10.1016/S2214-109X(16)30131-0
5. Villar J, Bergsjø P. Scientific basis for the content of routine antenatal care. I. Philosophy, recent studies, and power to eliminate or alleviate adverse maternal outcomes. *Acta obstetricia et gynecologica Scandinavica* 1997;76(1):1-14.
6. Campbell O, Graham W. Strategies for reducing maternal mortality: getting on with what works. *The Lancet* 2006;368(9543):1284-99. doi: 10.1016/S0140-6736(06)69381-1
7. Lassi ZS, Salam RA, Das JK, et al. Essential pre-pregnancy and pregnancy interventions for improved maternal, newborn and child health. *Reproductive Health* 2014;11(Suppl 1):S2-S2. doi: 10.1186/1742-4755-11-S1-S2
8. Callaghan-Koru JA, McMahon SA, Chebet JJ, et al. A qualitative exploration of health workers' and clients' perceptions of barriers to completing four antenatal care visits in Morogoro Region, Tanzania. *Health policy and planning* 2016;31(8):1039-49. doi: 10.1093/heapol/czw034

For peer review only

For peer review only - http://bmjopen.bmj.com/site/about/guidelines.xhtml

9. Gross K, Schellenberg Armstrong J, Kessy F, et al. Antenatal care in practice: an exploratory study in antenatal care clinics in the Kilombero Valley, south-eastern Tanzania. *BMC Pregnancy and Childbirth* 2011;11:36:1-11.

10. Gupta S, Yamada G, Mpembeni R, et al. Factors associated with four or more antenatal care visits and its decline among pregnant women in Tanzania between 1999 and 2010. *PLoS ONE* 2014;9(7) doi: 10.1371/journal.pone.0101893

11. Donabedian A. The Quality of Care: How Can It Be Assessed? *JAMA* 1988;260(12):1743-43. doi: 10.1001/jama.1988.03410120089033

12. Donabedian A. Evaluating the quality of medical care. *The Milbank quarterly* 2005;83(4):691-729. doi: 10.1111/j.1468-0009.2005.00397.x

13. Donabedian A. The Definition of Quality and Approaches to Its Assessment Vol 1. Explorations in Quality Assessment and Monitoring. Ann Arbor, Michigan: Health Administration Press 1980.

14. Institute of Medicine Committee on Quality of Health Care in America. Crossing the quality chasm : a new health system for the 21st century. Washington, DC: National Academy Press 2001.

15. World Health Organization. Quality of care: a process for making strategic choices in health systems. Geneva, Switzerland 2006.

16. ICF. The DHS Program: SPA Overview 2017 [Available from: http://dhsprogram.com/What-We-Do/Survey-Types/SPA.cfm.

17. Institut Haïtien de l'Enfance, ICF International. Évaluation de Prestation des Services de Soins de Santé, Haïti, 2013, 2014.

18. Malawi Ministry of Health, ICF International. Malawi Service Provision Assessment (MSPA) 2013-2014: MoH and ICF International, 2014.

19. Tanzania Ministry of Health & Social Welfare, Tanzania National Bureau of Statistics, ICF International. Tanzania Service Provision Assessment Survey 2014-2015, 2016.

For peer review only

For peer review only - http://bmjopen.bmj.com/site/about/guidelines.xhtml

20. World Health Organization. Standards for improving quality of maternal and newborn care in health facilities. Geneva, Switzerland 2016.

21. Landis J, Koch G. The measurement of observer agreement for categorical data. *Biometrics* 1977;33(1):159-74.

22. Terwee CB, Bot SD, de Boer MR, et al. Quality criteria were proposed for measurement properties of health status questionnaires. *J Clin Epidemiol* 2007;60(1):34-42. doi: 10.1016/j.jclinepi.2006.03.012 [published Online First: 2006/12/13]

23. R: A Language and Environment for Statistical Computing [program]. Vienna, Austria: R Foundation for Statistical Computing, 2017.

24. Chen L, Evans T, Anand S, et al. Human resources for health: overcoming the crisis. *The Lancet* 2004;364(9449):1984-90.

25. Joint Learning Initiative. Human resources for health: overcoming the crisis. Cambridge, MA: Global Equity Initiative, Harvard University, 2004.

26. Ranson MK, Chopra M, Atkins S, et al. Priorities for research into human resources for health in low- and middle-income countries. *Bull World Health Organ* 2010;88(6):435-43. doi: 10.2471/blt.09.066290 [published Online First: 2010/06/12]

27. Sheffel A, Heidkamp R, Mpembeni R, et al. Understanding client and provider perspectives of antenatal care service quality: a qualitative multi-method study from Tanzania. *The Journal of Global Health* 2019; [in press]

28. Bohren MA, Vogel JP, Hunter EC, et al. The Mistreatment of Women during Childbirth in Health Facilities Globally: A Mixed-Methods Systematic Review. *PLoS Med* 2015;12(6) doi: 10.1371/journal.pmed.1001847 [published Online First: 2015/07/01]

29. Glick P. How reliable are surveys of client satisfaction with healthcare services? Evidence from matched facility and household data in Madagascar. *Soc Sci Med*

For peer review only - http://bmjopen.bmj.com/site/about/guidelines.xhtml

2009;68(2):368-79. doi: 10.1016/j.socscimed.2008.09.053 [published Online First: 2008/11/26]

30. Vedam S, Stoll K, Martin K, et al. The Mother's Autonomy in Decision Making (MADM) scale: Patient-led development and psychometric testing of a new instrument to evaluate experience of maternity care. *PLoS One* 2017;12(2):e0171804. doi: 10.1371/journal.pone.0171804 [published Online First: 2017/02/24]

31. Vedam S, Stoll K, Rubashkin N, et al. The Mothers on Respect (MOR) index: measuring quality, safety, and human rights in childbirth. *SSM - Population Health* 2017;3:201-10. doi: 10.1016/j.ssmph.2017.01.005

32. Sharker MY, Nasser M, Abedin J, et al. The risk of misclassifying subjects within principal component based asset index. *Emerging themes in epidemiology* 2014;11:6. doi: 10.1186/1742-7622-11-6 [published Online First: 2014/07/06]

For peer review only

For peer review only - http://bmjopen.bmj.com/site/about/guidelines.xhtml

TABLES

Table 1: Descriptive Statistics of Facility Readiness and Provision of Care Indices

	Facility Readiness Indices									Provision of Care Indices		
	core simple	core weighted	core PCA	expert simple	expert weighted	expert PCA	maximum simple	maximum weighted	maximum PCA	simple	weighted	PCA
**Haiti (N = 358 facilities)**										**Haiti (N = 779 clients)**		
mean	63.8	64.0	59.4	64.1	61.3	58.2	57.8	56.6	59.8	38.9	45.0	85.9
SE.mean	0.7	0.8	1.1	0.8	0.9	1.1	0.7	0.7	0.8	0.3	0.4	0.6
median	63.3	65.5	59.4	64.3	61.3	57.5	57.9	57.4	60.6	38.8	45.4	91.7
min	23.8	16.4	17.6	17.1	18.1	12.9	27.6	20.1	23.4	12.2	14.0	0.0
max	92.9	93.8	99.8	100.0	100.0	100.0	87.5	87.7	95.2	79.6	76.3	96.0
IQR	19.8	20.7	40.9	24.6	24.2	39.5	17.8	20.2	24.6	8.5	12.9	7.1
**Malawi (N = 253 facilities)**										**Malawi (N = 815 facilities)**		
mean	62.9	65.2	52.3	68.7	67.6	69.6	61.3	61.0	63.1	47.5	53.1	35.8
SE.mean	0.8	0.8	1.1	0.8	0.8	0.8	0.7	0.7	0.8	0.3	0.4	0.5
median	61.9	66.4	46.4	68.4	67.8	66.3	59.9	60.0	59.8	46.9	53.7	33.9
min	33.3	30.0	24.5	38.2	34.3	38.9	35.5	32.7	37.0	22.4	22.9	10.7
max	100.0	100.0	100.0	100.0	100.0	100.0	92.8	94.3	100.0	72.0	75.5	70.8
IQR	19.0	16.7	20.5	17.5	16.9	16.5	16.7	14.1	18.7	14.3	13.5	24.5
**Tanzania (N = 632 facilities)**										**Tanzania (N = 1,681 facilities)**		
mean	71.4	71.0	70.2	75.9	71.8	74.7	61.2	62.8	64.1	50.8	58.0	40.4
SE.mean	0.5	0.6	0.6	0.6	0.6	0.7	0.5	0.5	0.6	0.3	0.3	0.3
median	73.5	72.1	73.9	78.9	73.2	78.4	61.8	63.9	64.9	50.5	58.6	39.5
min	23.8	18.6	20.4	28.9	28.6	20.3	27.6	25.5	24.7	16.3	18.5	9.9
max	100.0	100.0	100.0	100.0	100.0	100.0	94.7	92.1	100.0	98.0	99.3	98.2
IQR	21.4	20.3	25.8	23.8	20.2	30.2	20.2	19.9	23.0	14.8	15.1	17.0

For peer review only - http://bmjopen.bmj.com/site/about/guidelines.xhtml

Table 2: Results from the Facility Readiness Indices and Provision of Care Indices Principal Component Analyses

**Facility Readiness Indices**									
**Item name**	**Haiti**			**Malawi**			**Tanzania**		
	**core set**	**expert set**	**maximum set**	**core set**	**expert set**	**maximum set**	**core set**	**expert set**	**maximum set**
Equipment and supplies									
Blood pressure apparatus	0.05	0.04	0.04	0.37	0.34	0.37	0.34	0.33	0.28
Examination light	0.08		0.14	0.44		0.49	0.34		0.38
Stethoscope	0.14	0.11	0.17	0.16	0.13	0.18	0.28	0.23	0.23
Adult weighing scale	0.22		0.19	0.25		0.32	0.43		0.39
Tape measure for fundal height	0.13		0.18	0.22		0.28	0.21		0.19
Examination bed	0.23	0.22	0.26	0.17	0.21	0.18	0.14	0.10	0.15
Latex gloves	0.24	0.52	0.49	0.14	0.32	0.39	0.08	0.50	0.51
Single use syringes		0.25	0.26		0.24	0.21		0.14	0.14
Soap and running water OR Alcohol based hand rub		0.41	0.41		0.38	0.40		0.52	0.57
Disinfectant		0.40	0.43		0.34	0.30		0.32	0.30
Appropriate storage of sharps waste (sharps box)			-0.12			0.09			0.11
Appropriate storage of infectious waste (pedal bin with lid)			0.01			0.03			0.17
Safe final disposal of sharps (incineration)			0.20			0.26			0.06
Safe final disposal of infectious wastes (incineration)			0.20			0.25			0.06
Medical masks			0.06			0.16			0.35
Gowns			0.31			0.21			0.38
Eye protection			0.29			0.28			0.23
Diagnostics									
Hemoglobin	0.63	0.61	0.60	0.75	0.75	0.70	0.72	0.71	0.69
Urine dipstick- protein	0.78	0.77	0.69	0.85	0.84	0.77	0.77	0.78	0.68
Urine dipstick- glucose	0.74	0.73	0.66	0.80	0.78	0.71	0.78	0.79	0.69
Grouping and Rhesus factor	0.18		0.19	0.53		0.47	0.29		0.37
Syphilis RDT / RPR	0.78	0.77	-0.04	0.68	0.68	-0.05	0.65	0.65	-0.04
HIV testing / RDT	0.78	0.79	0.72	0.18	0.23	0.21	0.41	0.41	0.35
Medicines									
Iron tablets	0.10	0.11	0.18	0.11	0.17	0.15	0.26	0.26	0.25
Folic acid tablets	0.15	0.16	0.19	0.16	0.24	0.22	0.20	0.21	0.21
TT vaccine	-0.05	-0.02	0.01	0.20	0.25	0.24	0.33	0.30	0.27
De-worming drugs	0.09		0.12	-0.12		-0.06	0.15		0.09
Basic Amenities									
Power		0.18	0.55		0.17	0.29		0.44	0.41
Improved water source		-0.08	0.23		0.10	0.14		0.03	0.39
Room with auditory and visual privacy	-0.10	0.46	-0.07	0.06	0.20	0.06	0.08	0.26	0.06
Sanitation facilities			0.42			0.10			0.25
Communication equipment			0.25			0.27			0.60
Computer with email/internet			0.62			0.71			0.66

For peer review only

For peer review only - http://bmjopen.bmj.com/site/about/guidelines.xhtml

Emergency transportation			0.38			0.01			0.33
Human resources									
Guidelines ANC	-0.06		0.00	0.15		0.20	0.38	0.33	0.36
Proportion of staff trained in ANC in last two years	0.43	0.39	0.39	0.47	0.46	0.44	0.40		0.34
Proportion of staff receiving supervision in the last six months	0.60		0.59	0.43		0.40	0.42		0.37
Proportion of staff reporting opportunities for promotion			0.17			-0.05			-0.07
**Eigenvalue**	3.61	3.87	3.61	3.74	3.42	3.74	3.66	3.70	3.66
**Percent of total variance**	17.21	20.38	17.21	17.80	17.98	17.80	17.44	19.49	17.44
**Cronbach's alpha**	0.64	0.70	0.75	0.66	0.65	0.72	0.70	0.71	0.78

Provision of Care Indices

Item name	Haiti	Malawi	Tanzania
History and counselling			
History-taking: Personal history: client age	0.03	0.24	0.28
History-taking: Personal history: medications client is taking	-0.02	0.05	0.22
History-taking: Personal history: date last menstrual period began	0.02	0.25	0.32
History-taking: Personal history: any prior pregnancy	-0.09	0.44	0.31
History-taking: Past medical history for prior pregnancies: still birth	-0.18	0.69	0.46
History-taking: Past medical history for prior pregnancies: infant died in the first week of life	-0.01	0.59	0.45
History-taking: Past medical history for prior pregnancies: heavy bleeding during or after delivery	0.03	0.73	0.50
History-taking: Past medical history for prior pregnancies: previous assisted delivery	-0.02	0.73	0.42
History-taking: Past medical history for prior pregnancies: previous spontaneous abortion	0.00	0.69	0.39
History-taking: Past medical history for prior pregnancies: previous multiple pregnancies	0.02	0.54	0.37
History-taking: Past medical history for prior pregnancies: previous prolonged labor	0.00	0.44	0.39
History-taking: Past medical history for prior pregnancies: pregnancy-induced hypertension	-0.13	0.73	0.35
History-taking: Past medical history for prior pregnancies: pregnancy related convulsions	0.00	0.68	0.37

For peer review only

History-taking: Past medical history for prior pregnancies: high fever or infection during prior pregnancy/pregnancies	-0.04	0.21	0.35
History-taking: History of complaints in current pregnancy: vaginal bleeding	0.01	0.31	0.58
History-taking: History of complaints in current pregnancy: fever	0.07	0.15	0.51
History-taking: History of complaints in current pregnancy: headache or blurred vision	0.03	0.18	0.56
History-taking: History of complaints in current pregnancy: swollen face or hands or extremities	0.03	0.25	0.48
History-taking: History of complaints in current pregnancy: tiredness or breathlessness	-0.02	0.17	0.51
History-taking: History of complaints in current pregnancy: fetal movement (loss of, excessive, normal)	0.04	0.10	0.47
History-taking: History of complaints in current pregnancy: cough or difficulty breathing for 3 weeks or longer	-0.04	0.21	0.36
Client education and counselling: Process of pregnancy and its complications	0.01	0.16	0.27
Client education and counselling: Diet and nutrition	0.03	0.20	0.28
Client education and counselling: Danger signs in pregnancy	-0.01	0.14	0.65
Client education and counselling: Voluntary counselling and testing for HIV	0.08	0.12	0.27
Client education and counselling: Breast feeding	-0.02	0.07	0.29
Client education and counselling: Plans of delivery (emergency preparedness, place of delivery, transportation, financial arrangements)	0.07	0.28	0.37
Examination			
Examination and observation: Edema (other than ankle-specify)	0.08	0.21	0.24
Examination and observation: Blood pressure	0.03	0.09	0.10
Examination and observation: Weight	0.06	0.11	0.16
Obstetric complications: Palpate the client's abdomen for fundal height	0.15	0.11	0.19
Diagnostics			
Laboratory investigations: Hemoglobin	0.03	-0.01	0.12
Laboratory investigations: Grouping and rhesus factor	0.04	0.15	0.16

For peer review only - http://bmjopen.bmj.com/site/about/guidelines.xhtml

Laboratory investigations: RPR (syphilis test)	0.03	0.04	0.16
Laboratory investigations: HIV testing	0.04	0.04	0.23
Laboratory investigations: Urine-protein, sugar, acetone	0.03	0.03	0.09
Preventative treatment			
Drug administration and immunization: Iron and or folic acid provided or prescribed	0.06	0.13	0.18
Drug administration and immunization: Tetanus toxoid provided or prescribed	0.06	0.03	0.09
Client experience			
No problem: discuss problems or concerns	0.65	-0.01	0.14
No problem: explanation you received	0.64	0.08	0.15
No problem: how the staff treated you	0.73	0.05	0.09
No problem: privacy from having others see	0.72	0.07	0.20
No problem: privacy from having others hear	0.74	0.10	0.20
No problem: time you waited	0.44	0.04	0.01
No problem: number of days services are available	0.73	0.01	0.11
No problem: hours of service	0.75	0.00	0.06
No problem: cost for services or treatments	0.65	0.10	0.08
No problem: availability of medicines	0.62	0.02	0.06
No problem: cleanliness of the facility	0.69	0.03	0.06
**Eigenvalue**	5.14	4.84	5.08
**Percent of total variance**	10.49	9.89	10.37
**Cronbach's alpha**	0.59	0.69	0.78

For peer review only - http://bmjopen.bmj.com/site/about/guidelines.xhtml

Table 3: Percent Agreement and Cohen's Kappa Coefficient Among Nine Facility Readiness Indices

Haiti

	Core simple		Core weighted		Core PCA		Expert simple		Expert weighted		Expert PCA		Maximum simple		Maximum weighted	
	% Agreement	Kappa	% Agreement	Kappa	% Agreement	Kappa	% Agreement	Kappa	% Agreement	Kappa	% Agreement	Kappa	% Agreement	Kappa	% Agreement	Kappa
Core simple																
Core weighted	67.91	0.52														
Core PCA	69.96	0.55	51.48	0.27												
Expert simple	79.85	0.70	65.60	0.48	74.45	0.62										
Expert weighted	74.97	0.62	65.60	0.48	66.37	0.50	77.41	0.66								
Expert PCA	71.89	0.58	53.53	0.30	86.01	0.79	81.77	0.73	67.01	0.51						
Maximum simple	77.02	0.66	62.64	0.44	63.54	0.45	75.87	0.64	68.68	0.53	68.29	0.52				
Maximum weighted	82.54	0.74	65.98	0.49	67.91	0.52	77.79	0.67	73.68	0.61	69.58	0.54	84.47	0.77		
Maximum PCA	68.68	0.53	51.73	0.28	79.46	0.69	75.48	0.63	65.08	0.48	81.90	0.73	76.77	0.65	74.58	0.62

Malawi

	Core simple		Core weighted		Core PCA		Expert simple		Expert weighted		Expert PCA		Maximum simple		Maximum weighted	
	% Agreement	Kappa	% Agreement	Kappa	% Agreement	Kappa	% Agreement	Kappa	% Agreement	Kappa	% Agreement	Kappa	% Agreement	Kappa	% Agreement	Kappa
Core simple																
Core weighted	75.58	0.63														
Core PCA	82.09	0.73	65.40	0.48												
Expert simple	78.04	0.67	65.28	0.48	71.90	0.58										
Expert weighted	71.66	0.57	66.01	0.49	66.38	0.50	74.48	0.62								
Expert PCA	76.69	0.65	62.70	0.44	79.51	0.69	81.10	0.72	70.18	0.55						
Maximum simple	73.13	0.60	59.88	0.40	74.85	0.62	70.67	0.56	63.68	0.45	70.18	0.55				

For peer review only - http://bmjopen.bmj.com/site/about/guidelines.xhtml

Maximum weighted	77.42	0.66	64.91	0.47	77.55	0.66	73.50	0.60	65.03	0.48	70.31	0.55	79.51	0.69		
Maximum PCA	79.02	0.69	63.19	0.45	86.63	0.80	78.40	0.68	66.75	0.50	80.86	0.71	82.58	0.74	80.61	0.71

Tanzania

	Core simple		Core weighted		Core PCA		Expert simple		Expert weighted		Expert PCA		Maximum simple		Maximum weighted	
	% Agreement	Kappa	% Agreement	Kappa	% Agreement	Kappa	% Agreement	Kappa	% Agreement	Kappa	% Agreement	Kappa	% Agreement	Kappa	% Agreement	Kappa
Core simple																
Core weighted	75.19	0.63														
Core PCA	88.28	0.82	66.57	0.50												
Expert simple	72.69	0.59	63.36	0.45	76.80	0.65										
Expert weighted	70.55	0.56	63.36	0.45	73.88	0.61	80.31	0.70								
Expert PCA	73.65	0.60	59.73	0.40	81.56	0.72	89.77	0.85	76.26	0.64						
Maximum simple	71.62	0.57	63.36	0.45	72.75	0.59	79.83	0.70	71.62	0.57	77.57	0.66				
Maximum weighted	77.33	0.66	65.91	0.49	77.51	0.66	75.01	0.63	71.92	0.58	74.30	0.61	84.47	0.77		
Maximum PCA	71.45	0.57	59.55	0.39	76.74	0.65	80.55	0.71	72.93	0.59	81.86	0.73	84.06	0.76	79.00	0.69

Legend	<0	0 — 0.20	0.21 — 0.40	0.41 — 0.60	0.61 — 0.80	0.81 — 1.0
	No agreement/ Worse than chance	Slight	Fair	Moderate	Good	Near perfect

For peer review only - http://bmjopen.bmj.com/site/about/guidelines.xhtml

Table 4: Variability and Distribution of Index Scores for the Nine Facility Readiness Indices

	Haiti			Malawi			Tanzania		
	CoV	Floor Effects	Ceiling Effects	CoV	Floor Effects	Ceiling Effects	CoV	Floor Effects	Ceiling Effects
Core simple	21.20	0.00	0.00	21.70	0.00	1.10	19.11	0.00	0.48
Core weighted	22.67	0.00	0.00	21.45	0.00	1.10	20.83	0.00	0.48
Core PCA	34.61	0.00	0.00	32.09	0.00	1.10	21.96	0.00	0.48
Expert simple	24.30	0.00	0.39	19.64	0.00	1.23	20.14	0.00	3.33
Expert weighted	26.01	0.00	0.39	19.82	0.00	1.23	20.24	0.00	3.33
Expert PCA	35.72	0.00	0.51	18.96	0.00	1.23	23.62	0.00	3.33
Maximum simple	21.12	0.00	0.00	19.33	0.00	0.00	21.31	0.00	0.00
Maximum weighted	24.07	0.00	0.00	18.98	0.00	0.00	21.69	0.00	0.00
Maximum PCA	26.02	0.00	0.00	20.67	0.00	1.10	22.73	0.00	0.36

For peer review only

Table 5: Inclusion of Items in an Index Across a Range of Frequencies for the Facility Readiness Indices

	Haiti			Malawi			Tanzania		
	No./% items <30% frequency	No./% items <40% frequency	No./% items >90% frequency	No./% items <30% frequency	No./% items <40% frequency	No./% items >90% frequency	No./% items <30% frequency	No./% items <40% frequency	No./% items >90% frequency
Core (21 items)	2 (10%)	4 (19%)	5 (24%)	4 (19%)	7 (33%)	6 (29%)	2 (10%)	2 (10%)	8 (38%)
Expert (19 items)	2 (11%)	3 (16%)	4 (21%)	2 (11%)	5 (26%)	7 (37%)	0 (0%)	0 (0%)	7 (37%)
Maximum (38 items)	8 (21%)	10 (26%)	5 (13%)	8 (21%)	12 (32%)	8 (21%)	5 (13%)	9 (24%)	9 (24%)

For peer review only

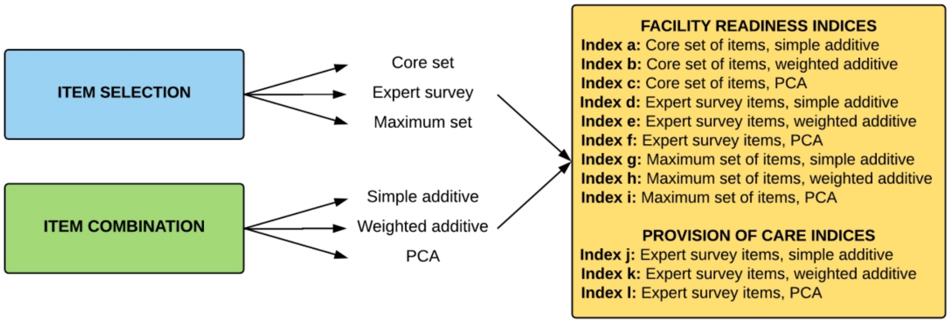
ITEM SELECTION
Core set
Expert survey
Maximum set
ITEM COMBINATION
Simple additive
Weighted additive
PCA
FACILITY READINESS INDICES
Index a: Core set of items, simple additive
Index b: Core set of items, weighted additive
Index c: Core set of items, PCA
Index d: Expert survey items, simple additive
Index e: Expert survey items, weighted additive
Index f: Expert survey items, PCA
Index g: Maximum set of items, simple additive
Index h: Maximum set of items, weighted additive
Index i: Maximum set of items, PCA
PROVISION OF CARE INDICES
Index j: Expert survey items, simple additive
Index k: Expert survey items, weighted additive
Index l: Expert survey items, PCA

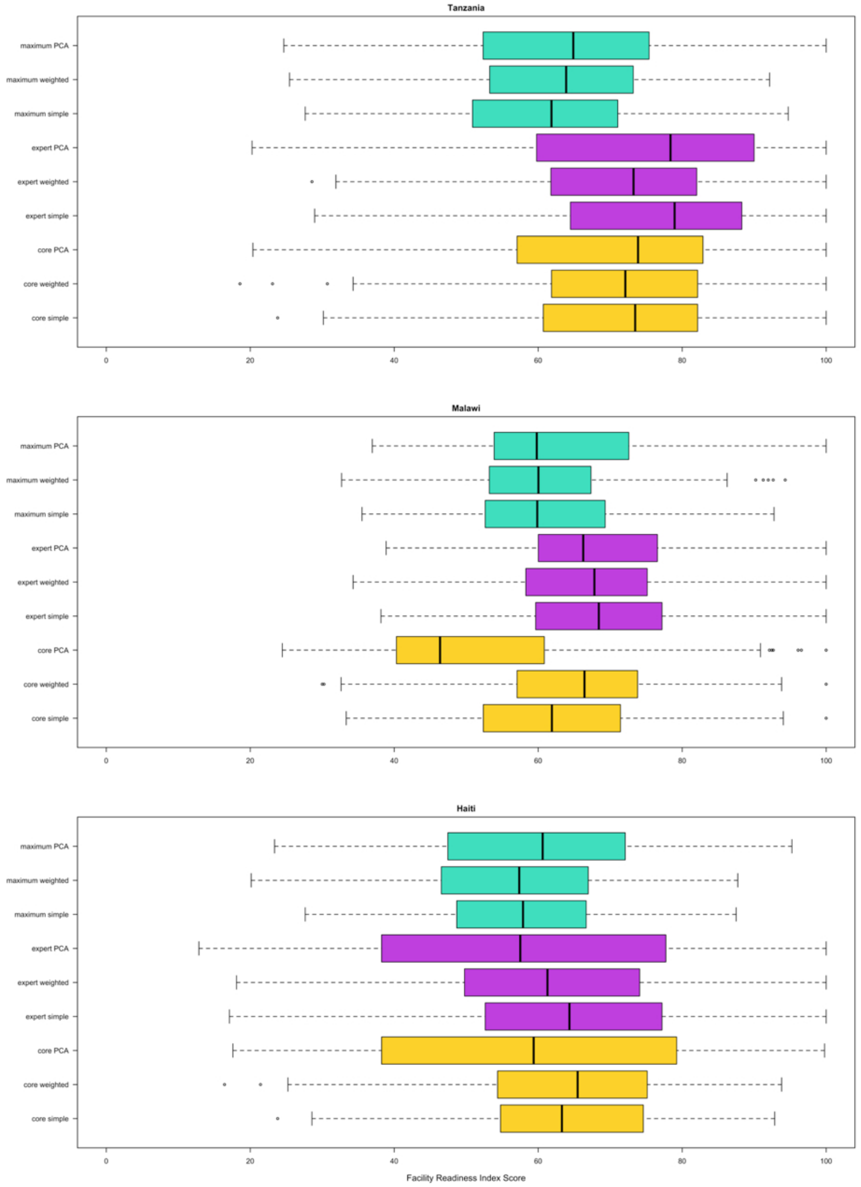
Tanzania
maximum PCA
maximum weighted
maximum simple
expert PCA
expert weighted
expert simple
core PCA
core weighted
core simple
0
20
40
60
80
100
Malawi
Haiti
Facility Readiness Index Score

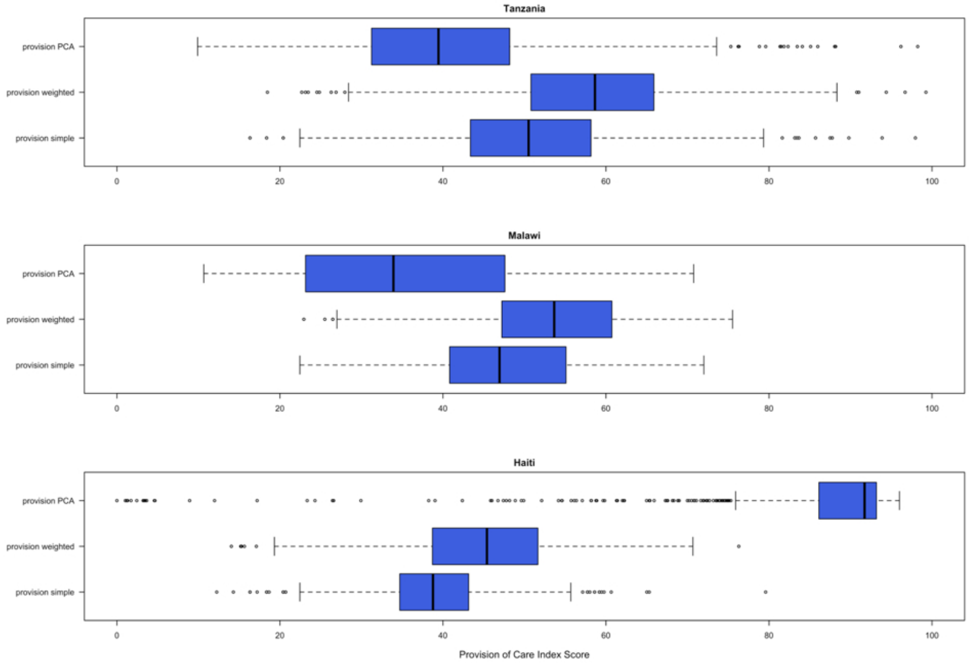
Tanzania
provision PCA
provision weighted
provision simple
0
20
40
60
80
100
Malawi
provision PCA
provision weighted
provision simple
0
20
40
60
80
100
Haiti
provision PCA
provision weighted
provision simple
0
20
40
60
80
100
Provision of Care Index Score

Supplementary Tables

Supplementary Table 1: Summary of Literature Using SPA Data to Create ANC Quality of Care Indices

Author	Year	Country	Type of index and number of items	Item selection	Item combination
Diamond-Smith et al	2016	Kenya, Namibia	Facility readiness: 8 items	DHS tracer indicators of ANC quality	Simple additive
Do et al	2017	Kenya, Namibia	Facility readiness: 14 items<br>Provision of care: 7 items	Donabedian framework and the WHO SARA indicators	Factor analysis
Kruk et al	2017	Kenya, Malawi, Namibia, Rwanda, Senegal, Uganda, Tanzania	Provision of care: 8 items	Clinical guidelines from the WHO	Simple additive
Lee et al	2016	Kenya	Facility readiness and provision of care: 14 items	WHO definition of quality of care	Weighted additive
Sharma et al	2017	Kenya	Facility readiness: 15 items<br>Provision of care: 40 items	Donabedian framework, IOM definition of quality of care, and Focused ANC Model Checklist	Simple additive
Sipsma et al	2012	Rwanda	Provision of care: 8 items	Recommended practices derived from clinical guidelines	Simple additive

For peer review only - http://bmjopen.bmj.com/site/about/guidelines.xhtml

Supplementary Table 2: List of Items Included in the Facility Readiness Indices, by Domain

#	Item Name	Core set	Expert survey	Maximum set
	EQUIPMENT AND SUPPLIES<br>*Availability of…*			
1	Blood pressure apparatus	X	X	X
2	Examination light	X		X
3	Stethoscope	X	X	X
4	Adult weighing scale	X		X
5	Tape measure for fundal height	X		X
6	Examination bed	X	X	X
7	Latex gloves		X	X
8	Single use syringes	X	X	X
9	Soap and water OR alcohol-based hand rub		X	X
10	Disinfectant		X	X
11	Appropriate storage of sharps waste (sharps box)			X
12	Appropriate storage of infectious waste (pedal bin with lid and liner)			X
13	Safe final disposal of sharps (incineration)			X
14	Safe final disposal of infectious wastes (incineration)			X
15	Medical masks			X
16	Gowns			X
17	Eye protection			X
	DIAGNOSTICS<br>*Availability on-site to test for…*			
18	Hemoglobin	X	X	X
19	Urine dipstick- protein	X	X	X
20	Urine dipstick- glucose	X	X	X
21	Grouping and Rhesus factor	X		X
22	Syphilis RDT / RPR	X	X	X
23	HIV testing / RDT	X	X	X
	MEDICINES AND COMMODITIES<br>*Availability on-site of…*			
24	Iron tablets	X	X	X
25	Folic acid tablets	X	X	X
26	Tetanus toxoid vaccine	X	X	X
27	De-worming drugs (mebendazole / albendazole)	X		X
	BASIC AMENITIES<br>*Availability of…*			

For peer review only - http://bmjopen.bmj.com/site/about/guidelines.xhtml

#	Item Name	Core set	Expert survey	Maximum set
28	Power			X
29	Improved water source		X	X
30	Room with auditory and visual privacy	X	X	X
31	Sanitation facilities		X	X
32	Communication equipment			X
33	Computer with email/internet access			X
34	Emergency transportation			X
	HUMAN RESOURCES<br>*Availability of…*			
35	Guidelines for ANC available at the facility	X		X
36	Proportion of health facility staff providing ANC services trained in ANC in the last two years	X	X	X
37	Proportion of health facility staff providing ANC services who received supervision in the last six months	X		X
38	Proportion of health facility staff providing ANC services who report availability of opportunities for promotion in their current job			X

For peer review only - http://bmjopen.bmj.com/site/about/guidelines.xhtml

Supplementary Table 3: Expert Survey Results (mean, sd, min, max, n)

**Item Name**	**Mean**	**Standard Deviation**	**Min.**	**Max.**	**N**
EQUIPMENT AND SUPPLIES *Availability of…*					
Blood pressure apparatus	3.933	0.258	3	4	15
Examination light	2.600	0.828	1	4	15
Fetal stethoscope	3.733	0.594	2	4	15
Stethoscope	3.467	0.834	2	4	15
Adult weighing scale	3.000	0.845	2	4	15
Thermometer	3.533	0.834	1	4	15
Tape measure for fundal height	3.333	0.724	2	4	15
Height board	2.692	0.947	1	4	13
Speculum	3.429	0.646	2	4	14
Examination bed	3.429	0.938	1	4	14
Latex gloves	3.600	0.632	2	4	15
Single use syringes	3.643	0.745	2	4	14
Soap and water OR alcohol-based hand rub	3.733	0.704	2	4	15
Disinfectant	3.533	0.640	2	4	15
Appropriate storage of sharps waste (sharps box)	3.400	0.910	1	4	15
Appropriate storage of infectious waste (pedal bin with lid and liner)	3.333	0.976	1	4	15
Safe final disposal of sharps (incineration)	3.200	0.941	1	4	15
Safe final disposal of infectious wastes (incineration)	3.133	0.990	1	4	15
Medical masks	2.200	0.775	1	4	15
Gowns	2.000	0.845	1	4	15
Eye protection	2.067	0.884	1	4	15
Gum boots	1.667	0.724	1	3	15
DIAGNOSTICS *Availability on-site to test for…*					
Hemoglobin	3.800	0.414	3	4	15
Urine dipstick- protein	3.733	0.458	3	4	15
Urine dipstick- glucose	3.467	0.743	2	4	15
Grouping and Rhesus factor	3.357	0.745	2	4	14
Syphilis RDT / RPR	3.667	0.488	3	4	15
HIV testing / RDT	3.667	0.488	3	4	15

For peer review only - http://bmjopen.bmj.com/site/about/guidelines.xhtml

Item Name	Mean	Standard Deviation	Min.	Max.	N
MEDICINES AND COMMODITIES *Availability on-site of…*					
Iron tablets	3.800	0.414	3	4	15
Folic acid tablets	3.667	0.617	2	4	15
Tetanus toxoid vaccine	3.800	0.414	3	4	15
Intermittent Preventative Treatment (IPT) drug	3.667	0.488	3	4	15
Insecticide treated nets (ITNs) or vouchers	3.267	0.799	2	4	15
De-worming drugs (mebendazole / albendazole)	3.000	0.784	2	4	14
BASIC AMENITIES *Availability of…*					
Power	3.400	0.737	2	4	15
Improved water source	3.667	0.617	2	4	15
Room with auditory and visual privacy	3.533	0.640	2	4	15
Sanitation facilities	3.600	0.507	3	4	15
Communication equipment	3.267	0.884	1	4	15
Computer with email/internet access	2.267	1.033	1	4	15
Emergency transportation	3.400	0.986	1	4	15
HUMAN RESOURCES *Availability of…*					
Guidelines for ANC available at the facility	3.267	0.884	1	4	15
Proportion of health facility staff providing ANC services trained in ANC in the last two years	3.400	0.737	2	4	15
Proportion of health facility staff providing ANC services who received supervision in the last six months	3.267	0.799	2	4	15
Proportion of health facility staff providing ANC services who report availability of opportunities for promotion in their current job	2.500	0.760	2	4	14
HISTORY-TAKING *During ANC visit the provider asks about…*					
Personal history: client age	3.533	0.640	2	4	15
Personal history: medications client is taking	3.733	0.458	3	4	15
Personal history: date last menstrual period began	3.933	0.258	3	4	15

For peer review only - http://bmjopen.bmj.com/site/about/guidelines.xhtml

Item Name	Mean	Standard Deviation	Min.	Max.	N
Personal history: any prior pregnancy	3.867	0.352	3	4	15
Family history	3.200	0.862	2	4	15
Social history	3.067	0.704	2	4	15
Past medical history for prior pregnancies: still birth	3.867	0.352	3	4	15
Past medical history for prior pregnancies: infant died in the first week of life	3.600	0.632	2	4	15
Past medical history for prior pregnancies: heavy bleeding during or after delivery	3.733	0.458	3	4	15
Past medical history for prior pregnancies: previous assisted delivery	3.733	0.458	3	4	15
Past medical history for prior pregnancies: previous spontaneous abortion	3.467	0.834	2	4	15
Past medical history for prior pregnancies: multiple pregnancies	3.600	0.737	2	4	15
Past medical history for prior pregnancies: prolonged labor	3.600	0.632	2	4	15
Past medical history for prior pregnancies: pregnancy-induced hypertension	4.000	0.000	4	4	15
Past medical history for prior pregnancies: pregnancy related convulsions	4.000	0.000	4	4	15
Past medical history for prior pregnancies: high fever or infection during prior pregnancy	3.533	0.640	2	4	15
History of complaints in current pregnancy: vaginal bleeding	4.000	0.000	4	4	15
History of complaints in current pregnancy: fever	3.867	0.352	3	4	15
History of complaints in current pregnancy: headache or blurred vision	4.000	0.000	4	4	15
History of complaints in current pregnancy: swollen face or hands or extremities	4.000	0.000	4	4	15
History of complaints in current pregnancy: tiredness or breathlessness	3.667	0.816	1	4	15
History of complaints in current pregnancy: fetal movement (loss of, excessive, normal)	3.933	0.258	3	4	15

For peer review only - http://bmjopen.bmj.com/site/about/guidelines.xhtml

Item Name	Mean	Standard Deviation	Min.	Max.	N
History of complaints in current pregnancy: cough or difficulty breathing for three weeks or longer	3.733	0.458	3	4	15
History of complaints in current pregnancy: amniotic leakage	3.786	0.579	2	4	14
EXAMINATION *During ANC visit the provider examines the client for…*					
Head to toe (whole body)	2.600	1.121	1	4	15
Pallor	3.400	0.632	2	4	15
Oedema	3.733	0.458	3	4	15
Breast	2.533	0.990	1	4	15
Lungs and heart	2.600	1.056	1	4	15
OBSERVATION AND CLINICAL INVESTIGATION *During ANC visit the provider assesses the client's…*					
Blood pressure	4.000	0.000	4	4	15
Weight	3.133	0.834	2	4	15
Temperature	3.333	0.900	1	4	15
Pulse	3.357	0.929	1	4	14
OBSTETRIC COMPLICATIONS *During ANC visit the provider assesses the client for potential obstetric complications by…*					
Palpating the client's abdomen for fundal height	3.667	0.617	2	4	15
Palpating the client's abdomen for fetal presentation	3.600	0.632	2	4	15
Listening to the client's abdomen for fetal heartbeat	3.800	0.561	2	4	15
Conducting an ultrasound/ referring client for ultrasound/ look at recent ultrasound report	2.600	0.986	1	4	15
PELVIC EXAMINATION *During ANC visit the provider conducts a pelvic exam with…*					
Soft tissue assessment/ Vaginal examination	2.154	1.214	1	4	13
Bony pelvic assessment	2.571	1.342	1	4	14
LABORATORY INVESTIGATIONS *During ANC visit the provider performs or refers the client to test for…*					
Hemoglobin	3.733	0.458	3	4	15
Grouping and rhesus factor	3.357	0.633	2	4	14
Syphilis / RPR	3.667	0.488	3	4	15
HIV	3.867	0.352	3	4	15

Item Name	Mean	Standard Deviation	Min.	Max.	N
Urine – protein	3.667	0.617	2	4	15
Urine – glucose	3.400	0.828	2	4	15
Urine – acetone	2.500	1.087	1	4	12
DRUG ADMINISTRATION AND IMMUNIZATION *During ANC visit the provider administers or prescribes…*					
Iron and/or folic acid	3.800	0.414	3	4	15
Antimalarials	3.429	0.756	2	4	14
Tetanus toxoid	3.600	0.737	2	4	15
CLIENT EDUCATION AND COUNSELLING *During ANC visit the provider counsels the client on…*					
Process of pregnancy and its complications	3.733	0.594	2	4	15
Diet and nutrition	3.600	0.632	2	4	15
Rest and exercise in pregnancy	2.933	0.704	2	4	15
Personal hygiene	2.867	0.990	1	4	15
Danger signs in pregnancy	3.933	0.258	3	4	15
Use of drugs in pregnancy	3.400	0.828	2	4	15
Effects of STI/HIV/AIDS	3.467	0.834	2	4	15
Voluntary counselling and testing for HIV	3.667	0.617	2	4	15
Care of breasts	2.867	0.743	2	4	15
Breastfeeding	3.867	0.352	3	4	15
Symptoms/signs of labor	3.867	0.516	2	4	15
Plans of delivery (emergency preparedness, place of delivery, transportation, financial arrangements)	3.733	0.458	3	4	15
Plans for postpartum care	3.400	0.632	2	4	15
Family planning	3.533	0.640	2	4	15
Harmful habits (e.g. smoking, drug abuse, alcoholism)	3.533	0.640	2	4	15
Schedule of return visit	3.867	0.352	3	4	15
CLIENT EXPERIENCE *During ANC visit…*					
Client is able to discuss problems or concerns with provider	3.867	0.352	3	4	15
Client satisfied with the amount of explanation received about the problem or treatment	3.600	0.632	2	4	15

For peer review only - http://bmjopen.bmj.com/site/about/guidelines.xhtml

Item Name	Mean	Standard Deviation	Min.	Max.	N
Client satisfied with how the staff treated them	3.667	0.617	2	4	15
Privacy from having others see the consultation	3.533	0.640	2	4	15
Privacy from having others hear the consultation	3.533	0.640	2	4	15
Client satisfied with the wait time	3.133	0.834	2	4	15
Client satisfied with the number of days services are available at the facility	3.000	0.756	2	4	15
Client satisfied with the hours of service at the facility	3.067	0.704	2	4	15
Client satisfied with the cost for services or treatments	3.333	0.617	2	4	15
Client satisfied with the availability of medicines at the facility	3.467	0.516	3	4	15
Client satisfied with the cleanliness of the facility	3.533	0.516	3	4	15

For peer review only

For peer review only - http://bmjopen.bmj.com/site/about/guidelines.xhtml

Supplementary Table 4: List of Items Included in the Provision of Care Indices, by Domain

#	Item Name	Corresponding Readiness Domain
	HISTORY-TAKING AND CLIENT EDUCATION/COUNSELLING *During ANC visit the provider asks about/counsels the client on…*	
1	Personal history: client age	Human Resources
2	Personal history: medications client is taking	Human Resources
3	Personal history: date last menstrual period began	Human Resources
4	Personal history: any prior pregnancy	Human Resources
5	Past medical history for prior pregnancies: still birth	Human Resources
6	Past medical history for prior pregnancies: infant died in the first week of life	Human Resources
7	Past medical history for prior pregnancies: heavy bleeding during or after delivery	Human Resources
8	Past medical history for prior pregnancies: previous assisted delivery	Human Resources
9	Past medical history for prior pregnancies: previous spontaneous abortion	Human Resources
10	Past medical history for prior pregnancies: multiple pregnancies	Human Resources
11	Past medical history for prior pregnancies: prolonged labor	Human Resources
12	Past medical history for prior pregnancies: pregnancy-induced hypertension	Human Resources
13	Past medical history for prior pregnancies: pregnancy related convulsions	Human Resources
14	Past medical history for prior pregnancies: high fever or infection during prior pregnancy	Human Resources
15	History of complaints in current pregnancy: vaginal bleeding	Human Resources
16	History of complaints in current pregnancy: fever	Human Resources
17	History of complaints in current pregnancy: headache or blurred vision	Human Resources
18	History of complaints in current pregnancy: swollen face or hands or extremities	Human Resources
19	History of complaints in current pregnancy: tiredness or breathlessness	Human Resources
20	History of complaints in current pregnancy: fetal movement (loss of, excessive, normal)	Human Resources
21	History of complaints in current pregnancy: cough or difficulty breathing for three weeks or longer	Human Resources
22	Client education/counselling: Process of pregnancy and its complications	Human Resources
23	Client education/counselling: Diet and nutrition	Human Resources
24	Client education/counselling: Danger signs in pregnancy	Human Resources
25	Client education/counselling: Voluntary counselling and testing for HIV	Human Resources
26	Client education/counselling: Breastfeeding	Human Resources
27	Client education/counselling: Plans of delivery (emergency preparedness, place of delivery, transportation, financial arrangements)	Human Resources
	EXAMINATION *During ANC visit the provider examines the client for…*	
28	Oedema	Human Resources

For peer review only - http://bmjopen.bmj.com/site/about/guidelines.xhtml

#	Item Name	Corresponding Readiness Domain
29	Blood pressure	Human Resources, Equipment
30	Weight	Human Resources, Equipment
31	Palpating the client's abdomen for fundal height	Human Resources, Equipment
	DIAGNOSTICS<br>*During ANC visit the provider performs or refers the client to test for…*	
32	Hemoglobin	Human Resources, Diagnostics
33	Grouping and rhesus factor	Human Resources, Diagnostics
34	Syphilis / RPR	Human Resources, Diagnostics
35	HIV	Human Resources, Diagnostics
36	Urine – protein, glucose	Human Resources, Diagnostics
	PREVENTATIVE TREATMENT<br>*During ANC visit the provider administers or prescribes…*	
37	Iron and/or folic acid	Human Resources, Medicines
38	Tetanus toxoid	Human Resources, Medicines
	CLIENT EXPERIENCE<br>*During ANC visit…*	
39	Client is able to discuss problems or concerns with provider	Human Resources
40	Client satisfied with the amount of explanation received about the problem or treatment	Human Resources
41	Client satisfied with how the staff treated them	Human Resources
42	Privacy from having others see the consultation	Human Resources, Basic Amenities
43	Privacy from having others hear the consultation	Human Resources, Basic Amenities
44	Client satisfied with the wait time	Human Resources
45	Client satisfied with the number of days services are available at the facility	Human Resources
46	Client satisfied with the hours of service at the facility	Human Resources
47	Client satisfied with the cost for services or treatments	Human Resources
48	Client satisfied with the availability of medicines at the facility	Human Resources, Medicines
49	Client satisfied with the cleanliness of the facility	Human Resources

For peer review only - http://bmjopen.bmj.com/site/about/guidelines.xhtml

For peer review only

For peer review only - http://bmjopen.bmj.com/site/about/guidelines.xhtml

Supplementary Table 5: Summary Statistics for Facility Readiness Calculated as a Client-Level Dataset

	core simple	core weighted	core PCA	expert simple	expert weighted	expert PCA	maximum simple	maximum weighted	maximum PCA
**Haiti (N = 779)**									
mean	64.9	64.7	62.3	65.8	63.1	61.3	58.9	57.8	61.8
SE.mean	0.5	0.5	0.8	0.6	0.6	0.8	0.4	0.5	0.6
median	65.5	65.5	64.8	65.8	63.6	63.4	59.5	58.4	63.5
min	23.8	16.4	17.6	17.1	18.1	12.9	27.6	20.1	23.4
max	92.9	93.8	99.8	100.0	100.0	100.0	87.5	87.7	95.2
range	69.0	77.4	82.2	82.9	81.9	87.1	59.9	67.6	71.9
IQR	19.8	20.5	40.8	25.6	23.2	38.1	18.3	18.9	25.6
**Malawi (N = 815)**									
mean	63.4	65.9	52.7	69.3	68.3	70.1	61.7	61.5	63.7
SE.mean	0.5	0.5	0.6	0.5	0.5	0.5	0.4	0.4	0.5
median	62.7	66.7	47.3	68.4	67.8	66.5	60.5	60.0	60.9
min	33.3	30.0	24.5	38.2	34.3	38.9	35.5	32.7	37.0
max	100.0	100.0	100.0	100.0	100.0	100.0	92.8	94.3	100.0
range	66.7	70.0	75.5	61.8	65.7	61.1	57.2	61.6	63.0
IQR	20.6	18.7	20.1	19.3	17.4	15.6	17.1	16.2	18.9
**Tanzania (N = 1,681)**									
mean	72.3	71.9	71.2	77.1	73.0	76.0	62.2	64.0	65.4
SE.mean	0.3	0.4	0.4	0.4	0.4	0.4	0.3	0.3	0.4
median	74.6	73.8	75.0	80.3	74.3	80.2	63.7	65.6	66.9
min	23.8	18.6	20.4	28.9	28.6	20.3	27.6	25.5	24.7
max	100.0	100.0	100.0	100.0	100.0	100.0	94.7	92.1	100.0
range	76.2	81.4	79.6	71.1	71.4	79.7	67.1	66.7	75.3
IQR	21.1	20.7	26.1	24.6	20.2	30.5	20.1	19.7	22.8

For peer review only

BMJ Open

Development of Summary Indices of Antenatal Care Service Quality in Haiti, Malawi, and Tanzania

Journal:	*BMJ Open*
Manuscript ID	bmjopen-2019-032558.R1
Article Type:	Research
Date Submitted by the Author:	20-Sep-2019

Complete List of Authors:	Sheffel, Ashley; Johns Hopkins University Bloomberg School of Public Health, Department of International Health<br>Zeger, Scott; Johns Hopkins University Bloomberg School of Public Health, Department of Biostatistics; Johns Hopkins University Bloomberg School of Public Health, Department of International Health<br>Heidkamp, Rebecca; Johns Hopkins University Bloomberg School of Public Health, International Health<br>Munos, Melinda; Johns Hopkins University Bloomberg School of Public Health, Department of International Health

**Primary Subject Heading**:	Public health
Secondary Subject Heading:	Global health, Health services research
Keywords:	Maternal medicine < OBSTETRICS, International health services < HEALTH SERVICES ADMINISTRATION & MANAGEMENT, Quality in health care < HEALTH SERVICES ADMINISTRATION & MANAGEMENT, PUBLIC HEALTH, STATISTICS & RESEARCH METHODS

SCHOLARONE™
Manuscripts

Title: Development of Summary Indices of Antenatal Care Service Quality in Haiti, Malawi, and Tanzania

Authors: Ashley Sheffel[1], Scott Zeger[1,2], Rebecca Heidkamp[1], Melinda Munos[1]

Affiliations:

[1]Department of International Health, Johns Hopkins Bloomberg School of Public Health, Baltimore, MD, USA

[2]Department of Biostatistics, Johns Hopkins Bloomberg School of Public Health, Baltimore, MD, USA

Author contributions: AS, MM, RH, and SZ contributed to conceptualizing the paper and analysis; AS prepared the manuscript; MM, RH, and SZ critically reviewed and revised the manuscript.

Corresponding author:

Ashley Sheffel

Johns Hopkins Bloomberg School of Public Health

615 N Wolfe St. Room E8009

Baltimore, MD 21205 USA

asheffel@jhu.edu

Keywords: Maternal medicine, International health services, Quality in health care, Public health, Statistics & research methods

Word count: 5,421

Reference count: 54

Table/Figure count: 5 tables, 3 figures

Funding: This work was supported by the Improving Measurement and Program Design grant (OPP1172551) from the Bill & Melinda Gates Foundation.

For peer review only - http://bmjopen.bmj.com/site/about/guidelines.xhtml

Abbreviations:

ANC – Antenatal Care

CoV – Coefficient of Variation

DHS – Demographic and Health Survey

FANC – Focused Antenatal Care

IQR – Inter-Quartile Range

LMIC – Low-Income and Middle- Income Countries

PCA – Principal Components Analysis

SPA – Service Provision Assessment

USAID – US Agency for International Development

For peer review only

For peer review only - http://bmjopen.bmj.com/site/about/guidelines.xhtml

ABSTRACT

Introduction: Measuring quality of care in low-income and middle-income countries is complicated by the lack of a standard, universally-accepted definition for "quality" for any particular service, as well as limited guidance on which indicators to include in measures of quality of care, and how to incorporate those indicators into summary indices. The aim of this paper is to develop, characterize, and compare a set of antenatal care (ANC) indices for facility readiness and provision of care.

Methods: We created nine indices for facility readiness using three methods for selecting items and three methods for combining items. In addition, we created three indices for provision of care using one method for selecting items and three methods for combining items. For each index, we calculated descriptive statistics, categorized the continuous index scores using tercile cut points to assess comparability of facility classification, and examined the variability and distribution of scores.

Results: Our results showed that within a country the indices were quite similar in terms of mean index score, facility classification, coefficient of variation, floor and ceiling effects, and the inclusion of items in an index with a range of variability. Notably, the indices created using PCA to combine the items were the most different from the other indices. In addition, the index created by taking a weighted average of a core set of items had lower agreement with the other indices when looking at facility classification.

For peer review only - http://bmjopen.bmj.com/site/about/guidelines.xhtml

Conclusions: As improving quality of care becomes integral to global efforts to produce better health outcomes, demand for guidance on creating standardized measures of service quality will grow. This study provides health systems researchers with a comparison of methodologies commonly used to create summary indices of ANC service quality and it highlights the similarities and differences between methods.

For peer review only

For peer review only - http://bmjopen.bmj.com/site/about/guidelines.xhtml

STRENGTHS AND LIMITATIONS OF THIS STUDY

- To our knowledge, this is the first study that uses multiple methodologies to develop and compare indices for ANC facility readiness and provision of care.
- Our approaches to item selection and combination were based on the most commonly used approaches in the literature and we included those most likely to be useful to LMICs.
- The analysis was conducted using data from multiple countries which strengthens our ability to generalize the findings to other similar contexts.
- The SPA survey, while quite comprehensive, does not capture every aspect of quality of care that may be important to include in quality of care metrics.

  This analysis does not provide a formal validation of any one ANC quality of care index.

For peer review only

Introduction

Reducing maternal morbidity and mortality has been a global priority over the last several decades. While progress has been made in both improving coverage and reducing mortality, there remains an unacceptably high number of deaths of women globally [1]. In many countries, considerable increases in the coverage of health services have not translated into sufficient reductions in mortality [2]. This finding suggests that quality of care may play a critical role in producing better health outcomes. Reducing maternal mortality will require not only increasing the coverage of health interventions, but also ensuring that those interventions are delivered with a high level of quality [3 4]. These interventions include not only intrapartum and postpartum care, but also antenatal care (ANC), which is important to maintaining a healthy pregnancy [5-7], and promotes safe delivery, postnatal attendance, and is positively associated with an increase in facility-based deliveries [8-10].

Low-income and middle-income countries (LMICs) are increasingly shifting focus towards making improvements in the quality of health services delivered, which requires measuring quality of care and monitoring progress. However, for LMICs to systematically and comprehensively measure quality of care, they require a definition of quality of care and clear, specific indicators to operationalize the definition. Many quality of care frameworks exist, most of which build from Donabedian's 1988 framework characterizing quality at three levels: structure, commonly called facility readiness (the setting in which care occurs including material resources, human resources, and organizational structure), process (commonly called provision of care — the quality of medical advice delivered by providers to clients, as well as interpersonal relationships between the provider and the client), and outcome (the effects of care on the health status and behaviors of patients, as well as improvements in patient knowledge and the degree of

For peer review only - http://bmjopen.bmj.com/site/about/guidelines.xhtml

satisfaction with care) [11 12]. While there may be growing consensus on the core components of a framework for describing quality of care, measuring quality of care is complicated by lack of a standard, universally accepted definition for "quality" for any one particular service [13-15]. In addition, there is limited guidance on which indicators to include in measures of quality of care and how to incorporate those indicators into summary indices.

Studies on quality of care in LMICs largely rely on data from health facility surveys that collect data on facility readiness and provision of care. These studies often use summary indices or composite scores to provide an overall description of service quality[16]. Summary scores are useful to LMIC governments in that they simplify complex data and enable comparison of performance within facilities, across administrative units, and over time. However, there is little consistency between studies in terms of the items included in summary metrics of quality of care, or in how the metrics are created. A scoping review published in 2018 identified numerous studies which utilized health facility survey data to assess maternal and newborn quality of care in LMICs and found that studies used various approaches to create quality of care metrics [17]. ***Supplementary Table 1*** illustrates this with an excerpt of studies that used data from the Service Provision Assessment (SPA) to create quality of care metrics for ANC specifically, each using a different approach. Item selection was guided by different sources including the Donabedian quality of care framework (for example [18 19]), clinical guidelines (for example [20 21 22 23]), and various definitions of quality of care (for example [24 25]). The number of items that were included in an index varied from a minimum of seven to a maximum of forty, and various methods were used to combine items into indices. These methods include a simple and weighted additive approaches (for example [24 26-30]) as well as principal components analysis (PCA) (for example [19 31-33]). While many of these studies had some overlap in the items selected for inclusion in their

For peer review only - http://bmjopen.bmj.com/site/about/guidelines.xhtml

ANC quality of care index, the quality of care indices varied greatly in the items selected for inclusion in the indices and in the methodology utilized for combining items into a summary measure.

As quality of care indices are increasingly used by countries, global agencies, and researchers to quantify the quality of health services and estimate effective coverage, there is a need to standardize the methodology for creating these measures. To date, few studies have compared the methodologies used to create indices of ANC service quality. The objective of this paper is to develop, characterize, and compare a set of facility readiness and provision of care indices for ANC.

METHODS

Overall approach

Using data from an expert survey, we created nine facility readiness indices using three methods for selecting items (a "core set" of items, "expert survey" set of items, and "maximum" set of items) and three methods for combining items (simple additive, weighted additive, and PCA) (***Figure 1***). We created three indices for provision of care using one method for selecting items ("expert survey" set of items) and three methods for combining items (simple additive, weighted additive, and PCA) (***Figure 1***). Data for these indices comes from the Service Provision assessment (SPA). We then compared the indices and examined the variability and distribution of the index scores.

Data Source

The Service Provision Assessment (SPA) is a health facility assessment used in LMICs to generate nationally representative data on health service delivery [34]. SPA data are publicly available through the Demographic and Health Survey (DHS) program. The SPA includes a

For peer review only - http://bmjopen.bmj.com/site/about/guidelines.xhtml

standard set of survey instruments: a facility inventory questionnaire, health worker interviews, observation of ANC consultations, and exit interviews with ANC clients.

We examined all SPA surveys for inclusion in the analysis (total of 16). We included all SPA surveys which utilized the DHS-VI or DHS-VII questionnaire (four surveys excluded), that included observations of ANC consultations (three surveys excluded), were conducted in the last five years (between 2013 and 2018) (two surveys excluded), and for which recode data files were available (four surveys excluded). The included surveys are from Haiti (2013), Malawi (2013/14), and Tanzania (2014/15). The SPA final country reports contain comprehensive information on the survey methodology and questionnaires [35-37]. Briefly described, the survey in Tanzania was a nationally representative sample of health facilities. Health facilities were selected using stratified systematic probability sampling with stratification by region and facility type (with oversampling of some facility types such as hospitals). In Haiti and Malawi, the survey was a census of all health facilities in the country. In all three countries, all surveyed facilities completed the facility inventory module. In addition, within each facility, up to eight health workers were interviewed including all health workers whose consultations were observed and those who provided information for any section of the facility questionnaire. ANC clients were selected for observation using systematic sampling, based on the number of clients present at each service site on the day of the visit. At facilities where the number of ANC clients expected on the day of the survey could not be predetermined, the sample was opportunistic since clients were selected as they arrived. Observation was completed for a minimum of five clients per service provider, with a maximum of 15 observations in any given facility for each service. Client exit interviews were conducted with every client whose visit was observed.

Item selection

For peer review only - http://bmjopen.bmj.com/site/about/guidelines.xhtml

To identify elements of facility readiness and the provision of ANC considered by experts to be the most important to ANC service quality, we reviewed the SARA indicators, WHO Focused Antenatal Care (FANC) guidelines, and WHO recommendations on antenatal care for a positive pregnancy experience, as well as the SPA questionnaire. This process identified a total of 121 items organized according to the dimensions of quality of care proposed by the WHO Quality of Care Framework for maternal and newborn health: essential physical resources (41 items), competent and motivated human resources (four items), provision of care (65 items), and experience of care (11 items) [38].

We then conducted a survey of 50 maternal health experts who had experience working in LMICs. Respondents were asked to rate each item based on overall importance. Importance ratings ranged from one (item was unimportant) to four (item was essential). Experts also provided a list of items they felt were important for delivering high-quality ANC services, but were missing from the survey instrument. Fifteen maternal health experts completed the survey, with respondents representing academic institutions, donor agencies, United Nations agencies, global health implementing organizations, and Ministries of Health in LMICs.

Items that were not collected across SPA surveys in all three countries and items that are not required for a first ANC visit were excluded from all indices. For each readiness item, a binary score was created based on whether the criteria for availability were met on the day of the survey (1=available, 0=not available). For human resources items, each item is a proportion ranging from 0 to 1. For provision of care items, a binary score was created based on whether or not the activity was conducted during the ANC visit (1=conducted, 0=not conducted) or whether or not the client had a problem with the item during the ANC visit (1=no problem, 0=major or

For peer review only - http://bmjopen.bmj.com/site/about/guidelines.xhtml

minor problem). The list of items included in each readiness and provision of care index can be found in ***Supplementary Table 2 and Supplementary Table 3***, respectively.

Three methods were used to select items to include in the facility readiness indices. The first method identified the **core set** of readiness items required to deliver ANC services. This set of items was identified by reviewing the provision of care items required for an ANC visit based on the WHO FANC guidelines and the WHO recommendations on antenatal care for a positive pregnancy experience, and by determining the human resources, equipment and supplies, medicines, and diagnostics required to deliver each specific item. In creating the core set of items for facility readiness, we mapped each provision of care item to the facility readiness items required to deliver the specific service component. We found that of the 49 provision of care items, 36 items required only human resources and 13 items required human resources plus equipment, diagnostics, medicines, or basic amenities. The core index did not include standard precautions for infection prevention items because these are not explicitly required for any one provision of care item. A total of 21 items were selected for the core set readiness index.

The second method utilized results from the **expert survey** to identify the set of readiness items maternal health experts identified as essential to deliver ANC services. Mean ratings were calculated for each readiness item (see ***Supplementary Table 4*** for results). Items rated by the expert group with a mean importance of >3.4 out of 4 were selected for the expert survey index. The threshold for inclusion was determined by examining the distribution of scores and identifying a natural break point which separated the top-rated items from the rest. In addition, items were selected so that at least one item per domain (human resources, equipment and supplies, diagnostics, medicines, basic amenities) was included in the index. A total of 19 items were selected, representing 42% of the total items for the expert survey readiness index.

For peer review only - http://bmjopen.bmj.com/site/about/guidelines.xhtml

The third method identified the **maximum set** of readiness items used to deliver ANC services. This index includes all items identified in the SPA related to ANC readiness across the following domains: human resources, equipment and supplies, medicines, diagnostics, and basic amenities. Out of the 45 facility readiness items identified for inclusion in the expert survey, a total of 38 items were selected for the maximum set readiness index. Seven items from the expert survey were not included in the maximum set of readiness items as data was not collected in the SPA on these items.

We utilized a single method to select items to include in the provision of care index. The method utilized results from the **expert survey** to select the set of provision of care items maternal health experts identified as essential to deliver ANC services. We chose this method because maternal health experts were the best source for determining which processes are essential to high quality ANC consultations. In addition, the experts selected most items as very important or essential, and therefore it was not appropriate to define a core and maximum set of items. Mean ratings were calculated for each provision of care item (see ***Supplementary Table 4*** for results). Items rated by the expert group with a mean importance of ≥3.0 out of 4 were selected for the provision of care index. The threshold for inclusion was decided by examining the distribution of scores which showed a bimodal distribution; then, selecting all items in the first mode. The expert survey respondents mentioned a number of provision of care items, largely related to experience of care, that they felt were essential for high quality care, but were missing from our survey and from SPA datasets. A total of 49 items were selected representing 64% of the total items for the provision of care index.

Item Combination

For peer review only - http://bmjopen.bmj.com/site/about/guidelines.xhtml

Three methods were used to combine items to create the facility readiness and provision of care indices — simple additive, weighted additive, and principal components analysis (PCA).

Simple additive: The simple additive index score was calculated by taking a sum of the items available divided by the total number of items in the index. We transformed the index into a score out of 100 by dividing by the number of items and multiplying by 100.The simple additive index weighted all items in the index equally.

Weighted additive: The weighted additive index was also calculated as a sum of items, but instead of assuming equal weights for all items, the weighted additive index accounted for the number of items within each domain. Readiness items were first grouped into five domains: human resources, equipment and supplies, medicines, diagnostics, and basic amenities. For the provision of care index, items were initially grouped into five domains: history and counselling, examination, diagnostics, preventative treatment, and client experience. We then computed a domain score by adding the items within each domain and dividing by the total number of items in the domain. Finally, we transformed the index into a score out of 100 by averaging the domain scores and multiplying by 100.

Principal components analysis (PCA): To create the PCA index score, we conducted an unrotated, unweighted PCA using a correlation matrix and used the factor loadings from the first principal component to create the index score. We rescaled the score obtained from the first component of the PCA to a range of zero to 100 for comparability with other indices.

Analysis

We limited the readiness analysis to facilities offering ANC services and with at least one first visit ANC client observation. In order to standardize expected clinical actions, we limited

For peer review only - http://bmjopen.bmj.com/site/about/guidelines.xhtml
For peer review only

the provision of care analysis to women attending the health facility for their first ANC visit. Finally, we excluded cases with incomplete data for any variables of interest.

We calculated descriptive statistics on each of the indices including mean, median, minimum, maximum, and range. The elements of the complex survey design (weights and clustering of observation) were not incorporated into the analysis as the goal of this analysis was not to make inferences about the entire population from which the sample of health facilities was drawn. In addition, for the indices created using PCA to combine items, we calculated factor loadings, the eigenvalue, and the percent of total variance explained by the first component. Since the PCA method was used to create weights for a composite variable rather than estimating a latent variable we did not report out the number of components with an eigenvalue greater than one. Next, to compare scores from the nine facility readiness indices, we categorized the continuous index scores into low, medium, and high readiness categories using tercile cut points. We then assessed the comparability of the facility classification across the indices by calculating the percent agreement and Cohen's kappa, which accounts for the possibility of the agreement occurring by chance, between each combination of indices. Cohen's kappa ranges from -1 to 1 where < 0 means no agreement and 1 means perfect agreement [39].

Next, we examined the variability and distribution of the index scores. As countries are interested in being able to compare facilities with each other to understand better and worse performers (even if they are all with the low- or high-quality band), we are interested in being able to capture that variation in an index score. The examination of variability and distribution of the index scores aims to understand the level of variability being captured but does not indicate that an index is necessarily "better" because it captures more variability. The coefficient of variation (CoV) was used to measure the variation captured by each index, with a higher CoV

For peer review only - http://bmjopen.bmj.com/site/about/guidelines.xhtml

indicative of more variation captured in the index. Distribution of the scores was examined by assessment of floor and ceiling effects. Floor and ceiling effects were considered to be present if more than 15% of facilities achieved the lowest or highest possible index score (0% or 100%) respectively [40]. While in some settings it may be possible that greater than 15% of facilities are at 0% or 100%, the presence of floor and ceiling effects may indicate that indices have limited ability to differentiate facilities at very low and very high readiness levels. Finally, each index was assessed in terms of inclusion of items with a range of variability. For each index, we tallied the number of items that were available in <30% of facilities, <40% of facilities, and >90% of facilities.

All statistical analyses were carried out using R version 3.5.1 [41]. The institutional review board at the Johns Hopkins Bloomberg School of Public Health determined this analysis to be exempt from human subjects review.

Patient and Public Involvement

It was not appropriate or possible to involve patients or the public in this work.

Results

The final analytic sample consisted of 358 health facilities and 779 ANC clients in Haiti, 253 health facilities and 815 ANC clients in Malawi, and 632 health facilities and 1,681 ANC clients in Tanzania. A total of 6 ANC clients in Haiti, 44 ANC clients in Malawi, and 73 ANC clients in Tanzania were excluded from the analysis due to missing data.

Item selection

The results of the expert survey are available in (***Supplementary Table 4).*** There was some alignment between the core set of readiness items and the expert set of readiness items. The core readiness index included 21 items, the expert readiness index included items, and the

For peer review only - http://bmjopen.bmj.com/site/about/guidelines.xhtml

overlap between the two indices was 14 items. Of the 45 facility readiness items identified for inclusion in the expert survey, only four were related to human resources and of those, only one was highly rated by the expert group as essential for high quality service delivery. In addition, the expert survey respondents mentioned a number of items that they felt were essential for high quality care but were missing from our survey and from SPA datasets. These items included: ensuring that clients are treated with respect and without discrimination, having the option for clients to invite their partner to participate in the consultation, discussing identification of a birth companion of choice with the client, including family members in counselling sessions, providing counselling on newborn care practices, and ensuring clients understand the counselling information they receive.

Descriptive statistics

The mean readiness score varied by index from 56.6 to 64.1 in Haiti, 52.3 to 69.6 in Malawi, and 61.2 to 75.9 in Tanzania (***Table 1, Figure 2***). Across all countries, there were no indices for which any facilities received the minimum score of zero and all countries had at least three indices for which some facilities received the maximum score of 100. In addition, in each country, the three indices that contained the same items — but used different methodologies for combining the items — generally had a similar median. For example, the core simple, core weighted, and core PCA median scores in Tanzania were 73.5, 72.1, and 73.9, respectively. Finally, the indices created using PCA resulted in a larger inter-quartile range (IQR), particularly for the core and expert item indices, across all three countries.

The mean provision of care score varied by index from 38.9 to 85.9 in Haiti, 35.8 to 53.1 in Malawi, and 40.4 to 58.0 in Tanzania (***Table 1, Figure 3***). The expert PCA index in Haiti was the only index in which any clients received the minimum score of zero and no countries had an

For peer review only - http://bmjopen.bmj.com/site/about/guidelines.xhtml

index for which any clients received the maximum score of 100. The expert simple and expert weighted indices had similar median provision of care scores; however, the expert weighted index scores were higher than the simple index scores in all three countries. The expert PCA index scores had median provision of care scores which were very different from both the expert simple and expert weighted scores. For example, in Malawi the expert simple mean provision of care score was 46.9, the expert weighted mean provision of care score was 53.7, and the expert PCA mean provision of care score was 33.9.

The results from the PCA analysis are presented in ***Table 2***. Across all countries, and all indices for facility readiness, the items with the highest loadings (absolute value greater than 0.4) differed by index and country. For the core item indices, the items that loaded the highest were related to diagnostics and human resources. For the expert item indices, the items that loaded the highest were related to diagnostics. For the maximum item indices, the items that loaded the highest were related to diagnostics and basic amenities. For all facility readiness indices in all countries, the percent of variance explained by the first principal component was low, ranging from 17.21% for the core and maximum indices in Haiti to 19.49% and 20.38% for the expert set of items in Tanzania and Haiti, respectively. In addition, we found that some items such as tetanus toxoid vaccine, de-worming medications, and syphilis testing had negative loadings.

Across all countries for provision of care, the items with the highest loadings (absolute value greater than 0.4) differed by country. For Haiti, the items that loaded the highest were related to client experience. For Malawi and Tanzania, the items that loaded the highest were related to history-taking and counselling; although for each country, the highest loadings were largely on different items. For provision of care indices in all countries, the percent of variance explained by the first principal component was low, ranging from 9.87% in Malawi to 19.49% in

For peer review only - http://bmjopen.bmj.com/site/about/guidelines.xhtml
For peer review only

Haiti. In addition, we found that items such as history-taking, client education and counselling on danger signs in pregnancy, and hemoglobin testing had negative loadings.

Comparability of the Facility Classification

Table 3 presents the results of the percent agreement and Cohen's kappa coefficient among the nine facility readiness indices. Across all countries, all readiness indices had fair or better agreement. In Haiti, there were three index combinations that had fair agreement (core weighted/core PCA; core weighted/expert PCA; core weighted/maximum PCA) while the remainder of the indices had moderate or better agreement. In Malawi, there was one index combination that had fair agreement (core weighted/maximum simple) while the remainder of the indices had moderate or better agreement. In Tanzania, there were two index combinations that had fair agreement (core weighted/expert PCA; core weighted/maximum PCA) while the remainder of the indices had moderate or better agreement.

Variability and Distribution of the Index

The variability and distribution of the nine facility readiness indices are presented in ***Table 4***. In Haiti, the CoV ranged from 21.12 (maximum simple readiness index) to 35.62 (expert PCA). In Malawi, the CoV ranged from 18.96 (expert PCA) to 32.09 (core PCA). In Tanzania, the CoV ranged from 19.11 (core simple) to 23.62 (expert PCA). Across all countries and all indices, there were no floor effects. Ceiling effects were limited and far below the 15% threshold. The highest percentage of ceiling effects was 3.33% which was found in Tanzania in three indices (expert simple, expert weighted, and expert PCA).

Inclusion of Items Across a Range of Frequency

Table 5 presents the percentage of facilities in which each index item was available in order to assess the inclusion of items across a range of frequency. Across all countries, the

For peer review only - http://bmjopen.bmj.com/site/about/guidelines.xhtml

maximum item index contained the greatest number of items available in less than 40% of facilities (10 items Haiti, 12 items Malawi, and nine items in Tanzania). In Tanzania, the expert index did not include any items available in less than 40% of facilities. Within countries, the core, expert, and maximum indices had a similar number of items that were available in over 90% of facilities (four to five in Haiti, six to eight in Malawi, seven to nine in Tanzania).

Discussion

To our knowledge, this is the first study that uses multiple methodologies to develop and compare indices for ANC facility readiness and provision of care. Our results showed that, within a country, indices containing different items combined using different approaches were quite similar in terms of mean index score, facility classification, coefficient of variation, floor and ceiling effects, and inclusion of items with a range of variability. Although similar overall, we found that the indices created using PCA were the most different from the other indices. In addition, the core weighted index had lower agreement with the other indices when looking at facility classification. This may be the result of some domains in the core weighted index having few items, making each of those items more influential in the overall index score.

Our analysis highlighted the importance of competent, motivated human resources to all aspects of delivering high quality ANC services. Our approach to defining the core set of items highlighted the lack of metrics for human resources captured by facility surveys, as well as the inconsistency between what inputs are required for provision of care and how facility readiness is often defined. This is especially important considering there is a health workforce crisis globally which is particularly pronounced in LMICs [42-44]. Many countries are suffering from an absolute shortage of health care workers. The health workers they do have are often poorly

For peer review only - http://bmjopen.bmj.com/site/about/guidelines.xhtml

distributed within a country and many workers are deficient in skill mix and core competencies [45 46].

This study's findings also suggest that to provide more comprehensive measures of ANC quality some items could be added to SPAs and other facility surveys, particularly items related to the experience of care. While the global community is moving toward more person-centered care, data collected through health facility assessments is generally deficient in experience of care measures [47 48]. This may be due to the lack of validated instruments available for measuring the experience of maternity care [49]. In addition, measures of patient experience from health facility assessments are limited and may be subject to courtesy bias which has been found to be particularly problematic for subjective questions regarding items such as treatment by staff and consultation quality, items of interest for measuring experience of care [50]. We therefore cannot be certain if these measures are actually representative of people's experiences or if other types of questions would better capture experience of care. However, a number of recent studies have generated validated tools for measuring respectful maternity care; these may well provide a starting point for incorporating improved experience of care metrics in health facility assessments such as the SPA [51 52].

We also found that the indices differed in terms of their ease of construction and interpretation. The simple additive and weighted additive approaches were straightforward to construct because they required taking averages of items, while the PCA necessitated more complex analysis. In addition, the simple additive approach was the easiest to interpret since facilities with more items available had a higher quality score. The weighted additive approach was also relatively easy to interpret. However, item weighting whereby each domain receives an equal weight was based on an implicit conceptual framework for quality which has not been

For peer review only - http://bmjopen.bmj.com/site/about/guidelines.xhtml

formally validated. The PCA approach was perhaps the most difficult to interpret as the PCA score represents the linear combination of variables that explained the most variance in the data. As the loadings of each variable on the first component were used as the weights, and some items were negatively correlated and consequently had negative loadings; having all items present in a facility did not always result in the highest PCA score. In addition, the weights assigned by PCA reflected the variation in the data which was different across countries. As a result, PCA did not produce the same weights across different contexts. As we were working with a common definition for what an ANC visit should include across country contexts, this variability in loadings resulted in concerns regarding the face validity and construct validity of the PCA indices. Overall, the low percent of total variance explained by the first principle component, diverging items with high loadings between countries, negatively correlated items, and the complexity in creating and interpreting the measure highlights concerns for the use of PCA for creating quality of care indices [53]. Another study focused on comparing summary measures of quality of care for family planning found similar results in terms of the ease of construction and interpretation of similar indices suggesting this finding may be relevant across service areas[54].

Due the variation in ease of construction and interpretation, different methodologies may be better suited for different purposes. For example, the simple additive approach using the maximum number of items may be easiest for Ministries of Health in LMICs to understand and implement. However, if there is particular interest in using facility readiness as a proxy for provision of care, it might be conceptually important to ensure that items align well between facility readiness and provision of care thus making the core set of items a good choice. In addition, while the weighted additive method aims to give equal weights to domains of facility

For peer review only - http://bmjopen.bmj.com/site/about/guidelines.xhtml

readiness and provision of care, as the contents of each domain are determined by what data is available as opposed to an ideal set of items, this approach implicitly weights certain items more heavily because of the unavailability of data on other items and therefore may not be well-suited depending on the purpose of the index. We note several limitations of the analysis. First, this analysis was conducted using data from three countries, two in Africa and one in Latin America and the Caribbean, which may limit generalizability of findings to low-income countries globally. However, the findings were relatively consistent across the three countries. The two African countries are in the Southern and Eastern Africa region and represent two different types and sizes of health system within that region. Second, the SPA survey, while quite comprehensive, does not capture every aspect of quality of care that may be important to include in quality of care metrics. However, the SPA represents the most comprehensive information on quality of care in LMICs that is currently available[34]. Third, our approaches to item selection and combination were based on the most commonly used approaches in the literature. However, there are other methods that may be helpful in determining which items to include in a quality of care index, such as latent class analysis, that we did not implemented. The selection of methods for this analysis included those most likely to be useful to LMICs. Finally, this analysis does not provide a formal validation of any one ANC quality of care index. However, it does characterize various indices of ANC summary indices and provides valuable information on the similarities and differences between indices.

Conclusion

While the goal of this study was not to identify the best index for measuring quality of care, we did endeavor to characterize the various indices to make more information available in order to assist health systems researchers in choosing a methodology for creating quality of care

For peer review only - http://bmjopen.bmj.com/site/about/guidelines.xhtml

indices. In addition, we found that different methodologies may be better suited for different purposes. Future research on the association between facility readiness and provision of care would be helpful to further characterize the quality of care indices and inform selection of an index. As quality of care becomes integral to global efforts to improve health outcomes, demand for guidance on creating standardized measures of service quality will grow. This study provides health systems researchers with a comparison of methodologies commonly used to create summary indices of ANC service quality and highlights the similarities and differences between methods.

For peer review only

For peer review only - http://bmjopen.bmj.com/site/about/guidelines.xhtml

Acknowledgements

The authors wish to acknowledge the Bill & Melinda Gates Foundation for their support of this project. In addition, we would like to thank the maternal health experts who participated in the expert survey; we appreciate your time and willingness to share your expertise.

Declaration of Interests

Dr. Munos reports grants from Bill and Melinda Gates Foundation during the conduct of the study.

Role of the Funding Source

The funder had no role in the study design, data collection, analysis and interpretation of data, or manuscript writing.

Data Availability Statement

Data are available in a public, open access repository. Data is available from the DHS program after registering as a user and submitting information regarding your research purpose. The DHS program website is: https://dhsprogram.com/

For peer review only

References

1. Countdown to 2015: Maternal Newborn and Child Survival. Fulfilling the Health Agenda for Women and Children: The 2014 Report, 2014.
2. Souza JP, Gulmezoglu AM, Vogel J, et al. Moving beyond essential interventions for reduction of maternal mortality (the WHO Multicountry Survey on Maternal and Newborn Health): a cross-sectional study. *Lancet* 2013;381(9879):1747-55. doi: 10.1016/s0140-6736(13)60686-8 [published Online First: 2013/05/21]
3. Sobel HL, Huntington D, Temmerman M. Quality at the centre of universal health coverage. *Health Policy Plan* 2016;31(4):547-9. doi: 10.1093/heapol/czv095 [published Online First: 2015/10/01]
4. Kruk ME, Larson E, Twum-Danso NAY. Time for a quality revolution in global health. *The Lancet Global Health* 2016;4(9) doi: 10.1016/S2214-109X(16)30131-0
5. Villar J, Bergsjø P. Scientific basis for the content of routine antenatal care. I. Philosophy, recent studies, and power to eliminate or alleviate adverse maternal outcomes. *Acta obstetricia et gynecologica Scandinavica* 1997;76(1):1-14.
6. Campbell O, Graham W. Strategies for reducing maternal mortality: getting on with what works. *The Lancet* 2006;368(9543):1284-99. doi: 10.1016/S0140-6736(06)69381-1
7. Lassi ZS, Salam RA, Das JK, et al. Essential pre-pregnancy and pregnancy interventions for improved maternal, newborn and child health. *Reproductive Health* 2014;11(Suppl 1):S2-S2. doi: 10.1186/1742-4755-11-S1-S2
8. Callaghan-Koru JA, McMahon SA, Chebet JJ, et al. A qualitative exploration of health workers' and clients' perceptions of barriers to completing four antenatal care visits in Morogoro Region, Tanzania. *Health policy and planning* 2016;31(8):1039-49. doi: 10.1093/heapol/czw034
9. Gross K, Schellenberg Armstrong J, Kessy F, et al. Antenatal care in practice: an exploratory study in antenatal care clinics in the Kilombero Valley, south-eastern Tanzania. *BMC Pregnancy and Childbirth* 2011;11:36:1-11.
10. Gupta S, Yamada G, Mpembeni R, et al. Factors associated with four or more antenatal care visits and its decline among pregnant women in Tanzania between 1999 and 2010. *PLoS ONE* 2014;9(7) doi: 10.1371/journal.pone.0101893

For peer review only - http://bmjopen.bmj.com/site/about/guidelines.xhtml

11. Donabedian A. The Quality of Care: How Can It Be Assessed? *JAMA* 1988;260(12):1743-43. doi: 10.1001/jama.1988.03410120089033

12. Donabedian A. Evaluating the quality of medical care. *The Milbank quarterly* 2005;83(4):691-729. doi: 10.1111/j.1468-0009.2005.00397.x

13. Donabedian A. The Definition of Quality and Approaches to Its Assessment Vol 1. Explorations in Quality Assessment and Monitoring. Ann Arbor, Michigan: Health Administration Press 1980.

14. Institute of Medicine Committee on Quality of Health Care in America. Crossing the quality chasm : a new health system for the 21st century. Washington, DC: National Academy Press 2001.

15. World Health Organization. Quality of care: a process for making strategic choices in health systems. Geneva, Switzerland, 2006.

16. Bollen KA, Bauldry S. Three Cs in Measurement Models: Causal Indicators, Composite Indicators, and Covariates. *Psychol Methods* 2011;16(3):265-84. doi: 10.1037/a0024448

17. Sheffel A, Karp C, Creanga AA. Use of Service Provision Assessments and Service Availability and Readiness Assessments for monitoring quality of maternal and newborn health services in low-income and middle-income countries. *BMJ Global Health* 2018;3(6) doi: http://dx.doi.org/10.1136/bmjgh-2018-001011

18. Diamond-Smith N, Sudhinaraset M, Montagu D. Clinical and perceived quality of care for maternal, neonatal and antenatal care in Kenya and Namibia: the service provision assessment. *Reproductive Health* 2016;13(1):92-92. doi: 10.1186/s12978-016-0208-y

19. Do M, Wang W, Hembling J, et al. Quality of antenatal care and client satisfaction in Kenya and Namibia. *International Journal for Quality in Health Care* 2017;29(2):183-93. doi: 10.1093/intqhc/mzx001

20. Leslie HH, Fink G, Nsona H, et al. Obstetric Facility Quality and Newborn Mortality in Malawi: A Cross-Sectional Study. *PLoS medicine* 2016;13(10):e1002151. doi: 10.1371/journal.pmed.1002151

21. Kruk ME, Leslie HH, Verguet S, et al. Quality of basic maternal care functions in health facilities of five African countries: an analysis of national health system surveys. *The Lancet Global Health* 2016;4(11):e845-e55. doi: 10.1016/S2214-109X(16)30180-2

For peer review only

For peer review only - http://bmjopen.bmj.com/site/about/guidelines.xhtml

22. Leslie HH, Gage A, Nsona H, et al. Training And Supervision Did Not Meaningfully Improve Quality Of Care For Pregnant Women Or Sick Children In Sub-Saharan Africa. *Health Aff (Millwood)* 2016;35(9):1716-24. doi: 10.1377/hlthaff.2016.0261 [published Online First: 2016/09/09]

23. Leslie HH, Malata A, Ndiaye Y, et al. Effective coverage of primary care services in eight high-mortality countries. *BMJ Glob Health* 2017;2(3):e000424. doi: 10.1136/bmjgh-2017-000424 [published Online First: 2018/04/11]

24. Lee E, Madhavan S, Bauhoff S. Levels and variations in the quality of facility-based antenatal care in Kenya: evidence from the 2010 service provision assessment. *Health Policy and Planning* 2016;31(6):777-84. doi: 10.1093/heapol/czv132

25. Sharma J, Leslie HH, Kundu F, et al. Poor Quality for Poor Women? Inequities in the Quality of Antenatal and Delivery Care in Kenya. *PloS one* 2017;12(1):e0171236. doi: 10.1371/journal.pone.0171236

26. Kanyangarara M, Chou VB. Linking household surveys and health facility assessments to estimate intervention coverage for the Lives Saved Tool (LiST). *BMC public health* 2017;17(Suppl 4):780. doi: 10.1186/s12889-017-4743-4

27. Nguhiu PK, Barasa EW, Chuma J. Determining the effective coverage of maternal and child health services in Kenya, using demographic and health survey data sets: tracking progress towards universal health coverage. *Tropical Medicine & International Health* 2017;22(4):442-53. doi: 10.1111/tmi.12841

28. O'Neill K, Takane M, Sheffel A, et al. Monitoring service delivery for universal health coverage: the Service Availability and Readiness Assessment. *Bulletin of the World Health Organization* 2013;91(12):923-31. doi: 10.2471/BLT.12.116798

29. Sipsma HL, Curry LA, Kakoma J-B, et al. Identifying characteristics associated with performing recommended practices in maternal and newborn care among health facilities in Rwanda: a cross-sectional study. *Human Resources for Health* 2012;10(1):13-13. doi: 10.1186/1478-4491-10-13

30. Van Huy N, Nam YS, Van Thanh N, et al. WHO's Service Availability and Readiness Assessment of primary health care services of commune health centers in a rural district of Northern Vietnam. *International Journal of Health Planning and Management* 2017 doi: 10.1002/hpm.2429

For peer review only

For peer review only - http://bmjopen.bmj.com/site/about/guidelines.xhtml

31. Wang W, Winner M, Burgert-Brucker CR. Limited Service Availability, Readiness, and Use of Facility-Based Delivery Care in Haiti: A Study Linking Health Facility Data and Population Data. *Global Health: Science and Practice* 2017;5(2):244-60. doi: 10.9745/GHSP-D-16-00311

32. Hutchinson PL, Do M, Agha S. Measuring client satisfaction and the quality of family planning services: A comparative analysis of public and private health facilities in Tanzania, Kenya and Ghana. *BMC Health Services Research* 2011;11(1):203-03. doi: 10.1186/1472-6963-11-203

33. Ngo DKL, Sherry TB, Bauhoff S. Health system changes under pay-for-performance: the effects of Rwanda's national programme on facility inputs. *Health Policy and Planning* 2017;32(1):11-20. doi: 10.1093/heapol/czw091

34. ICF. The DHS Program: SPA Overview 2017 [Available from: http://dhsprogram.com/What-We-Do/Survey-Types/SPA.cfm.

35. Institut Haïtien de l'Enfance, ICF International. Évaluation de Prestation des Services de Soins de Santé, Haïti, 2013, 2014.

36. Malawi Ministry of Health, ICF International. Malawi Service Provision Assessment (MSPA) 2013-2014: MoH and ICF International, 2014.

37. Tanzania Ministry of Health & Social Welfare, Tanzania National Bureau of Statistics, ICF International. Tanzania Service Provision Assessment Survey 2014-2015, 2016.

38. World Health Organization. Standards for improving quality of maternal and newborn care in health facilities. Geneva, Switzerland, 2016.

39. Landis J, Koch G. The measurement of observer agreement for categorical data. *Biometrics* 1977;33(1):159-74.

40. Terwee CB, Bot SD, de Boer MR, et al. Quality criteria were proposed for measurement properties of health status questionnaires. *J Clin Epidemiol* 2007;60(1):34-42. doi: 10.1016/j.jclinepi.2006.03.012 [published Online First: 2006/12/13]

41. R: A Language and Environment for Statistical Computing [program]. Vienna, Austria: R Foundation for Statistical Computing, 2017.

42. Chen L, Evans T, Anand S, et al. Human resources for health: overcoming the crisis. *The Lancet* 2004;364(9449):1984-90.

For peer review only

For peer review only - http://bmjopen.bmj.com/site/about/guidelines.xhtml

43. Fulton BD, Scheffler RM, Sparkes SP, et al. Health workforce skill mix and task shifting in low income countries: a review of recent evidence. *Hum Resour Health* 2011;9:1. doi: 10.1186/1478-4491-9-1 [published Online First: 2011/01/13]

44. Campbell J, Dussault G, Buchan J, et al. A Universal Truth: No Health Without a Workforce. Recife, Brazil and Geneva,Switzerland: Global Health Workforce Alliance and World Health Organization, 2014.

45. Joint Learning Initiative. Human resources for health: overcoming the crisis. Cambridge, MA: Global Equity Initiative, Harvard University, 2004.

46. Ranson MK, Chopra M, Atkins S, et al. Priorities for research into human resources for health in low- and middle-income countries. *Bull World Health Organ* 2010;88(6):435-43. doi: 10.2471/blt.09.066290 [published Online First: 2010/06/12]

47. Sheffel A, Heidkamp R, Mpembeni R, et al. Understanding client and provider perspectives of antenatal care service quality: a qualitative multi-method study from Tanzania. *The Journal of Global Health* 2018; [submitted, under review]

48. Brizuela V, Leslie HH, Sharma J, et al. Measuring quality of care for all women and newborns: how do we know if we are doing it right? A review of facility assessment tools. *Lancet Glob Health* 2019;7(5):e624-e32. doi: 10.1016/s2214-109x(19)30033-6 [published Online First: 2019/03/23]

49. Bohren MA, Vogel JP, Hunter EC, et al. The Mistreatment of Women during Childbirth in Health Facilities Globally: A Mixed-Methods Systematic Review. *PLoS Med* 2015;12(6) doi: 10.1371/journal.pmed.1001847 [published Online First: 2015/07/01]

50. Glick P. How reliable are surveys of client satisfaction with healthcare services? Evidence from matched facility and household data in Madagascar. *Soc Sci Med* 2009;68(2):368-79. doi: 10.1016/j.socscimed.2008.09.053 [published Online First: 2008/11/26]

51. Vedam S, Stoll K, Martin K, et al. The Mother's Autonomy in Decision Making (MADM) scale: Patient-led development and psychometric testing of a new instrument to evaluate experience of maternity care. *PLoS One* 2017;12(2):e0171804. doi: 10.1371/journal.pone.0171804 [published Online First: 2017/02/24]

52. Vedam S, Stoll K, Rubashkin N, et al. The Mothers on Respect (MOR) index: measuring quality, safety, and human rights in childbirth. *SSM - Population Health* 2017;3:201-10. doi: https://doi.org/10.1016/j.ssmph.2017.01.005

For peer review only - http://bmjopen.bmj.com/site/about/guidelines.xhtml

53. Sharker MY, Nasser M, Abedin J, et al. The risk of misclassifying subjects within principal component based asset index. *Emerging themes in epidemiology* 2014;11:6. doi: 10.1186/1742-7622-11-6 [published Online First: 2014/07/06]

54. Mallick L, Temsah G, Wang W. Comparing summary measures of quality of care for family planning in Haiti, Malawi, and Tanzania. *PLoS One* 2019;14(6):e0217547. doi: 10.1371/journal.pone.0217547 [published Online First: 2019/06/08]

For peer review only

TABLES

Table 1: Descriptive Statistics of Facility Readiness and Provision of Care Indices

	Facility Readiness Indices									Provision of Care Indices		
	core simple	core weighted	core PCA	expert simple	expert weighted	expert PCA	maximum simple	maximum weighted	maximu m PCA	simple	weighted	PCA
**Haiti (N = 358 facilities)**										**Haiti (N = 779 clients)**		
mean	63.8	64.0	59.4	64.1	61.3	58.2	57.8	56.6	59.8	38.9	45.0	85.9
SD	14.0	15.1	21.4	16.1	16.6	21.6	12.6	14.0	15.9	8.0	10.4	15.9
median	63.3	65.5	59.4	64.3	61.3	57.5	57.9	57.4	60.6	38.8	45.4	91.7
min	23.8	16.4	17.6	17.1	18.1	12.9	27.6	20.1	23.4	12.2	14.0	0.0
max	92.9	93.8	99.8	100.0	100.0	100.0	87.5	87.7	95.2	79.6	76.3	96.0
IQR	19.8	20.7	40.9	24.6	24.2	39.5	17.8	20.2	24.6	8.5	12.9	7.1
**Malawi (N = 253 facilities)**										**Malawi (N = 815 clients)**		
mean	62.9	65.2	52.3	68.7	67.6	69.6	61.3	61.0	63.1	47.5	53.1	35.8
SD	13.2	13.3	16.7	13.2	13.1	13.2	11.5	11.4	12.9	9.9	10.2	14.5
median	61.9	66.4	46.4	68.4	67.8	66.3	59.9	60.0	59.8	46.9	53.7	33.9
min	33.3	30.0	24.5	38.2	34.3	38.9	35.5	32.7	37.0	22.4	22.9	10.7
max	100.0	100.0	100.0	100.0	100.0	100.0	92.8	94.3	100.0	72.0	75.5	70.8
IQR	19.0	16.7	20.5	17.5	16.9	16.5	16.7	14.1	18.7	14.3	13.5	24.5
**Tanzania (N = 632 facilities)**										**Tanzania (N = 1,681 clients)**		
mean	71.4	71.0	70.2	75.9	71.8	74.7	61.2	62.8	64.1	50.8	58.0	40.4
SD	13.6	14.6	15.5	15.4	14.6	17.8	13.0	13.6	14.6	10.9	11.2	12.3
median	73.5	72.1	73.9	78.9	73.2	78.4	61.8	63.9	64.9	50.5	58.6	39.5
min	23.8	18.6	20.4	28.9	28.6	20.3	27.6	25.5	24.7	16.3	18.5	9.9
max	100.0	100.0	100.0	100.0	100.0	100.0	94.7	92.1	100.0	98.0	99.3	98.2
IQR	21.4	20.3	25.8	23.8	20.2	30.2	20.2	19.9	23.0	14.8	15.1	17.0

For peer review only - http://bmjopen.bmj.com/site/about/guidelines.xhtml

Table 2: Results from the Facility Readiness Indices and Provision of Care Indices Principal Component Analyses

Facility Readiness Indices									
Item name	Haiti			Malawi			Tanzania		
	core set	expert set	maximum set	core set	expert set	maximum set	core set	expert set	maximum set
Equipment and supplies									
Blood pressure apparatus	0.05	0.04	0.04	0.37	0.34	0.37	0.34	0.33	0.28
Examination light	0.08		0.14	0.44		0.49	0.34		0.38
Stethoscope	0.14	0.11	0.17	0.16	0.13	0.18	0.28	0.23	0.23
Adult weighing scale	0.22		0.19	0.25		0.32	0.43		0.39
Tape measure for fundal height	0.13		0.18	0.22		0.28	0.21		0.19
Examination bed	0.23	0.22	0.26	0.17	0.21	0.18	0.14	0.10	0.15
Latex gloves	0.24	0.52	0.49	0.14	0.32	0.39	0.08	0.50	0.51
Single use syringes		0.25	0.26		0.24	0.21		0.14	0.14
Soap and running water OR Alcohol based hand rub		0.41	0.41		0.38	0.40		0.52	0.57
Disinfectant		0.40	0.43		0.34	0.30		0.32	0.30
Appropriate storage of sharps waste (sharps box)			-0.12			0.09			0.11
Appropriate storage of infectious waste (pedal bin with lid)			0.01			0.03			0.17
Safe final disposal of sharps (incineration)			0.20			0.26			0.06
Safe final disposal of infectious wastes (incineration)			0.20			0.25			0.06
Medical masks			0.06			0.16			0.35
Gowns			0.31			0.21			0.38
Eye protection			0.29			0.28			0.23
Diagnostics									
Hemoglobin	0.63	0.61	0.60	0.75	0.75	0.70	0.72	0.71	0.69
Urine dipstick- protein	0.78	0.77	0.69	0.85	0.84	0.77	0.77	0.78	0.68
Urine dipstick- glucose	0.74	0.73	0.66	0.80	0.78	0.71	0.78	0.79	0.69
Grouping and Rhesus factor	0.18		0.19	0.53		0.47	0.29		0.37
Syphilis RDT / RPR	0.78	0.77	-0.04	0.68	0.68	-0.05	0.65	0.65	-0.04
HIV testing / RDT	0.78	0.79	0.72	0.18	0.23	0.21	0.41	0.41	0.35
Medicines									
Iron tablets	0.10	0.11	0.18	0.11	0.17	0.15	0.26	0.26	0.25
Folic acid tablets	0.15	0.16	0.19	0.16	0.24	0.22	0.20	0.21	0.21
TT vaccine	-0.05	-0.02	0.01	0.20	0.25	0.24	0.33	0.30	0.27
De-worming drugs	0.09		0.12	-0.12		-0.06	0.15		0.09
Basic Amenities									
Power		0.18	0.55		0.17	0.29		0.44	0.41
Improved water source		-0.08	0.23		0.10	0.14		0.03	0.39
Room with auditory and visual privacy	-0.10	0.46	-0.07	0.06	0.20	0.06	0.08	0.26	0.06
Sanitation facilities			0.42			0.10			0.25
Communication equipment			0.25			0.27			0.60
Computer with email/internet			0.62			0.71			0.66
Emergency transportation			0.38			0.01			0.33
Human resources									
Guidelines ANC	-0.06		0.00	0.15		0.20	0.38	0.33	0.36
Proportion of staff trained in ANC in last two years	0.43	0.39	0.39	0.47	0.46	0.44	0.40		0.34
Proportion of staff receiving supervision in the last six months	0.60		0.59	0.43		0.40	0.42		0.37

For peer review only - http://bmjopen.bmj.com/site/about/guidelines.xhtml

Proportion of staff reporting opportunities for promotion			0.17			-0.05			-0.07
**Eigenvalue**	3.61	3.87	3.61	3.74	3.42	3.74	3.66	3.70	3.66
**Percent of total variance**	17.21	20.38	17.21	17.80	17.98	17.80	17.44	19.49	17.44

Provision of Care Indices

Item name	Haiti	Malawi	Tanzania
History and counselling			
History-taking: Personal history: client age	0.03	0.24	0.28
History-taking: Personal history: medications client is taking	-0.02	0.05	0.22
History-taking: Personal history: date last menstrual period began	0.02	0.25	0.32
History-taking: Personal history: any prior pregnancy	-0.09	0.44	0.31
History-taking: Past medical history for prior pregnancies: still birth	-0.18	0.69	0.46
History-taking: Past medical history for prior pregnancies: infant died in the first week of life	-0.01	0.59	0.45
History-taking: Past medical history for prior pregnancies: heavy bleeding during or after delivery	0.03	0.73	0.50
History-taking: Past medical history for prior pregnancies: previous assisted delivery	-0.02	0.73	0.42
History-taking: Past medical history for prior pregnancies: previous spontaneous abortion	0.00	0.69	0.39
History-taking: Past medical history for prior pregnancies: previous multiple pregnancies	0.02	0.54	0.37
History-taking: Past medical history for prior pregnancies: previous prolonged labor	0.00	0.44	0.39
History-taking: Past medical history for prior pregnancies: pregnancy-induced hypertension	-0.13	0.73	0.35
History-taking: Past medical history for prior pregnancies: pregnancy related convulsions	0.00	0.68	0.37
History-taking: Past medical history for prior pregnancies: high fever or infection during prior pregnancy/pregnancies	-0.04	0.21	0.35
History-taking: History of complaints in current pregnancy: vaginal bleeding	0.01	0.31	0.58
History-taking: History of complaints in current pregnancy: fever	0.07	0.15	0.51
History-taking: History of complaints in current pregnancy: headache or blurred vision	0.03	0.18	0.56
History-taking: History of complaints in current pregnancy: swollen face or hands or extremities	0.03	0.25	0.48
History-taking: History of complaints in current pregnancy: tiredness or breathlessness	-0.02	0.17	0.51

For peer review only

History-taking: History of complaints in current pregnancy: fetal movement (loss of, excessive, normal)	0.04	0.10	0.47
History-taking: History of complaints in current pregnancy: cough or difficulty breathing for 3 weeks or longer	-0.04	0.21	0.36
Client education and counselling: Process of pregnancy and its complications	0.01	0.16	0.27
Client education and counselling: Diet and nutrition	0.03	0.20	0.28
Client education and counselling: Danger signs in pregnancy	-0.01	0.14	0.65
Client education and counselling: Voluntary counselling and testing for HIV	0.08	0.12	0.27
Client education and counselling: Breast feeding	-0.02	0.07	0.29
Client education and counselling: Plans of delivery (emergency preparedness, place of delivery, transportation, financial arrangements)	0.07	0.28	0.37
Examination			
Examination and observation: Edema (other than ankle-specify)	0.08	0.21	0.24
Examination and observation: Blood pressure	0.03	0.09	0.10
Examination and observation: Weight	0.06	0.11	0.16
Obstetric complications: Palpate the client's abdomen for fundal height	0.15	0.11	0.19
Diagnostics			
Laboratory investigations: Hemoglobin	0.03	-0.01	0.12
Laboratory investigations: Grouping and rhesus factor	0.04	0.15	0.16
Laboratory investigations: RPR (syphilis test)	0.03	0.04	0.16
Laboratory investigations: HIV testing	0.04	0.04	0.23
Laboratory investigations: Urine- protein, sugar, acetone	0.03	0.03	0.09
Preventative treatment			
Drug administration and immunization: Iron and or folic acid provided or prescribed	0.06	0.13	0.18
Drug administration and immunization: Tetanus toxoid provided or prescribed	0.06	0.03	0.09
Client experience			
No problem: discuss problems or concerns	0.65	-0.01	0.14
No problem: explanation you received	0.64	0.08	0.15
No problem: how the staff treated you	0.73	0.05	0.09
No problem: privacy from having others see	0.72	0.07	0.20
No problem: privacy from having others hear	0.74	0.10	0.20
No problem: time you waited	0.44	0.04	0.01
No problem: number of days services are available	0.73	0.01	0.11
No problem: hours of service	0.75	0.00	0.06
No problem: cost for services or treatments	0.65	0.10	0.08

For peer review only - http://bmjopen.bmj.com/site/about/guidelines.xhtml

No problem: availability of medicines	0.62	0.02	0.06
No problem: cleanliness of the facility	0.69	0.03	0.06
**Eigenvalue**	5.14	4.84	5.08
**Percent of total variance**	10.49	9.89	10.37

For peer review only

For peer review only - http://bmjopen.bmj.com/site/about/guidelines.xhtml

Table 3: Percent Agreement and Cohen's Kappa Coefficient Among Nine Facility Readiness Indices

Haiti

	Core simple		Core weighted		Core PCA		Expert simple		Expert weighted		Expert PCA		Maximum simple		Maximum weighted	
	% Agreement	Kappa	% Agreement	Kappa	% Agreement	Kappa	% Agreement	Kappa	% Agreement	Kappa	% Agreement	Kappa	% Agreement	Kappa	% Agreement	Kappa
Core simple																
Core weighted	67.91	0.52														
Core PCA	69.96	0.55	51.48	0.27												
Expert simple	79.85	0.70	65.60	0.48	74.45	0.62										
Expert weighted	74.97	0.62	65.60	0.48	66.37	0.50	77.41	0.66								
Expert PCA	71.89	0.58	53.53	0.30	86.01	0.79	81.77	0.73	67.01	0.51						
Maximum simple	77.02	0.66	62.64	0.44	63.54	0.45	75.87	0.64	68.68	0.53	68.29	0.52				
Maximum weighted	82.54	0.74	65.98	0.49	67.91	0.52	77.79	0.67	73.68	0.61	69.58	0.54	84.47	0.77		
Maximum PCA	68.68	0.53	51.73	0.28	79.46	0.69	75.48	0.63	65.08	0.48	81.90	0.73	76.77	0.65	74.58	0.62

Malawi

	Core simple		Core weighted		Core PCA		Expert simple		Expert weighted		Expert PCA		Maximum simple		Maximum weighted	
	% Agreement	Kappa	% Agreement	Kappa	% Agreement	Kappa	% Agreement	Kappa	% Agreement	Kappa	% Agreement	Kappa	% Agreement	Kappa	% Agreement	Kappa
Core simple																
Core weighted	75.58	0.63														
Core PCA	82.09	0.73	65.40	0.48												
Expert simple	78.04	0.67	65.28	0.48	71.90	0.58										
Expert weighted	71.66	0.57	66.01	0.49	66.38	0.50	74.48	0.62								
Expert PCA	76.69	0.65	62.70	0.44	79.51	0.69	81.10	0.72	70.18	0.55						
Maximum simple	73.13	0.60	59.88	0.40	74.85	0.62	70.67	0.56	63.68	0.45	70.18	0.55				
Maximum weighted	77.42	0.66	64.91	0.47	77.55	0.66	73.50	0.60	65.03	0.48	70.31	0.55	79.51	0.69		
Maximum PCA	79.02	0.69	63.19	0.45	86.63	0.80	78.40	0.68	66.75	0.50	80.86	0.71	82.58	0.74	80.61	0.71

For peer review only - http://bmjopen.bmj.com/site/about/guidelines.xhtml

Tanzania	Core simple		Core weighted		Core PCA		Expert simple		Expert weighted		Expert PCA		Maximum simple		Maximum weighted	
	% Agreement	Kappa	% Agreement	Kappa	% Agreement	Kappa	% Agreement	Kappa	% Agreement	Kappa	% Agreement	Kappa	% Agreement	Kappa	% Agreement	Kappa
Core simple																
Core weighted	75.19	0.63														
Core PCA	88.28	0.82	66.57	0.50												
Expert simple	72.69	0.59	63.36	0.45	76.80	0.65										
Expert weighted	70.55	0.56	63.36	0.45	73.88	0.61	80.31	0.70								
Expert PCA	73.65	0.60	59.73	0.40	81.56	0.72	89.77	0.85	76.26	0.64						
Maximum simple	71.62	0.57	63.36	0.45	72.75	0.59	79.83	0.70	71.62	0.57	77.57	0.66				
Maximum weighted	77.33	0.66	65.91	0.49	77.51	0.66	75.01	0.63	71.92	0.58	74.30	0.61	84.47	0.77		
Maximum PCA	71.45	0.57	59.55	0.39	76.74	0.65	80.55	0.71	72.93	0.59	81.86	0.73	84.06	0.76	79.00	0.69

Legend	<0	0 — 0.20	0.21 — 0.40	0.41 — 0.60	0.61 — 0.80	0.81 — 1.0
	No agreement/ Worse than chance	Slight	Fair	Moderate	Good	Near perfect

For peer review only

Table 4: Variability and Distribution of Index Scores for the Nine Facility Readiness Indices

	Haiti			Malawi			Tanzania		
	CoV	Floor Effects	Ceiling Effects	CoV	Floor Effects	Ceiling Effects	CoV	Floor Effects	Ceiling Effects
Core simple	21.20	0.00	0.00	21.70	0.00	1.10	19.11	0.00	0.48
Core weighted	22.67	0.00	0.00	21.45	0.00	1.10	20.83	0.00	0.48
Core PCA	34.61	0.00	0.00	32.09	0.00	1.10	21.96	0.00	0.48
Expert simple	24.30	0.00	0.39	19.64	0.00	1.23	20.14	0.00	3.33
Expert weighted	26.01	0.00	0.39	19.82	0.00	1.23	20.24	0.00	3.33
Expert PCA	35.72	0.00	0.51	18.96	0.00	1.23	23.62	0.00	3.33
Maximum simple	21.12	0.00	0.00	19.33	0.00	0.00	21.31	0.00	0.00
Maximum weighted	24.07	0.00	0.00	18.98	0.00	0.00	21.69	0.00	0.00
Maximum PCA	26.02	0.00	0.00	20.67	0.00	1.10	22.73	0.00	0.36

For peer review only

Table 5: Inclusion of Items in an Index Across a Range of Frequencies for the Facility Readiness Indices

	Haiti			Malawi			Tanzania		
	No./% items <30% frequency	No./% items <40% frequency	No./% items >90% frequency	No./% items <30% frequency	No./% items <40% frequency	No./% items >90% frequency	No./% items <30% frequency	No./% items <40% frequency	No./% items >90% frequency
Core (21 items)	2 (10%)	4 (19%)	5 (24%)	4 (19%)	7 (33%)	6 (29%)	2 (10%)	2 (10%)	8 (38%)
Expert (19 items)	2 (11%)	3 (16%)	4 (21%)	2 (11%)	5 (26%)	7 (37%)	0 (0%)	0 (0%)	7 (37%)
Maximum (38 items)	8 (21%)	10 (26%)	5 (13%)	8 (21%)	12 (32%)	8 (21%)	5 (13%)	9 (24%)	9 (24%)

For peer review only

FIGURE TITLES AND LEGENDS

Figure 1: Process for Index Creation

- no legend

Figure 2: Distribution of Facility Readiness Scores

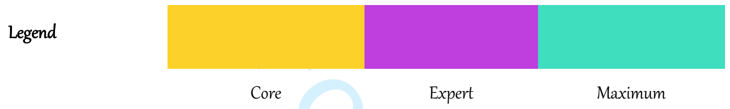

Figure 3: Distribution of Provision of Care Scores

- no legend

For peer review only

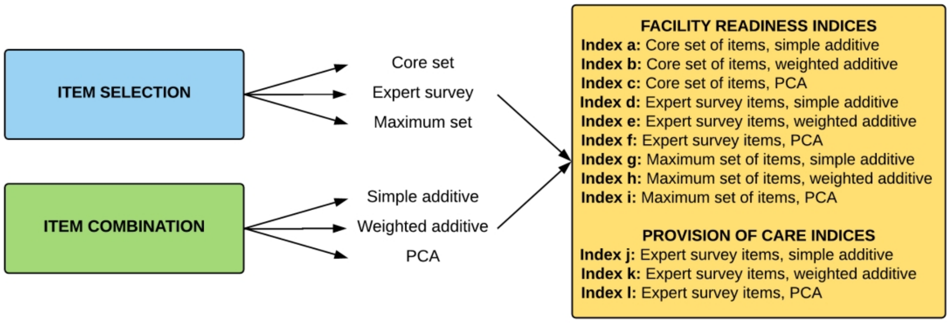
ITEM SELECTION
Core set
Expert survey
Maximum set
ITEM COMBINATION
Simple additive
Weighted additive
PCA
FACILITY READINESS INDICES
Index a: Core set of items, simple additive
Index b: Core set of items, weighted additive
Index c: Core set of items, PCA
Index d: Expert survey items, simple additive
Index e: Expert survey items, weighted additive
Index f: Expert survey items, PCA
Index g: Maximum set of items, simple additive
Index h: Maximum set of items, weighted additive
Index i: Maximum set of items, PCA
PROVISION OF CARE INDICES
Index j: Expert survey items, simple additive
Index k: Expert survey items, weighted additive
Index l: Expert survey items, PCA

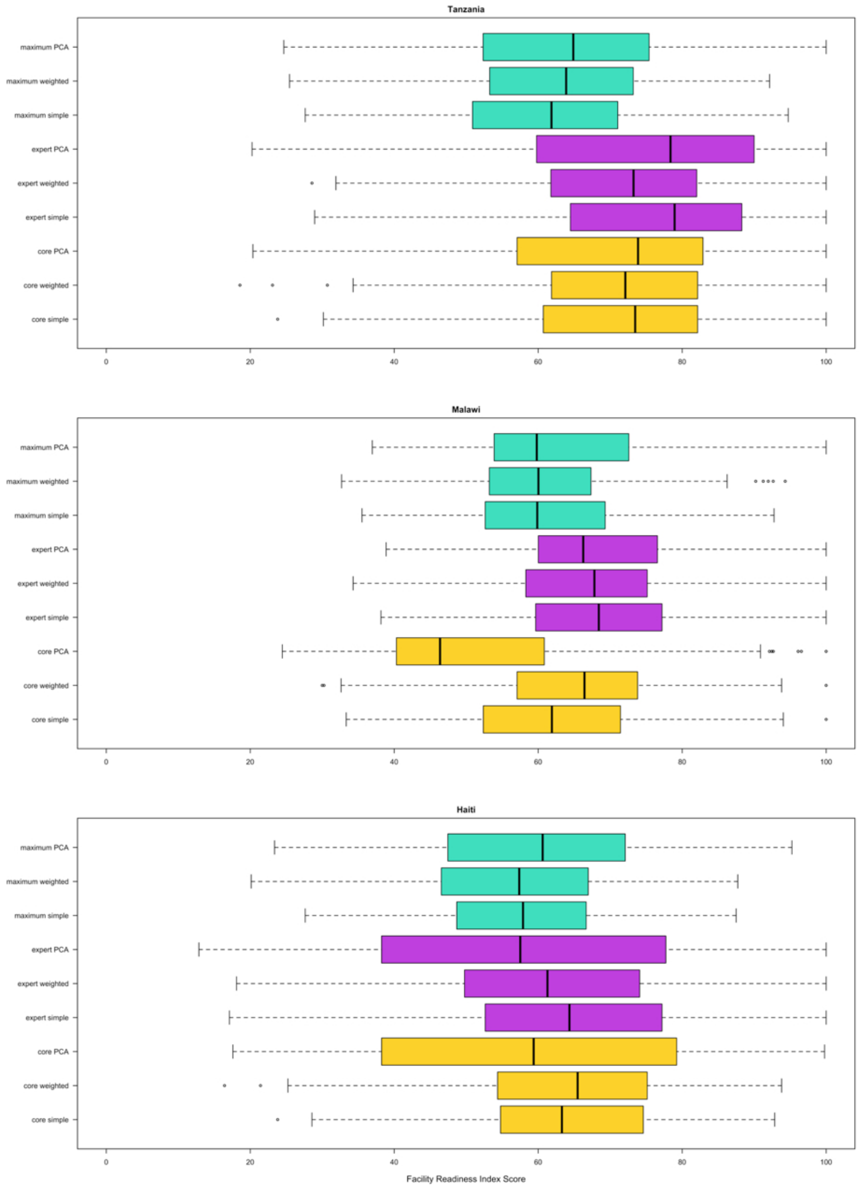
Tanzania
maximum PCA
maximum weighted
maximum simple
expert PCA
expert weighted
expert simple
core PCA
core weighted
core simple
0
20
40
60
80
100
Malawi
Haiti
Facility Readiness Index Score

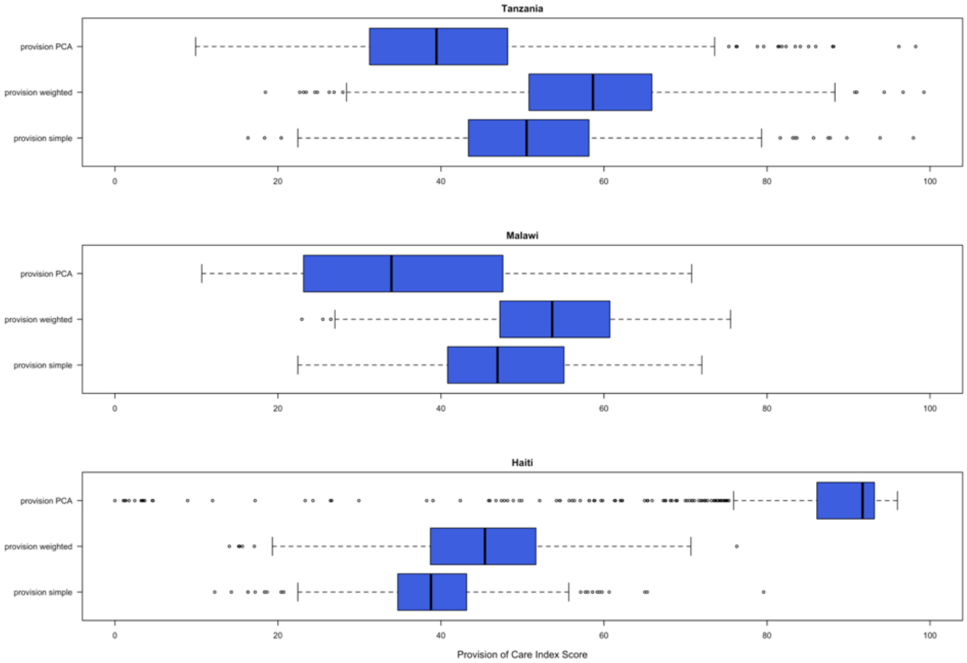
Tanzania
provision PCA
provision weighted
provision simple
0
20
40
60
80
100
Malawi
provision PCA
provision weighted
provision simple
0
20
40
60
80
100
Haiti
provision PCA
provision weighted
provision simple
0
20
40
60
80
100
Provision of Care Index Score

SUPPLEMENTARY TABLES

Supplementary Table 1: Summary of Literature Using SPA Data to Create ANC Quality of Care Indices

Author	Year	Country	Type of index and number of items	Item selection	Item combination
Diamond-Smith et al	2016	Kenya, Namibia	Facility readiness: 8 items	DHS tracer indicators of ANC quality	Simple additive
Do et al	2017	Kenya, Namibia	Facility readiness: 14 items Provision of care: 7 items	Donabedian framework and the WHO SARA indicators	Factor analysis
Kruk et al	2017	Kenya, Malawi, Namibia, Rwanda, Senegal, Uganda, Tanzania	Provision of care: 8 items	Clinical guidelines from the WHO	Simple additive
Lee et al	2016	Kenya	Facility readiness and provision of care: 14 items	WHO definition of quality of care	Weighted additive
Sharma et al	2017	Kenya	Facility readiness: 15 items Provision of care: 40 items	Donabedian framework, IOM definition of quality of care, and Focused ANC Model Checklist	Simple additive
Sipsma et al	2012	Rwanda	Provision of care: 8 items	Recommended practices derived from clinical guidelines	Simple additive

For peer review only

Supplementary Table 2: List of Items Included in the Facility Readiness Indices, by Domain

#	Item Name	Core set	Expert survey	Maximum set
	EQUIPMENT AND SUPPLIES<br>*Availability of...*			
1	Blood pressure apparatus	X	X	X
2	Examination light	X		X
3	Stethoscope	X	X	X
4	Adult weighing scale	X		X
5	Tape measure for fundal height	X		X
6	Examination bed	X	X	X
7	Latex gloves		X	X
8	Single use syringes	X	X	X
9	Soap and water OR alcohol-based hand rub		X	X
10	Disinfectant		X	X
11	Appropriate storage of sharps waste (sharps box)			X
12	Appropriate storage of infectious waste (pedal bin with lid and liner)			X
13	Safe final disposal of sharps (incineration)			X
14	Safe final disposal of infectious wastes (incineration)			X
15	Medical masks			X
16	Gowns			X
17	Eye protection			X
	DIAGNOSTICS<br>*Availability on-site to test for...*			
18	Hemoglobin	X	X	X
19	Urine dipstick- protein	X	X	X
20	Urine dipstick- glucose	X	X	X
21	Grouping and Rhesus factor	X		X
22	Syphilis RDT / RPR	X	X	X
23	HIV testing / RDT	X	X	X
	MEDICINES AND COMMODITIES<br>*Availability on-site of...*			
24	Iron tablets	X	X	X

For peer review only - http://bmjopen.bmj.com/site/about/guidelines.xhtml

#	Item Name	Core set	Expert survey	Maximum set
25	Folic acid tablets	X	X	X
26	Tetanus toxoid vaccine	X	X	X
27	De-worming drugs (mebendazole / albendazole)	X		X
	BASIC AMENITIES *Availability of…*			
28	Power			X
29	Improved water source		X	X
30	Room with auditory and visual privacy	X	X	X
31	Sanitation facilities		X	X
32	Communication equipment			X
33	Computer with email/internet access			X
34	Emergency transportation			X
	HUMAN RESOURCES *Availability of…*			
35	Guidelines for ANC available at the facility	X		X
36	Proportion of health facility staff providing ANC services trained in ANC in the last two years	X	X	X
37	Proportion of health facility staff providing ANC services who received supervision in the last six months	X		X
38	Proportion of health facility staff providing ANC services who report availability of opportunities for promotion in their current job			X

For peer review only - http://bmjopen.bmj.com/site/about/guidelines.xhtml

Supplementary Table 3: List of Items Included in the Provision of Care Indices, by Domain

#	Item Name	Corresponding Readiness Domain
	HISTORY-TAKING AND CLIENT EDUCATION/COUNSELLING *During ANC visit the provider asks about/counsels the client on…*	
1	Personal history: client age	Human Resources
2	Personal history: medications client is taking	Human Resources
3	Personal history: date last menstrual period began	Human Resources
4	Personal history: any prior pregnancy	Human Resources
5	Past medical history for prior pregnancies: still birth	Human Resources
6	Past medical history for prior pregnancies: infant died in the first week of life	Human Resources
7	Past medical history for prior pregnancies: heavy bleeding during or after delivery	Human Resources
8	Past medical history for prior pregnancies: previous assisted delivery	Human Resources
9	Past medical history for prior pregnancies: previous spontaneous abortion	Human Resources
10	Past medical history for prior pregnancies: multiple pregnancies	Human Resources
11	Past medical history for prior pregnancies: prolonged labor	Human Resources
12	Past medical history for prior pregnancies: pregnancy-induced hypertension	Human Resources
13	Past medical history for prior pregnancies: pregnancy related convulsions	Human Resources
14	Past medical history for prior pregnancies: high fever or infection during prior pregnancy	Human Resources
15	History of complaints in current pregnancy: vaginal bleeding	Human Resources
16	History of complaints in current pregnancy: fever	Human Resources
17	History of complaints in current pregnancy: headache or blurred vision	Human Resources
18	History of complaints in current pregnancy: swollen face or hands or extremities	Human Resources
19	History of complaints in current pregnancy: tiredness or breathlessness	Human Resources
20	History of complaints in current pregnancy: fetal movement (loss of, excessive, normal)	Human Resources
21	History of complaints in current pregnancy: cough or difficulty breathing for three weeks or longer	Human Resources
22	Client education/counselling: Process of pregnancy and its complications	Human Resources
23	Client education/counselling: Diet and nutrition	Human Resources
24	Client education/counselling: Danger signs in pregnancy	Human Resources

For peer review only - http://bmjopen.bmj.com/site/about/guidelines.xhtml

#	Item Name	Corresponding Readiness Domain
25	Client education/counselling: Voluntary counselling and testing for HIV	Human Resources
26	Client education/counselling: Breastfeeding	Human Resources
27	Client education/counselling: Plans of delivery (emergency preparedness, place of delivery, transportation, financial arrangements)	Human Resources
	EXAMINATION *During ANC visit the provider examines the client for…*	
28	Oedema	Human Resources
29	Blood pressure	Human Resources, Equipment
30	Weight	Human Resources, Equipment
31	Palpating the client's abdomen for fundal height	Human Resources, Equipment
	DIAGNOSTICS *During ANC visit the provider performs or refers the client to test for…*	
32	Hemoglobin	Human Resources, Diagnostics
33	Grouping and rhesus factor	Human Resources, Diagnostics
34	Syphilis / RPR	Human Resources, Diagnostics
35	HIV	Human Resources, Diagnostics
36	Urine – protein, glucose	Human Resources, Diagnostics
	PREVENTATIVE TREATMENT *During ANC visit the provider administers or prescribes…*	
37	Iron and/or folic acid	Human Resources, Medicines
38	Tetanus toxoid	Human Resources, Medicines
	CLIENT EXPERIENCE *During ANC visit…*	
39	Client is able to discuss problems or concerns with provider	Human Resources
40	Client satisfied with the amount of explanation received about the problem or treatment	Human Resources

For peer review only - http://bmjopen.bmj.com/site/about/guidelines.xhtml

#	Item Name	Corresponding Readiness Domain
41	Client satisfied with how the staff treated them	Human Resources
42	Privacy from having others see the consultation	Human Resources, Basic Amenities
43	Privacy from having others hear the consultation	Human Resources, Basic Amenities
44	Client satisfied with the wait time	Human Resources
45	Client satisfied with the number of days services are available at the facility	Human Resources
46	Client satisfied with the hours of service at the facility	Human Resources
47	Client satisfied with the cost for services or treatments	Human Resources
48	Client satisfied with the availability of medicines at the facility	Human Resources, Medicines
49	Client satisfied with the cleanliness of the facility	Human Resources

For peer review only

Supplementary Table 4: Expert Survey Results (mean, sd, min, max, n)

Item Name	Mean	Standard Deviation	Min.	Max.	N
EQUIPMENT AND SUPPLIES *Availability of…*					
Blood pressure apparatus	3.933	0.258	3	4	15
Examination light	2.600	0.828	1	4	15
Fetal stethoscope	3.733	0.594	2	4	15
Stethoscope	3.467	0.834	2	4	15
Adult weighing scale	3.000	0.845	2	4	15
Thermometer	3.533	0.834	1	4	15
Tape measure for fundal height	3.333	0.724	2	4	15
Height board	2.692	0.947	1	4	13
Speculum	3.429	0.646	2	4	14
Examination bed	3.429	0.938	1	4	14
Latex gloves	3.600	0.632	2	4	15
Single use syringes	3.643	0.745	2	4	14
Soap and water OR alcohol-based hand rub	3.733	0.704	2	4	15
Disinfectant	3.533	0.640	2	4	15
Appropriate storage of sharps waste (sharps box)	3.400	0.910	1	4	15
Appropriate storage of infectious waste (pedal bin with lid and liner)	3.333	0.976	1	4	15
Safe final disposal of sharps (incineration)	3.200	0.941	1	4	15
Safe final disposal of infectious wastes (incineration)	3.133	0.990	1	4	15
Medical masks	2.200	0.775	1	4	15
Gowns	2.000	0.845	1	4	15
Eye protection	2.067	0.884	1	4	15
Gum boots	1.667	0.724	1	3	15
DIAGNOSTICS *Availability on-site to test for…*					
Hemoglobin	3.800	0.414	3	4	15
Urine dipstick- protein	3.733	0.458	3	4	15

For peer review only - http://bmjopen.bmj.com/site/about/guidelines.xhtml

Item Name	Mean	Standard Deviation	Min.	Max.	N
Urine dipstick- glucose	3.467	0.743	2	4	15
Grouping and Rhesus factor	3.357	0.745	2	4	14
Syphilis RDT / RPR	3.667	0.488	3	4	15
HIV testing / RDT	3.667	0.488	3	4	15
MEDICINES AND COMMODITIES *Availability on-site of...*					
Iron tablets	3.800	0.414	3	4	15
Folic acid tablets	3.667	0.617	2	4	15
Tetanus toxoid vaccine	3.800	0.414	3	4	15
Intermittent Preventative Treatment (IPT) drug	3.667	0.488	3	4	15
Insecticide treated nets (ITNs) or vouchers	3.267	0.799	2	4	15
De-worming drugs (mebendazole / albendazole)	3.000	0.784	2	4	14
BASIC AMENITIES *Availability of...*					
Power	3.400	0.737	2	4	15
Improved water source	3.667	0.617	2	4	15
Room with auditory and visual privacy	3.533	0.640	2	4	15
Sanitation facilities	3.600	0.507	3	4	15
Communication equipment	3.267	0.884	1	4	15
Computer with email/internet access	2.267	1.033	1	4	15
Emergency transportation	3.400	0.986	1	4	15
HUMAN RESOURCES *Availability of...*					
Guidelines for ANC available at the facility	3.267	0.884	1	4	15
Proportion of health facility staff providing ANC services trained in ANC in the last two years	3.400	0.737	2	4	15
Proportion of health facility staff providing ANC services who received supervision in the last six months	3.267	0.799	2	4	15

For peer review only

Item Name	Mean	Standard Deviation	Min.	Max.	N
Proportion of health facility staff providing ANC services who report availability of opportunities for promotion in their current job	2.500	0.760	2	4	14
HISTORY-TAKING *During ANC visit the provider asks about…*					
Personal history: client age	3.533	0.640	2	4	15
Personal history: medications client is taking	3.733	0.458	3	4	15
Personal history: date last menstrual period began	3.933	0.258	3	4	15
Personal history: any prior pregnancy	3.867	0.352	3	4	15
Family history	3.200	0.862	2	4	15
Social history	3.067	0.704	2	4	15
Past medical history for prior pregnancies: still birth	3.867	0.352	3	4	15
Past medical history for prior pregnancies: infant died in the first week of life	3.600	0.632	2	4	15
Past medical history for prior pregnancies: heavy bleeding during or after delivery	3.733	0.458	3	4	15
Past medical history for prior pregnancies: previous assisted delivery	3.733	0.458	3	4	15
Past medical history for prior pregnancies: previous spontaneous abortion	3.467	0.834	2	4	15
Past medical history for prior pregnancies: multiple pregnancies	3.600	0.737	2	4	15
Past medical history for prior pregnancies: prolonged labor	3.600	0.632	2	4	15
Past medical history for prior pregnancies: pregnancy-induced hypertension	4.000	0.000	4	4	15
Past medical history for prior pregnancies: pregnancy related convulsions	4.000	0.000	4	4	15

For peer review only

Item Name	Mean	Standard Deviation	Min.	Max.	N
Past medical history for prior pregnancies: high fever or infection during prior pregnancy	3.533	0.640	2	4	15
History of complaints in current pregnancy: vaginal bleeding	4.000	0.000	4	4	15
History of complaints in current pregnancy: fever	3.867	0.352	3	4	15
History of complaints in current pregnancy: headache or blurred vision	4.000	0.000	4	4	15
History of complaints in current pregnancy: swollen face or hands or extremities	4.000	0.000	4	4	15
History of complaints in current pregnancy: tiredness or breathlessness	3.667	0.816	1	4	15
History of complaints in current pregnancy: fetal movement (loss of, excessive, normal)	3.933	0.258	3	4	15
History of complaints in current pregnancy: cough or difficulty breathing for three weeks or longer	3.733	0.458	3	4	15
History of complaints in current pregnancy: amniotic leakage	3.786	0.579	2	4	14
EXAMINATION *During ANC visit the provider examines the client for…*					
Head to toe (whole body)	2.600	1.121	1	4	15
Pallor	3.400	0.632	2	4	15
Oedema	3.733	0.458	3	4	15
Breast	2.533	0.990	1	4	15
Lungs and heart	2.600	1.056	1	4	15
OBSERVATION AND CLINICAL INVESTIGATION *During ANC visit the provider assesses the client's…*					
Blood pressure	4.000	0.000	4	4	15
Weight	3.133	0.834	2	4	15
Temperature	3.333	0.900	1	4	15
Pulse	3.357	0.929	1	4	14

For peer review only - http://bmjopen.bmj.com/site/about/guidelines.xhtml

Item Name	Mean	Standard Deviation	Min.	Max.	N
OBSTETRIC COMPLICATIONS<br>*During ANC visit the provider assesses the client for potential obstetric complications by…*					
Palpating the client's abdomen for fundal height	3.667	0.617	2	4	15
Palpating the client's abdomen for fetal presentation	3.600	0.632	2	4	15
Listening to the client's abdomen for fetal heartbeat	3.800	0.561	2	4	15
Conducting an ultrasound/ referring client for ultrasound/ look at recent ultrasound report	2.600	0.986	1	4	15
PELVIC EXAMINATION<br>*During ANC visit the provider conducts a pelvic exam with…*					
Soft tissue assessment/ Vaginal examination	2.154	1.214	1	4	13
Bony pelvic assessment	2.571	1.342	1	4	14
LABORATORY INVESTIGATIONS<br>*During ANC visit the provider performs or refers the client to test for…*					
Hemoglobin	3.733	0.458	3	4	15
Grouping and rhesus factor	3.357	0.633	2	4	14
Syphilis / RPR	3.667	0.488	3	4	15
HIV	3.867	0.352	3	4	15
Urine – protein	3.667	0.617	2	4	15
Urine – glucose	3.400	0.828	2	4	15
Urine – acetone	2.500	1.087	1	4	12
DRUG ADMINISTRATION AND IMMUNIZATION<br>*During ANC visit the provider administers or prescribes…*					
Iron and/or folic acid	3.800	0.414	3	4	15
Antimalarials	3.429	0.756	2	4	14
Tetanus toxoid	3.600	0.737	2	4	15
CLIENT EDUCATION AND COUNSELLING<br>*During ANC visit the provider counsels the client on…*					
Process of pregnancy and its complications	3.733	0.594	2	4	15
Diet and nutrition	3.600	0.632	2	4	15

For peer review only - http://bmjopen.bmj.com/site/about/guidelines.xhtml

Item Name	Mean	Standard Deviation	Min.	Max.	N
Rest and exercise in pregnancy	2.933	0.704	2	4	15
Personal hygiene	2.867	0.990	1	4	15
Danger signs in pregnancy	3.933	0.258	3	4	15
Use of drugs in pregnancy	3.400	0.828	2	4	15
Effects of STI/HIV/AIDS	3.467	0.834	2	4	15
Voluntary counselling and testing for HIV	3.667	0.617	2	4	15
Care of breasts	2.867	0.743	2	4	15
Breastfeeding	3.867	0.352	3	4	15
Symptoms/signs of labor	3.867	0.516	2	4	15
Plans of delivery (emergency preparedness, place of delivery, transportation, financial arrangements)	3.733	0.458	3	4	15
Plans for postpartum care	3.400	0.632	2	4	15
Family planning	3.533	0.640	2	4	15
Harmful habits (e.g. smoking, drug abuse, alcoholism)	3.533	0.640	2	4	15
Schedule of return visit	3.867	0.352	3	4	15
CLIENT EXPERIENCE *During ANC visit...*					
Client is able to discuss problems or concerns with provider	3.867	0.352	3	4	15
Client satisfied with the amount of explanation received about the problem or treatment	3.600	0.632	2	4	15
Client satisfied with how the staff treated them	3.667	0.617	2	4	15
Privacy from having others see the consultation	3.533	0.640	2	4	15
Privacy from having others hear the consultation	3.533	0.640	2	4	15
Client satisfied with the wait time	3.133	0.834	2	4	15
Client satisfied with the number of days services are available at the facility	3.000	0.756	2	4	15

For peer review only

Item Name	Mean	Standard Deviation	Min.	Max.	N
Client satisfied with the hours of service at the facility	3.067	0.704	2	4	15
Client satisfied with the cost for services or treatments	3.333	0.617	2	4	15
Client satisfied with the availability of medicines at the facility	3.467	0.516	3	4	15
Client satisfied with the cleanliness of the facility	3.533	0.516	3	4	15

For peer review only

Expert Survey: Antenatal Care Service Quality

INTRODUCTION:
We invite you to participate in a brief online survey as part of a study aiming to assess the relationship between structural quality and process quality for antenatal care (ANC) services in low- and middle-income countries being conducted at the Johns Hopkins Bloomberg School of Public Health. You have been identified as an expert in maternal health with experience working in low- and middle-income countries and as such we would greatly appreciate your contribution to this work.

In order to explore the relationship between structural quality and process quality for ANC services, we first need to develop ANC quality of care metrics. While there are many definitions and frameworks for defining quality of care, there is a lack of consensus on indicators for measuring the quality of antenatal care. The goal of this survey is to elicit expert opinion on the items that are important to include in measures of ANC service quality. We expect that the results of this work will be published in a dissertation, and eventually a journal article.

The survey can be accessed at the following link:
https://tinyurl.com/ExpertSurvey-ANC

The survey should take approximately 20-30 minutes to complete. We would greatly appreciate if you can respond to the survey by 10 September 2018. If you have any questions, please feel free to contact Ashley Sheffel (asheffel@jhu.edu) or Melinda Munos (mmunos@jhu.edu).

Many thanks in advance for your time and contribution.

CONSENT:
By checking "Yes" I agree to participate in the survey. I understand the purpose and nature of this activity and I am participating voluntarily. I understand that I can stop taking the survey at any time, without any penalty or consequences. I understand that any personal information collected through this survey will remain confidential.

YES □ → continue NO □ → end survey

BACKGROUND:
In order to create a comprehensive list of items related to ANC service quality, a review of the Service Availability and Readiness Assessment Indicators and the WHO Focused Antenatal Care Guidelines was conducted along with a review of the data available in the Service Provision Assessment questionnaire, which is the main data source for this research. The identified items have been organized according to the dimensions of quality of care as proposed by the WHO Quality of Care Framework for Maternal and Newborn Health: essential physical resources, competent and motivated human resources, provision of care, and experience of care. Brief descriptions of each dimension are provided below.

DIMENSION DESRIPTIONS:
Physical resources: This dimension includes items related to the availability of an appropriate physical environment, equipment, supplies, medicines, and diagnostics required to provide ANC services.

For peer review only - http://bmjopen.bmj.com/site/about/guidelines.xhtml

Human resources: This dimension includes items related to the availability of competent, motivated human resources to provide ANC services.

Provision of care: This dimension includes items related to use of evidence-based practices, information systems in which record- keeping allows review, and functioning systems for referral between different levels of care.

Experience of care: This dimension includes items related to effective communication with women and their families about the care provided, their expectations and their rights; care with respect and preservation of dignity; and access to the social and emotional support of their choice.

INSTRUCTIONS: For each dimension, a set of items has been identified that are related to delivery of ANC. The goal is to identify the items which are most important for delivery of a high-quality ANC service ***within each dimension***. For each item, please provide a rating of importance of the item to ANC service quality based on your expert opinion. Circle only one response option per item. If you are unfamiliar with an item or don't know how to rate the item, please select "Don't know".

RATINGS:
1 = Unimportant/Non-informative for measuring ANC service quality
2= Somewhat important for measuring ANC service quality
3 = Very important for measuring ANC service quality
4 = Essential for measuring ANC service quality
98 = Don't know

For peer review only

For peer review only - http://bmjopen.bmj.com/site/about/guidelines.xhtml

#	Item Name	Response options				
		Unimportant / Non-informative	Somewhat important	Very important	Essential	Don't Know
ESSENTIAL PHYSICAL RESOURCES						
EQUIPMENT AND SUPPLIES<br>*Availability of…*						
1	Blood pressure apparatus	1	2	3	4	98
2	Examination light	1	2	3	4	98
3	Fetal stethoscope	1	2	3	4	98
4	Stethoscope	1	2	3	4	98
5	Adult weighing scale	1	2	3	4	98
6	Thermometer	1	2	3	4	98
7	Tape measure for fundal height	1	2	3	4	98
8	Height board	1	2	3	4	98
9	Speculum	1	2	3	4	98
10	Examination bed	1	2	3	4	98
11	Latex gloves	1	2	3	4	98
12	Single use syringes	1	2	3	4	98
13	Soap and water OR alcohol based hand rub	1	2	3	4	98
14	Disinfectant	1	2	3	4	98
15	Appropriate storage of sharps waste (sharps box)	1	2	3	4	98
16	Appropriate storage of infectious waste (pedal bin with lid and liner)	1	2	3	4	98
17	Safe final disposal of sharps (incineration)	1	2	3	4	98
18	Safe final disposal of infectious wastes (incineration)	1	2	3	4	98
19	Medical masks	1	2	3	4	98
20	Gowns	1	2	3	4	98
21	Eye protection	1	2	3	4	98
22	Gum boots	1	2	3	4	98
DIAGNOSTICS<br>*Availability on-site to test for…*						
23	Hemoglobin	1	2	3	4	98
24	Urine dipstick- protein	1	2	3	4	98
25	Urine dipstick- glucose	1	2	3	4	98
26	Grouping and Rhesus factor	1	2	3	4	98
27	Syphilis RDT / RPR	1	2	3	4	98

For peer review only

For peer review only - http://bmjopen.bmj.com/site/about/guidelines.xhtml

#	Item Name	Response options				
		Unimportant / Non-informative	Somewhat important	Very important	Essential	Don't Know
28	HIV testing / RDT	1	2	3	4	98
MEDICINES AND COMMODITIES<br>*Availability on-site of…*						
29	Iron tablets	1	2	3	4	98
30	Folic acid tablets	1	2	3	4	98
31	Tetanus toxoid vaccine	1	2	3	4	98
32	Intermittent Preventative Treatment (IPT) drug	1	2	3	4	98
33	Insecticide treated nets (ITNs) or vouchers	1	2	3	4	98
34	De-worming drugs (mebendazole / albendazole)	1	2	3	4	98
BASIC AMENITIES<br>*Availability of…*						
35	Power	1	2	3	4	98
36	Improved water source	1	2	3	4	98
37	Room with auditory and visual privacy	1	2	3	4	98
38	Sanitation facilities	1	2	3	4	98
39	Communication equipment	1	2	3	4	98
40	Computer with email/internet access	1	2	3	4	98
41	Emergency transportation	1	2	3	4	98
COMPETENT, MOTIVATED HUMAN RESOURCES						
42	Guidelines for ANC available at the facility	1	2	3	4	98
43	At least one health facility staff member trained in ANC in the last two years	1	2	3	4	98
44	At least 75% of staff offering ANC at the facility have received supervision in the last six months	1	2	3	4	98
45	At least 75% of staff offering ANC at the facility report availability of opportunities for promotion in their current job	1	2	3	4	98
PROVISION OF CARE						
HISTORY TAKING<br>*During ANC visit the provider asks about…*						
46	Personal history: client age	1	2	3	4	98
47	Personal history: medications client is taking	1	2	3	4	98
48	Personal history: date last menstrual period began	1	2	3	4	98
49	Personal history: any prior pregnancy	1	2	3	4	98
50	Family history	1	2	3	4	98

For peer review only - http://bmjopen.bmj.com/site/about/guidelines.xhtml

#	Item Name	Response options				
		Unimportant / Non-informative	Somewhat important	Very important	Essential	Don't Know
51	Social history	1	2	3	4	98
52	Past medical history for prior pregnancies: still birth	1	2	3	4	98
53	Past medical history for prior pregnancies: infant died in the first week of life	1	2	3	4	98
54	Past medical history for prior pregnancies: heavy bleeding during or after delivery	1	2	3	4	98
55	Past medical history for prior pregnancies: previous assisted delivery	1	2	3	4	98
56	Past medical history for prior pregnancies: previous spontaneous abortion	1	2	3	4	98
57	Past medical history for prior pregnancies: multiple pregnancies	1	2	3	4	98
58	Past medical history for prior pregnancies: prolonged labor	1	2	3	4	98
59	Past medical history for prior pregnancies: pregnancy-induced hypertension	1	2	3	4	98
60	Past medical history for prior pregnancies: pregnancy related convulsions	1	2	3	4	98
61	Past medical history for prior pregnancies: high fever or infection during prior pregnancy	1	2	3	4	98
62	History of complaints in current pregnancy: vaginal bleeding	1	2	3	4	98
63	History of complaints in current pregnancy: fever	1	2	3	4	98
64	History of complaints in current pregnancy: headache or blurred vision	1	2	3	4	98
65	History of complaints in current pregnancy: swollen face or hands or extremities	1	2	3	4	98
66	History of complaints in current pregnancy: tiredness or breathlessness	1	2	3	4	98
67	History of complaints in current pregnancy: fetal movement (loss of, excessive, normal)	1	2	3	4	98
68	History of complaints in current pregnancy: cough or difficulty breathing for three weeks or longer	1	2	3	4	98
69	History of complaints in current pregnancy: amniotic leakage	1	2	3	4	98
EXAMINATION<br>*During ANC visit the provider examines the client for…*						
70	Head to toe (whole body)	1	2	3	4	98
71	Pallor	1	2	3	4	98
72	Oedema	1	2	3	4	98
73	Breast	1	2	3	4	98
74	Lungs and heart	1	2	3	4	98
OBSERVATION AND CLINICAL INVESTIGATION<br>*During ANC visit the provider assesses the client's…*						

For peer review only - http://bmjopen.bmj.com/site/about/guidelines.xhtml

#	Item Name	Response options				
		Unimportant / Non-informative	Somewhat important	Very important	Essential	Don't Know
75	Blood pressure	1	2	3	4	98
76	Weight	1	2	3	4	98
77	Temperature	1	2	3	4	98
78	Pulse	1	2	3	4	98
OBSTETRIC COMPLICATIONS<br>*During ANC visit the provider assesses the client for potential obstetric complications by…*						
79	Palpating the client's abdomen for fundal height	1	2	3	4	98
80	Palpating the client's abdomen for fetal presentation	1	2	3	4	98
81	Listening to the client's abdomen for fetal heartbeat	1	2	3	4	98
82	Conducting an ultrasound/ referring client for ultrasound/ look at recent ultrasound report	1	2	3	4	98
PELVIC EXAMINATION<br>*During ANC visit the provider conducts a pelvic exam with…*						
83	Soft tissue assessment/ Vaginal examination	1	2	3	4	98
84	Bony pelvic assessment	1	2	3	4	98
LABORATORY INVESTIGATIONS<br>*During ANC visit the provider performs or refers the client to test for…*						
85	Hemoglobin	1	2	3	4	98
86	Grouping and rhesus factor	1	2	3	4	98
87	Syphilis / RPR	1	2	3	4	98
88	HIV	1	2	3	4	98
89	Urine – protein	1	2	3	4	98
90	Urine -sugar	1	2	3	4	98
91	Urine- acetone	1	2	3	4	98
DRUG ADMINISTRATION AND IMMUNIZATION<br>*During ANC visit the provider administers or prescribes…*						
92	Iron and/or folic acid	1	2	3	4	98
93	Antimalarials	1	2	3	4	98
94	Tetanus toxoid	1	2	3	4	98
CLIENT EDUCATION AND COUNSELING<br>*During ANC visit the provider counsels the client on…*						
95	Process of pregnancy and its complications	1	2	3	4	98
96	Diet and nutrition	1	2	3	4	98
97	Rest and exercise in pregnancy	1	2	3	4	98
98	Personal hygiene	1	2	3	4	98

For peer review only - http://bmjopen.bmj.com/site/about/guidelines.xhtml

#	Item Name	Response options				
		Unimportant / Non-informative	Somewhat important	Very important	Essential	Don't Know
99	Danger signs in pregnancy	1	2	3	4	98
100	Use of drugs in pregnancy	1	2	3	4	98
101	Effects of STI/HIV/AIDS	1	2	3	4	98
102	Voluntary counselling and testing for HIV	1	2	3	4	98
103	Care of breasts	1	2	3	4	98
104	Breastfeeding	1	2	3	4	98
105	Symptoms/signs of labor	1	2	3	4	98
106	Plans of delivery (emergency preparedness, place of delivery, transportation, financial arrangements)	1	2	3	4	98
107	Plans for postpartum care	1	2	3	4	98
108	Family planning	1	2	3	4	98
109	Harmful habits (e.g. smoking, drug abuse, alcoholism)	1	2	3	4	98
110	Schedule of return visit	1	2	3	4	98
EXPERIENCE OF CARE						
111	Client is able to discuss problems or concerns with provider	1	2	3	4	98
112	Client satisfied with the amount of explanation received about the problem or treatment	1	2	3	4	98
113	Client satisfied with how the staff treated them	1	2	3	4	98
114	Privacy from having others see the consultation	1	2	3	4	98
115	Privacy from having others hear the consultation	1	2	3	4	98
116	Client satisfied with the wait time	1	2	3	4	98
117	Client satisfied with the number of days services are available at the facility	1	2	3	4	98
118	Client satisfied with the hours of service at the facility	1	2	3	4	98
119	Client satisfied with the cost for services or treatments	1	2	3	4	98
120	Client satisfied with the availability of medicines at the facility	1	2	3	4	98
121	Client satisfied with the cleanliness of the facility	1	2	3	4	98

Are there any items you feel are essential for delivering a high-quality ANC service that do not appear in this questionnaire?

____

____

For peer review only - http://bmjopen.bmj.com/site/about/guidelines.xhtml

____
____
____

RESPONDENT INFORMATION:

Please answer the following question about yourself and your current role. This information will only be used for internal purposes and will not be disseminated.

1. Name: ___________________________
2. Email address: ___________________________
3. Organization: ___________________________
4. Position title: ___________________________
5. Years of experience in maternal health: ___________________________

The survey is now complete. Thank you for helping us with our research. We greatly appreciate your time and insights.

For peer review only - http://bmjopen.bmj.com/site/about/guidelines.xhtml

BMJ Open

Development of Summary Indices of Antenatal Care Service Quality in Haiti, Malawi, and Tanzania

Journal:	*BMJ Open*
Manuscript ID	bmjopen-2019-032558.R2
Article Type:	Research
Date Submitted by the Author:	24-Oct-2019

Complete List of Authors:	Sheffel, Ashley; Johns Hopkins University Bloomberg School of Public Health, Department of International Health<br>Zeger, Scott; Johns Hopkins University Bloomberg School of Public Health, Department of Biostatistics; Johns Hopkins University Bloomberg School of Public Health, Department of International Health<br>Heidkamp, Rebecca; Johns Hopkins University Bloomberg School of Public Health, International Health<br>Munos, Melinda; Johns Hopkins University Bloomberg School of Public Health, Department of International Health

**Primary Subject Heading**:	Public health
Secondary Subject Heading:	Global health, Health services research
Keywords:	Maternal medicine < OBSTETRICS, International health services < HEALTH SERVICES ADMINISTRATION & MANAGEMENT, Quality in health care < HEALTH SERVICES ADMINISTRATION & MANAGEMENT, PUBLIC HEALTH, STATISTICS & RESEARCH METHODS

SCHOLARONE™ Manuscripts

Title: Development of Summary Indices of Antenatal Care Service Quality in Haiti, Malawi, and Tanzania

Authors: Ashley Sheffel[1], Scott Zeger[1,2], Rebecca Heidkamp[1], Melinda Munos[1]

Affiliations:

[1]Department of International Health, Johns Hopkins Bloomberg School of Public Health, Baltimore, MD, USA

[2]Department of Biostatistics, Johns Hopkins Bloomberg School of Public Health, Baltimore, MD, USA

Author contributions: AS, MM, RH, and SZ contributed to conceptualizing the paper and analysis; AS prepared the manuscript; MM, RH, and SZ critically reviewed and revised the manuscript.

Corresponding author:

Ashley Sheffel

Johns Hopkins Bloomberg School of Public Health

615 N Wolfe St. Room E8009

Baltimore, MD 21205 USA

asheffel@jhu.edu

Keywords: Maternal medicine, International health services, Quality in health care, Public health, Statistics & research methods

Word count: 5,575

Reference count: 54

For peer review only - http://bmjopen.bmj.com/site/about/guidelines.xhtml

Table/Figure count: 5 tables, 3 figures

Funding: This work was supported by the Improving Measurement and Program Design grant (OPP1172551) from the Bill & Melinda Gates Foundation.

Abbreviations:

ANC – Antenatal Care

CoV – Coefficient of Variation

DHS – Demographic and Health Survey

FANC – Focused Antenatal Care

IQR – Inter-Quartile Range

LMIC – Low-Income and Middle- Income Countries

PCA – Principal Components Analysis

SPA – Service Provision Assessment

USAID – US Agency for International Development

For peer review only

ABSTRACT

Introduction: Measuring quality of care in low-income and middle-income countries is complicated by the lack of a standard, universally-accepted definition for "quality" for any particular service, as well as limited guidance on which indicators to include in measures of quality of care, and how to incorporate those indicators into summary indices. The aim of this paper is to develop, characterize, and compare a set of antenatal care (ANC) indices for facility readiness and provision of care.

Methods: We created nine indices for facility readiness using three methods for selecting items and three methods for combining items. In addition, we created three indices for provision of care using one method for selecting items and three methods for combining items. For each index, we calculated descriptive statistics, categorized the continuous index scores using tercile cut points to assess comparability of facility classification, and examined the variability and distribution of scores.

Results: Our results showed that within a country the indices were quite similar in terms of mean index score, facility classification, coefficient of variation, floor and ceiling effects, and the inclusion of items in an index with a range of variability. Notably, the indices created using PCA to combine the items were the most different from the other

For peer review only

For peer review only - http://bmjopen.bmj.com/site/about/guidelines.xhtml

indices. In addition, the index created by taking a weighted average of a core set of items had lower agreement with the other indices when looking at facility classification.

Conclusions: As improving quality of care becomes integral to global efforts to produce better health outcomes, demand for guidance on creating standardized measures of service quality will grow. This study provides health systems researchers with a comparison of methodologies commonly used to create summary indices of ANC service quality and it highlights the similarities and differences between methods.

For peer review only

For peer review only - http://bmjopen.bmj.com/site/about/guidelines.xhtml

STRENGTHS AND LIMITATIONS OF THIS STUDY

- To our knowledge, this is the first study that uses multiple methodologies to develop and compare indices for ANC facility readiness and provision of care.
- Our approaches to item selection and combination were based on the most commonly used approaches in the literature and we included those most likely to be useful to LMICs.
- The analysis was conducted using data from multiple countries which strengthens our ability to generalize the findings to other similar contexts.
- The SPA survey, while quite comprehensive, does not capture every aspect of quality of care that may be important to include in quality of care metrics. This analysis does not provide a formal validation of any one ANC quality of care index.

For peer review only

For peer review only - http://bmjopen.bmj.com/site/about/guidelines.xhtml

INTRODUCTION

Reducing maternal morbidity and mortality has been a global priority over the last several decades. While progress has been made in both improving coverage and reducing mortality, there remains an unacceptably high number of deaths of women globally [1]. In many countries, considerable increases in the coverage of health services have not translated into sufficient reductions in mortality [2]. This finding suggests that quality of care may play a critical role in producing better health outcomes. Reducing maternal mortality will require not only increasing the coverage of health interventions, but also ensuring that those interventions are delivered with a high level of quality [3 4]. These interventions include not only intrapartum and postpartum care, but also antenatal care (ANC), which is important to maintaining a healthy pregnancy [5-7], and promotes safe delivery, postnatal attendance, and is positively associated with an increase in facility-based deliveries [8-10].

Low-income and middle-income countries (LMICs) are increasingly shifting focus towards making improvements in the quality of health services delivered, which requires measuring quality of care and monitoring progress. However, for LMICs to systematically and comprehensively measure quality of care, they require a definition of quality of care and clear, specific indicators to operationalize the definition. Many quality of care frameworks exist, most of which build from Donabedian's 1988 framework characterizing quality at three levels: structure, commonly called facility readiness (the

For peer review only - http://bmjopen.bmj.com/site/about/guidelines.xhtml

setting in which care occurs including material resources, human resources, and organizational structure), process (commonly called provision of care — the quality of medical advice delivered by providers to clients, as well as interpersonal relationships between the provider and the client), and outcome (the effects of care on the health status and behaviors of patients, as well as improvements in patient knowledge and the degree of satisfaction with care) [11 12]. While there may be growing consensus on the core components of a framework for describing quality of care, measuring quality of care is complicated by lack of a standard, universally accepted definition for "quality" for any one particular service [13-15]. In addition, there is limited guidance on which indicators to include in measures of quality of care and how to incorporate those indicators into summary indices.

Studies on quality of care in LMICs largely rely on data from health facility surveys that collect data on facility readiness and provision of care. These studies often use summary indices or composite scores to provide an overall description of service quality[16-26]. Summary scores are useful to LMIC governments in that they simplify complex data and enable comparison of performance within facilities, across administrative units, and over time. However, there is little consistency between studies in terms of the items included in summary metrics of quality of care, or in how the metrics are created. A scoping review published in 2018 identified numerous studies which utilized health facility survey data to assess maternal and newborn quality of care

For peer review only

For peer review only - http://bmjopen.bmj.com/site/about/guidelines.xhtml

in LMICs and found that studies used various approaches to create quality of care metrics [27]. ***Supplementary Table 1*** illustrates this with an excerpt of studies that used data from the Service Provision Assessment (SPA) to create quality of care metrics for ANC specifically, each using a different approach. Item selection was guided by different sources including the Donabedian quality of care framework (for example [17 28]), clinical guidelines (for example [18 19 29 30]), and various definitions of quality of care (for example [20 21]). The number of items that were included in an index varied from a minimum of seven to a maximum of forty, and various methods were used to combine items into indices. These methods include a simple and weighted additive approaches (for example [20 22-26]) as well as principal components analysis (PCA) (for example [28 31-33]). While many of these studies had some overlap in the items selected for inclusion in their ANC quality of care index, the quality of care indices varied greatly in the items selected for inclusion in the indices and in the methodology utilized for combining items into a summary measure.

As quality of care indices are increasingly used by countries, global agencies, and researchers to quantify the quality of health services and estimate effective coverage, there is a need to standardize the methodology for creating these measures. To our knowledge, no studies have compared the methodologies used to create indices of ANC service quality. The objective of this paper is to develop, characterize, and compare a set of facility readiness and provision of care indices for ANC.

For peer review only - http://bmjopen.bmj.com/site/about/guidelines.xhtml

METHODS

Overall approach

Using data from an expert survey, we created nine facility readiness indices using three methods for selecting items (a "core set" of items, "expert survey" set of items, and "maximum" set of items) and three methods for combining items (simple additive, weighted additive, and PCA) (***Figure 1***). We created three indices for provision of care using one method for selecting items (“expert survey” set of items) and three methods for combining items (simple additive, weighted additive, and PCA) (***Figure 1***). Data for these indices comes from the Service Provision assessment (SPA). We then compared the indices and examined the variability and distribution of the index scores.

Data Source

The Service Provision Assessment (SPA) is a health facility assessment used in LMICs to generate nationally representative data on health service delivery [34]. While there are a number of widely used health facility assessment tools, we chose to use SPA data for this analysis as the SPA has been conducted in a number of different countries, includes observation of clinical care, and the data is publicly available through the Demographic and Health Survey (DHS) program. The SPA includes a standard set of survey instruments: a facility inventory questionnaire, health worker interviews, observation of ANC consultations, and exit interviews with ANC clients.

For peer review only - http://bmjopen.bmj.com/site/about/guidelines.xhtml

We examined all SPA surveys for inclusion in the analysis (total of 16). We included all SPA surveys which utilized the DHS-VI or DHS-VII questionnaire (four surveys excluded), that included observations of ANC consultations (three surveys excluded), were conducted in the last five years (between 2013 and 2018) (two surveys excluded), and for which recode data files were available (four surveys excluded). The included surveys are from Haiti (2013), Malawi (2013/14), and Tanzania (2014/15). The SPA final country reports contain comprehensive information on the survey methodology and questionnaires [35-37]. Briefly described, the survey in Tanzania was a nationally representative sample of health facilities. Health facilities were selected using stratified systematic probability sampling with stratification by region and facility type (with oversampling of some facility types such as hospitals). In Haiti and Malawi, the survey was a census of all health facilities in the country. In all three countries, all surveyed facilities completed the facility inventory module. In addition, within each facility, up to eight health workers were interviewed including all health workers whose consultations were observed and those who provided information for any section of the facility questionnaire. ANC clients were selected for observation using systematic sampling, based on the number of clients present at each service site on the day of the visit. At facilities where the number of ANC clients expected on the day of the survey could not be predetermined, the sample was opportunistic since clients were selected as they arrived. Observation was completed for a minimum of five clients per service

For peer review only - http://bmjopen.bmj.com/site/about/guidelines.xhtml

provider, with a maximum of 15 observations in any given facility for each service. Client exit interviews were conducted with every client whose visit was observed.

Item selection

To identify elements of facility readiness and the provision of ANC considered by experts to be the most important to ANC service quality, we reviewed the SARA indicators, WHO Focused Antenatal Care (FANC) guidelines, and WHO recommendations on antenatal care for a positive pregnancy experience, as well as the SPA questionnaire. This process identified a total of 121 items organized according to the dimensions of quality of care proposed by the WHO Quality of Care Framework for maternal and newborn health: essential physical resources (41 items), competent and motivated human resources (four items), provision of care (65 items), and experience of care (11 items) [38].

We then conducted a survey of 50 maternal health experts who had experience working in LMICs (questionnaire available as a ***Supplementary File***). Respondents were asked to rate each item based on overall importance. Importance ratings ranged from one (item was unimportant) to four (item was essential). Experts also provided a list of items they felt were important for delivering high-quality ANC services, but were missing from the survey instrument. Fifteen maternal health experts completed the survey, with respondents representing academic institutions, donor agencies, United

For peer review only - http://bmjopen.bmj.com/site/about/guidelines.xhtml

Nations agencies, global health implementing organizations, and Ministries of Health in LMICs.

Items that were not collected across SPA surveys in all three countries and items that are not required for a first ANC visit were excluded from all indices. For each readiness item, a binary score was created based on whether the criteria for availability were met on the day of the survey (1=available, 0=not available). For human resources items, each item is a proportion ranging from 0 to 1. For provision of care items, a binary score was created based on whether or not the activity was conducted during the ANC visit (1=conducted, 0=not conducted) or whether or not the client had a problem with the item during the ANC visit (1=no problem, 0=major or minor problem). The list of items included in each readiness and provision of care index can be found in ***Supplementary Table 2 and Supplementary Table 3***, respectively.

Three methods were used to select items to include in the facility readiness indices. The first method identified the **core set** of readiness items required to deliver ANC services. This set of items was identified by reviewing the provision of care items required for an ANC visit based on the WHO FANC guidelines and the WHO recommendations on antenatal care for a positive pregnancy experience, and by determining the human resources, equipment and supplies, medicines, and diagnostics required to deliver each specific item. In creating the core set of items for facility readiness, we mapped each provision of care item to the facility readiness items

For peer review only - http://bmjopen.bmj.com/site/about/guidelines.xhtml

required to deliver the specific service component. We found that of the 49 provision of care items, 36 items required only human resources and 13 items required human resources plus equipment, diagnostics, medicines, or basic amenities. The core index did not include standard precautions for infection prevention items because these are not explicitly required for any one provision of care item. A total of 21 items were selected for the core set readiness index.

The second method utilized results from the **expert survey** to identify the set of readiness items maternal health experts identified as essential to deliver ANC services. Mean ratings were calculated for each readiness item (see ***Supplementary Table 4*** for results). Items rated by the expert group with a mean importance of >3.4 out of 4 were selected for the expert survey index. The threshold for inclusion was determined by examining the distribution of scores and identifying a natural break point which separated the top-rated items from the rest. In addition, items were selected so that at least one item per domain (human resources, equipment and supplies, diagnostics, medicines, basic amenities) was included in the index. A total of 19 items were selected, representing 42% of the total items for the expert survey readiness index.

The third method identified the **maximum set** of readiness items used to deliver ANC services. This index includes all items identified in the SPA related to ANC readiness across the following domains: human resources, equipment and supplies, medicines, diagnostics, and basic amenities. Out of the 45 facility readiness items identified for

For peer review only - http://bmjopen.bmj.com/site/about/guidelines.xhtml

inclusion in the expert survey, a total of 38 items were selected for the maximum set readiness index. Seven items from the expert survey were not included in the maximum set of readiness items as data was not collected in the SPA on these items.

We utilized a single method to select items to include in the provision of care index. The method utilized results from the **expert survey** to select the set of provision of care items maternal health experts identified as essential to deliver ANC services. We chose this method because maternal health experts were the best source for determining which processes are essential to high quality ANC consultations. In addition, the experts selected most items as very important or essential, and therefore it was not appropriate to define a core and maximum set of items. Mean ratings were calculated for each provision of care item (see ***Supplementary Table 4*** for results). Items rated by the expert group with a mean importance of ≥3.0 out of 4 were selected for the provision of care index. The threshold for inclusion was decided by examining the distribution of scores which showed a bimodal distribution; then, selecting all items in the first mode. The expert survey respondents mentioned a number of provision of care items, largely related to experience of care, that they felt were essential for high quality care, but were missing from our survey and from SPA datasets. A total of 49 items were selected representing 64% of the total items for the provision of care index.

Item Combination

For peer review only - http://bmjopen.bmj.com/site/about/guidelines.xhtml

Three methods were used to combine items to create the facility readiness and provision of care indices — simple additive, weighted additive, and principal components analysis (PCA).

Simple additive: The simple additive index score was calculated by taking a sum of the items available divided by the total number of items in the index. We transformed the index into a score out of 100 by dividing by the number of items and multiplying by 100.The simple additive index weighted all items in the index equally.

Weighted additive: The weighted additive index was also calculated as a sum of items, but instead of assuming equal weights for all items, the weighted additive index accounted for the number of items within each domain. Readiness items were first grouped into five domains: human resources, equipment and supplies, medicines, diagnostics, and basic amenities. For the provision of care index, items were initially grouped into five domains: history and counselling, examination, diagnostics, preventative treatment, and client experience. We then computed a domain score by adding the items within each domain and dividing by the total number of items in the domain. Finally, we transformed the index into a score out of 100 by averaging the domain scores and multiplying by 100.

Principal components analysis (PCA): To create the PCA index score, we conducted an unrotated, unweighted PCA using a correlation matrix and used the factor loadings from the first principal component to create the index score. We rescaled the

For peer review only - http://bmjopen.bmj.com/site/about/guidelines.xhtml

score obtained from the first component of the PCA to a range of zero to 100 for comparability with other indices.

Analysis

We limited the readiness analysis to facilities offering ANC services and with at least one first visit ANC client observation. In order to standardize expected clinical actions, we limited the provision of care analysis to women attending the health facility for their first ANC visit. Finally, we excluded cases with incomplete data for any variables of interest.

We calculated descriptive statistics on each of the indices including mean, median, minimum, maximum, and range. The elements of the complex survey design (weights and clustering of observation) were not incorporated into the analysis as the goal of this analysis was not to make inferences about the entire population from which the sample of health facilities was drawn. In addition, for the indices created using PCA to combine items, we calculated factor loadings, the eigenvalue, and the percent of total variance explained by the first component. Since the PCA method was used to create weights for a composite variable rather than estimating a latent variable we did not report out the number of components with an eigenvalue greater than one. Next, to compare scores from the nine facility readiness indices, we categorized the continuous index scores into low, medium, and high readiness categories using tercile cut points. We then assessed the comparability of the facility classification across the indices by

For peer review only - http://bmjopen.bmj.com/site/about/guidelines.xhtml

calculating the percent agreement and Cohen's kappa, which accounts for the possibility of the agreement occurring by chance, between each combination of indices. Cohen's kappa ranges from -1 to 1 and can be interpreted as follows: < 0 no agreement, 0–0.20 slight, 0.21–0.40 fair, 0.41–0.60 moderate, 0.61–0.80 good, and 0.81–1 almost perfect agreement [39].

Next, we examined the variability and distribution of the index scores. As countries are interested in being able to compare facilities with each other to understand better and worse performers (even if they are all with the low- or high-quality band), we are interested in being able to capture that variation in an index score. The examination of variability and distribution of the index scores aims to understand the level of variability being captured but does not indicate that an index is necessarily "better" because it captures more variability. The coefficient of variation (CoV) was used to measure the variation captured by each index, with a higher CoV indicative of more variation captured in the index. Distribution of the scores was examined by assessment of floor and ceiling effects. Floor and ceiling effects were considered to be present if more than 15% of facilities achieved the lowest or highest possible index score (0% or 100%) respectively [40]. While in some settings it may be possible that greater than 15% of facilities are at 0% or 100%, the presence of floor and ceiling effects may indicate that indices have limited ability to differentiate facilities at very low and very high readiness levels. Finally, each index was assessed in terms of inclusion of items with a range of

For peer review only

variability. For each index, we tallied the number of items that were available in <30% of facilities, <40% of facilities, and >90% of facilities.

All statistical analyses were carried out using R version 3.5.1 [41]. The institutional review board at the Johns Hopkins Bloomberg School of Public Health determined this analysis to be exempt from human subjects review.

Patient and Public Involvement

It was not appropriate or possible to involve patients or the public in this work.

RESULTS

The final analytic sample consisted of 358 health facilities and 779 ANC clients in Haiti, 253 health facilities and 815 ANC clients in Malawi, and 632 health facilities and 1,681 ANC clients in Tanzania. A total of 6 ANC clients in Haiti, 44 ANC clients in Malawi, and 73 ANC clients in Tanzania were excluded from the analysis due to missing data.

Item selection

The results of the expert survey are available in (***Supplementary Table 4).*** There was some alignment between the core set of readiness items and the expert set of readiness items. The core readiness index included 21 items, the expert readiness index included 19 items, and the overlap between the two indices was 14 items. Of the 45 facility readiness items identified for inclusion in the expert survey, only four were related to human resources and of those, only one was highly rated by the expert group as essential for high quality service delivery. In addition, the expert survey respondents

For peer review only - http://bmjopen.bmj.com/site/about/guidelines.xhtml

mentioned a number of items that they felt were essential for high quality care but were missing from our survey and from SPA datasets. These items included: ensuring that clients are treated with respect and without discrimination, having the option for clients to invite their partner to participate in the consultation, discussing identification of a birth companion of choice with the client, including family members in counselling sessions, providing counselling on newborn care practices, and ensuring clients understand the counselling information they receive.

Descriptive statistics

The mean readiness score varied by index from 56.6 to 64.1 in Haiti, 52.3 to 69.6 in Malawi, and 61.2 to 75.9 in Tanzania (***Table 1, Figure 2***). Across all countries, there were no indices for which any facilities received the minimum score of zero and all countries had at least three indices for which some facilities received the maximum score of 100. In addition, in each country, the three indices that contained the same items — but used different methodologies for combining the items — generally had a similar median. For example, the core simple, core weighted, and core PCA median scores in Tanzania were 73.5, 72.1, and 73.9, respectively. Finally, in general, the indices created using PCA resulted in a larger inter-quartile range (IQR), particularly for the core and expert item indices, across all three countries.

The mean provision of care score varied by index from 38.9 to 85.9 in Haiti, 35.8 to 53.1 in Malawi, and 40.4 to 58.0 in Tanzania (***Table 1, Figure 3***). The expert PCA index

For peer review only - http://bmjopen.bmj.com/site/about/guidelines.xhtml

in Haiti was the only index in which any clients received the minimum score of zero and no countries had an index for which any clients received the maximum score of 100. The expert simple and expert weighted indices had similar median provision of care scores; however, the expert weighted index scores were higher than the simple index scores in all three countries. The expert PCA index scores had median provision of care scores which were very different from both the expert simple and expert weighted scores. For example, in Malawi the expert simple mean provision of care score was 46.9, the expert weighted mean provision of care score was 53.7, and the expert PCA mean provision of care score was 33.9.

The results from the PCA analysis are presented in ***Table 2***. Across all countries, and all indices for facility readiness, the items with the highest loadings (absolute value greater than 0.4) differed by index and country. For the core item indices, the items that loaded the highest were related to diagnostics and human resources. For the expert item indices, the items that loaded the highest were related to diagnostics. For the maximum item indices, the items that loaded the highest were related to diagnostics and basic amenities. For all facility readiness indices in all countries, the percent of variance explained by the first principal component was low, ranging from 17.21% for the core and maximum indices in Haiti to 19.49% and 20.38% for the expert set of items in Tanzania and Haiti, respectively. In addition, we found that some items such as

For peer review only - http://bmjopen.bmj.com/site/about/guidelines.xhtml

For peer review only

tetanus toxoid vaccine, de-worming medications, and syphilis testing had negative loadings.

Across all countries for provision of care, the items with the highest loadings (absolute value greater than 0.4) differed by country. For Haiti, the items that loaded the highest were related to client experience. For Malawi and Tanzania, the items that loaded the highest were related to history-taking and counselling; although for each country, the highest loadings were largely on different items. For provision of care indices in all countries, the percent of variance explained by the first principal component was low, ranging from 9.89% in Malawi to 10.49% in Haiti. In addition, we found that items such as history-taking, client education and counselling on danger signs in pregnancy, and hemoglobin testing had negative loadings.

Comparability of the Facility Classification

Table 3 presents the results of the percent agreement and Cohen's kappa coefficient among the nine facility readiness indices. Across all countries, all readiness indices had fair or better agreement. In Haiti, there were three index combinations that had fair agreement (core weighted/core PCA; core weighted/expert PCA; core weighted/maximum PCA) while the remainder of the indices had moderate or better agreement. In Malawi, there was one index combination that had fair agreement (core weighted/maximum simple) while the remainder of the indices had moderate or better

For peer review only - http://bmjopen.bmj.com/site/about/guidelines.xhtml

agreement. In Tanzania, there were two index combinations that had fair agreement (core weighted/expert PCA; core weighted/maximum PCA) while the remainder of the indices had moderate or better agreement.

Variability and Distribution of the Index

The variability and distribution of the nine facility readiness indices are presented in ***Table 4***. In Haiti, the CoV ranged from 21.12 (maximum simple readiness index) to 35.72 (expert PCA). In Malawi, the CoV ranged from 18.96 (expert PCA) to 32.09 (core PCA). In Tanzania, the CoV ranged from 19.11 (core simple) to 23.62 (expert PCA). Across all countries and all indices, there were no floor effects. Ceiling effects were limited and far below the 15% threshold. The highest percentage of ceiling effects was 3.33% which was found in Tanzania in three indices (expert simple, expert weighted, and expert PCA).

Inclusion of Items Across a Range of Frequency

Table 5 presents the percentage of facilities in which each index item was available in order to assess the inclusion of items across a range of frequency. Across all countries, the maximum item index contained the greatest number of items available in less than 40% of facilities (10 items Haiti, 12 items Malawi, and nine items in Tanzania). In Tanzania, the expert index did not include any items available in less than 40% of facilities. Within countries, the core, expert, and maximum indices had a similar number of items that were available in over 90% of facilities (four to five in Haiti, six to eight in Malawi, seven to nine in Tanzania).

For peer review only - http://bmjopen.bmj.com/site/about/guidelines.xhtml

Discussion

To our knowledge, this is the first study that uses multiple methodologies to develop and compare indices for ANC facility readiness and provision of care. Our results showed that, within a country, indices containing different items combined using different approaches were quite similar in terms of mean index score, facility classification, coefficient of variation, floor and ceiling effects, and inclusion of items with a range of variability. Although similar overall, we found that the indices created using PCA were the most different from the other indices. In addition, the core weighted index had lower agreement with the other indices when looking at facility classification. This may be the result of some domains in the core weighted index having few items, making each of those items more influential in the overall index score.

Our analysis highlighted the importance of competent, motivated human resources to all aspects of delivering high quality ANC services. Our approach to defining the core set of items highlighted the lack of metrics for human resources captured by facility surveys, as well as the inconsistency between what inputs are required for provision of care and how facility readiness is often defined. This is especially important considering there is a health workforce crisis globally which is particularly pronounced in LMICs [42-44]. Many countries are suffering from an absolute shortage of health care workers. The health workers they do have are often poorly

For peer review only - http://bmjopen.bmj.com/site/about/guidelines.xhtml

distributed within a country and many workers are deficient in skill mix and core competencies [45 46].

This study's findings also suggest that to provide more comprehensive measures of ANC quality some items could be added to SPAs and other facility surveys, particularly items related to the experience of care. While the global community is moving toward more person-centered care, data collected through health facility assessments is generally deficient in experience of care measures [47 48]. This may be due to the lack of validated instruments available for measuring the experience of maternity care [49]. In addition, measures of patient experience from health facility assessments are limited and may be subject to courtesy bias which has been found to be particularly problematic for subjective questions regarding items such as treatment by staff and consultation quality, items of interest for measuring experience of care [50]. We therefore cannot be certain if these measures are actually representative of people's experiences or if other types of questions would better capture experience of care. However, a number of recent studies have generated validated tools for measuring respectful maternity care; these may well provide a starting point for incorporating improved experience of care metrics in health facility assessments such as the SPA [51 52].

We also found that the indices differed in terms of their ease of construction and interpretation. The simple additive and weighted additive approaches were straightforward to construct because they required taking averages of items, while the

For peer review only - http://bmjopen.bmj.com/site/about/guidelines.xhtml

For peer review only

PCA necessitated more complex analysis. In addition, the simple additive approach was the easiest to interpret since facilities with more items available had a higher quality score. The weighted additive approach was also relatively easy to interpret. However, item weighting whereby each domain receives an equal weight was based on an implicit conceptual framework for quality which has not been formally validated. The PCA approach was perhaps the most difficult to interpret as the PCA score represents the linear combination of variables that explained the most variance in the data. As the loadings of each variable on the first component were used as the weights, and some items were negatively correlated and consequently had negative loadings; having all items present in a facility did not always result in the highest PCA score. In addition, the weights assigned by PCA reflected the variation in the data which was different across countries. As a result, PCA did not produce the same weights across different contexts. As we were working with a common definition for what an ANC visit should include across country contexts, this variability in loadings resulted in concerns regarding the face validity and construct validity of the PCA indices. Overall, the low percent of total variance explained by the first principle component, diverging items with high loadings between countries, negatively correlated items, and the complexity in creating and interpreting the measure highlights concerns for the use of PCA for creating quality of care indices [53]. Another study focused on comparing summary measures of quality of care for family planning found similar results in terms of the ease of construction and

For peer review only - http://bmjopen.bmj.com/site/about/guidelines.xhtml

interpretation of similar indices suggesting this finding may be relevant across service areas[54].

Due the variation in ease of construction and interpretation, different methodologies may be better suited for different purposes. For example, the simple additive approach using the maximum number of items may be easiest for Ministries of Health in LMICs to understand and implement. However, if there is particular interest in using facility readiness as a proxy for provision of care, it might be conceptually important to ensure that items align well between facility readiness and provision of care thus making the core set of items a good choice. In addition, while the weighted additive method aims to give equal weights to domains of facility readiness and provision of care, as the contents of each domain are determined by what data is available as opposed to an ideal set of items, this approach implicitly weights certain items more heavily because of the unavailability of data on other items and therefore may not be well-suited depending on the purpose of the index. While different methodologies may be better suited for different purposes, there remains a need for standardized, meaningful, valid measures of quality of care that take into account the variation in ease of construction and interpretation and can be used both by countries and at global level.

We note several limitations of the analysis. First, this analysis was conducted using data from three countries, two in Africa and one in Latin America and the

For peer review only - http://bmjopen.bmj.com/site/about/guidelines.xhtml

Caribbean, which may limit generalizability of findings to low-income countries globally. However, the findings were relatively consistent across the three countries. The two African countries are in the Southern and Eastern Africa region and represent two different types and sizes of health system within that region. Second, the SPA survey, while quite comprehensive, does not capture every aspect of quality of care that may be important to include in quality of care metrics. However, the SPA represents the most comprehensive information on quality of care in LMICs that is currently available[34]. Third, our approaches to item selection and combination were based on the most commonly used approaches in the literature. However, there are other methods that may be helpful in determining which items to include in a quality of care index, such as latent class analysis, that we did not implemented. The selection of methods for this analysis included those most likely to be useful to LMICs. Finally, this analysis does not provide a formal validation of any one ANC quality of care index. However, it does characterize various indices of ANC summary indices and provides valuable information on the similarities and differences between indices.

CONCLUSION

While the goal of this study was not to identify the best index for measuring quality of care, we did endeavor to characterize the various indices to make more information available in order to assist health systems researchers in choosing a methodology for creating quality of care indices. Overall, we found the indices to be

For peer review only - http://bmjopen.bmj.com/site/about/guidelines.xhtml

quite similar within a country. In addition, we found that different methodologies may be better suited for different purposes. Future research on the association between facility readiness and provision of care would be helpful to further characterize the quality of care indices and inform selection of an index. As quality of care becomes integral to global efforts to improve health outcomes, demand for guidance on creating standardized measures of service quality will grow. This study provides health systems researchers with a comparison of methodologies commonly used to create summary indices of ANC service quality and highlights the similarities and differences between methods. Further research will be required at global and country level to develop standardized, meaningful, valid measures of quality of care that take into account multiple services and various country contexts.

For peer review only

For peer review only - http://bmjopen.bmj.com/site/about/guidelines.xhtml

Acknowledgements

The authors wish to acknowledge the Bill & Melinda Gates Foundation for their support of this project. In addition, we would like to thank the maternal health experts who participated in the expert survey; we appreciate your time and willingness to share your expertise.

Declaration of Interests

Dr. Munos reports grants from Bill and Melinda Gates Foundation during the conduct of the study.

Role of the Funding Source

The funder had no role in the study design, data collection, analysis and interpretation of data, or manuscript writing.

Data Availability Statement

Data are available in a public, open access repository. Data is available from the DHS program after registering as a user and submitting information regarding your research purpose. The DHS program website is: https://dhsprogram.com/

For peer review only

For peer review only - http://bmjopen.bmj.com/site/about/guidelines.xhtml

References

1. Countdown to 2015: Maternal Newborn and Child Survival. Fulfilling the Health Agenda for Women and Children: The 2014 Report, 2014.
2. Souza JP, Gulmezoglu AM, Vogel J, et al. Moving beyond essential interventions for reduction of maternal mortality (the WHO Multicountry Survey on Maternal and Newborn Health): a cross-sectional study. *Lancet* 2013;381(9879):1747-55. doi: 10.1016/s0140-6736(13)60686-8 [published Online First: 2013/05/21]
3. Sobel HL, Huntington D, Temmerman M. Quality at the centre of universal health coverage. *Health Policy Plan* 2016;31(4):547-9. doi: 10.1093/heapol/czv095 [published Online First: 2015/10/01]
4. Kruk ME, Larson E, Twum-Danso NAY. Time for a quality revolution in global health. *The Lancet Global Health* 2016;4(9) doi: 10.1016/S2214-109X(16)30131-0
5. Villar J, Bergsjø P. Scientific basis for the content of routine antenatal care. I. Philosophy, recent studies, and power to eliminate or alleviate adverse maternal outcomes. *Acta obstetricia et gynecologica Scandinavica* 1997;76(1):1-14.
6. Campbell O, Graham W. Strategies for reducing maternal mortality: getting on with what works. *The Lancet* 2006;368(9543):1284-99. doi: 10.1016/S0140-6736(06)69381-1
7. Lassi ZS, Salam RA, Das JK, et al. Essential pre-pregnancy and pregnancy interventions for improved maternal, newborn and child health. *Reproductive Health* 2014;11(Suppl 1):S2-S2. doi: 10.1186/1742-4755-11-S1-S2
8. Callaghan-Koru JA, McMahon SA, Chebet JJ, et al. A qualitative exploration of health workers' and clients' perceptions of barriers to completing four antenatal care visits in Morogoro Region, Tanzania. *Health policy and planning* 2016;31(8):1039-49. doi: 10.1093/heapol/czw034

For peer review only

For peer review only - http://bmjopen.bmj.com/site/about/guidelines.xhtml

9. Gross K, Schellenberg Armstrong J, Kessy F, et al. Antenatal care in practice: an exploratory study in antenatal care clinics in the Kilombero Valley, south-eastern Tanzania. *BMC Pregnancy and Childbirth* 2011;11:36:1-11.

10. Gupta S, Yamada G, Mpembeni R, et al. Factors associated with four or more antenatal care visits and its decline among pregnant women in Tanzania between 1999 and 2010. *PLoS ONE* 2014;9(7) doi: 10.1371/journal.pone.0101893

11. Donabedian A. The Quality of Care: How Can It Be Assessed? *JAMA* 1988;260(12):1743-43. doi: 10.1001/jama.1988.03410120089033

12. Donabedian A. Evaluating the quality of medical care. *The Milbank quarterly* 2005;83(4):691-729. doi: 10.1111/j.1468-0009.2005.00397.x

13. Donabedian A. The Definition of Quality and Approaches to Its Assessment Vol 1. Explorations in Quality Assessment and Monitoring. Ann Arbor, Michigan: Health Administration Press 1980.

14. Institute of Medicine Committee on Quality of Health Care in America. Crossing the quality chasm : a new health system for the 21st century. Washington, DC: National Academy Press 2001.

15. World Health Organization. Quality of care: a process for making strategic choices in health systems. Geneva, Switzerland, 2006.

16. Bollen KA, Bauldry S. Three Cs in Measurement Models: Causal Indicators, Composite Indicators, and Covariates. *Psychol Methods* 2011;16(3):265-84. doi: 10.1037/a0024448

17. Diamond-Smith N, Sudhinaraset M, Montagu D. Clinical and perceived quality of care for maternal, neonatal and antenatal care in Kenya and Namibia: the service provision assessment. *Reproductive Health* 2016;13(1):92-92. doi: 10.1186/s12978-016-0208-y

For peer review only - http://bmjopen.bmj.com/site/about/guidelines.xhtml

18. Leslie HH, Fink G, Nsona H, et al. Obstetric Facility Quality and Newborn Mortality in Malawi: A Cross-Sectional Study. *PLoS medicine* 2016;13(10):e1002151. doi: 10.1371/journal.pmed.1002151
19. Kruk ME, Leslie HH, Verguet S, et al. Quality of basic maternal care functions in health facilities of five African countries: an analysis of national health system surveys. *The Lancet Global Health* 2016;4(11):e845-e55. doi: 10.1016/S2214-109X(16)30180-2
20. Lee E, Madhavan S, Bauhoff S. Levels and variations in the quality of facility-based antenatal care in Kenya: evidence from the 2010 service provision assessment. *Health Policy and Planning* 2016;31(6):777-84. doi: 10.1093/heapol/czv132
21. Sharma J, Leslie HH, Kundu F, et al. Poor Quality for Poor Women? Inequities in the Quality of Antenatal and Delivery Care in Kenya. *PloS one* 2017;12(1):e0171236. doi: 10.1371/journal.pone.0171236
22. Kanyangarara M, Chou VB. Linking household surveys and health facility assessments to estimate intervention coverage for the Lives Saved Tool (LiST). *BMC public health* 2017;17(Suppl 4):780. doi: 10.1186/s12889-017-4743-4
23. Nguhiu PK, Barasa EW, Chuma J. Determining the effective coverage of maternal and child health services in Kenya, using demographic and health survey data sets: tracking progress towards universal health coverage. *Tropical Medicine & International Health* 2017;22(4):442-53. doi: 10.1111/tmi.12841
24. O'Neill K, Takane M, Sheffel A, et al. Monitoring service delivery for universal health coverage: the Service Availability and Readiness Assessment. *Bulletin of the World Health Organization* 2013;91(12):923-31. doi: 10.2471/BLT.12.116798
25. Sipsma HL, Curry LA, Kakoma J-B, et al. Identifying characteristics associated with performing recommended practices in maternal and newborn care among health facilities in Rwanda: a cross-sectional study. *Human Resources for Health* 2012;10(1):13-13. doi: 10.1186/1478-4491-10-13

For peer review only

26. Van Huy N, Nam YS, Van Thanh N, et al. WHO's Service Availability and Readiness Assessment of primary health care services of commune health centers in a rural district of Northern Vietnam. *International Journal of Health Planning and Management* 2017 doi: 10.1002/hpm.2429

27. Sheffel A, Karp C, Creanga AA. Use of Service Provision Assessments and Service Availability and Readiness Assessments for monitoring quality of maternal and newborn health services in low-income and middle-income countries. *BMJ Global Health* 2018;3(6) doi: http://dx.doi.org/10.1136/bmjgh-2018-001011

28. Do M, Wang W, Hembling J, et al. Quality of antenatal care and client satisfaction in Kenya and Namibia. *International Journal for Quality in Health Care* 2017;29(2):183-93. doi: 10.1093/intqhc/mzx001

29. Leslie HH, Gage A, Nsona H, et al. Training And Supervision Did Not Meaningfully Improve Quality Of Care For Pregnant Women Or Sick Children In Sub-Saharan Africa. *Health Aff (Millwood)* 2016;35(9):1716-24. doi: 10.1377/hlthaff.2016.0261 [published Online First: 2016/09/09]

30. Leslie HH, Malata A, Ndiaye Y, et al. Effective coverage of primary care services in eight high-mortality countries. *BMJ Glob Health* 2017;2(3):e000424. doi: 10.1136/bmjgh-2017-000424 [published Online First: 2018/04/11]

31. Wang W, Winner M, Burgert-Brucker CR. Limited Service Availability, Readiness, and Use of Facility-Based Delivery Care in Haiti: A Study Linking Health Facility Data and Population Data. *Global Health: Science and Practice* 2017;5(2):244-60. doi: 10.9745/GHSP-D-16-00311

32. Hutchinson PL, Do M, Agha S. Measuring client satisfaction and the quality of family planning services: A comparative analysis of public and private health facilities in Tanzania, Kenya and Ghana. *BMC Health Services Research* 2011;11(1):203-03. doi: 10.1186/1472-6963-11-203

For peer review only

33. Ngo DKL, Sherry TB, Bauhoff S. Health system changes under pay-for-performance: the effects of Rwanda's national programme on facility inputs. *Health Policy and Planning* 2017;32(1):11-20. doi: 10.1093/heapol/czw091

34. ICF. The DHS Program: SPA Overview 2017 [Available from: http://dhsprogram.com/What-We-Do/Survey-Types/SPA.cfm.

35. Institut Haïtien de l'Enfance, ICF International. Évaluation de Prestation des Services de Soins de Santé, Haïti, 2013, 2014.

36. Malawi Ministry of Health, ICF International. Malawi Service Provision Assessment (MSPA) 2013-2014: MoH and ICF International, 2014.

37. Tanzania Ministry of Health & Social Welfare, Tanzania National Bureau of Statistics, ICF International. Tanzania Service Provision Assessment Survey 2014-2015, 2016.

38. World Health Organization. Standards for improving quality of maternal and newborn care in health facilities. Geneva, Switzerland, 2016.

39. Landis J, Koch G. The measurement of observer agreement for categorical data. *Biometrics* 1977;33(1):159-74.

40. Terwee CB, Bot SD, de Boer MR, et al. Quality criteria were proposed for measurement properties of health status questionnaires. *J Clin Epidemiol* 2007;60(1):34-42. doi: 10.1016/j.jclinepi.2006.03.012 [published Online First: 2006/12/13]

41. R: A Language and Environment for Statistical Computing [program]. Vienna, Austria: R Foundation for Statistical Computing, 2017.

42. Chen L, Evans T, Anand S, et al. Human resources for health: overcoming the crisis. *The Lancet* 2004;364(9449):1984-90.

43. Fulton BD, Scheffler RM, Sparkes SP, et al. Health workforce skill mix and task shifting in low income countries: a review of recent evidence. *Hum Resour Health* 2011;9:1. doi: 10.1186/1478-4491-9-1 [published Online First: 2011/01/13]

For peer review only

For peer review only - http://bmjopen.bmj.com/site/about/guidelines.xhtml

44. Campbell J, Dussault G, Buchan J, et al. A Universal Truth: No Health Without a Workforce. Recife, Brazil and Geneva,Switzerland: Global Health Workforce Alliance and World Health Organization, 2014.

45. Joint Learning Initiative. Human resources for health: overcoming the crisis. Cambridge, MA: Global Equity Initiative, Harvard University, 2004.

46. Ranson MK, Chopra M, Atkins S, et al. Priorities for research into human resources for health in low- and middle-income countries. *Bull World Health Organ* 2010;88(6):435-43. doi: 10.2471/blt.09.066290 [published Online First: 2010/06/12]

47. Sheffel A, Heidkamp R, Mpembeni R, et al. Understanding client and provider perspectives of antenatal care service quality: a qualitative multi-method study from Tanzania. *The Journal of Global Health* 2018; [submitted, under review]

48. Brizuela V, Leslie HH, Sharma J, et al. Measuring quality of care for all women and newborns: how do we know if we are doing it right? A review of facility assessment tools. *Lancet Glob Health* 2019;7(5):e624-e32. doi: 10.1016/s2214-109x(19)30033-6 [published Online First: 2019/03/23]

49. Bohren MA, Vogel JP, Hunter EC, et al. The Mistreatment of Women during Childbirth in Health Facilities Globally: A Mixed-Methods Systematic Review. *PLoS Med* 2015;12(6) doi: 10.1371/journal.pmed.1001847 [published Online First: 2015/07/01]

50. Glick P. How reliable are surveys of client satisfaction with healthcare services? Evidence from matched facility and household data in Madagascar. *Soc Sci Med* 2009;68(2):368-79. doi: 10.1016/j.socscimed.2008.09.053 [published Online First: 2008/11/26]

51. Vedam S, Stoll K, Martin K, et al. The Mother's Autonomy in Decision Making (MADM) scale: Patient-led development and psychometric testing of a new instrument to evaluate experience of maternity care. *PLoS One*

For peer review only - http://bmjopen.bmj.com/site/about/guidelines.xhtml

2017;12(2):e0171804. doi: 10.1371/journal.pone.0171804 [published Online First: 2017/02/24]

52. Vedam S, Stoll K, Rubashkin N, et al. The Mothers on Respect (MOR) index: measuring quality, safety, and human rights in childbirth. *SSM - Population Health* 2017;3:201-10. doi: https://doi.org/10.1016/j.ssmph.2017.01.005

53. Sharker MY, Nasser M, Abedin J, et al. The risk of misclassifying subjects within principal component based asset index. *Emerging themes in epidemiology* 2014;11:6. doi: 10.1186/1742-7622-11-6 [published Online First: 2014/07/06]

54. Mallick L, Temsah G, Wang W. Comparing summary measures of quality of care for family planning in Haiti, Malawi, and Tanzania. *PLoS One* 2019;14(6):e0217547. doi: 10.1371/journal.pone.0217547 [published Online First: 2019/06/08]

For peer review only

For peer review only - http://bmjopen.bmj.com/site/about/guidelines.xhtml

TABLES

Table 1: Descriptive Statistics of Facility Readiness and Provision of Care Indices

	Facility Readiness Indices									Provision of Care Indices		
	core simple	core weighted	core PCA	expert simple	expert weighted	expert PCA	maximum simple	maximum weighted	maximum PCA	simple	weighted	PCA
**Haiti (N = 358 facilities)**										**Haiti (N = 779 clients)**		
mean	63.8	64.0	59.4	64.1	61.3	58.2	57.8	56.6	59.8	38.9	45.0	85.9
SD	14.0	15.1	21.4	16.1	16.6	21.6	12.6	14.0	15.9	8.0	10.4	15.9
median	63.3	65.5	59.4	64.3	61.3	57.5	57.9	57.4	60.6	38.8	45.4	91.7
min	23.8	16.4	17.6	17.1	18.1	12.9	27.6	20.1	23.4	12.2	14.0	0.0
max	92.9	93.8	99.8	100.0	100.0	100.0	87.5	87.7	95.2	79.6	76.3	96.0
IQR	19.8	20.7	40.9	24.6	24.2	39.5	17.8	20.2	24.6	8.5	12.9	7.1
**Malawi (N = 253 facilities)**										**Malawi (N = 815 clients)**		
mean	62.9	65.2	52.3	68.7	67.6	69.6	61.3	61.0	63.1	47.5	53.1	35.8
SD	13.2	13.3	16.7	13.2	13.1	13.2	11.5	11.4	12.9	9.9	10.2	14.5
median	61.9	66.4	46.4	68.4	67.8	66.3	59.9	60.0	59.8	46.9	53.7	33.9
min	33.3	30.0	24.5	38.2	34.3	38.9	35.5	32.7	37.0	22.4	22.9	10.7
max	100.0	100.0	100.0	100.0	100.0	100.0	92.8	94.3	100.0	72.0	75.5	70.8
IQR	19.0	16.7	20.5	17.5	16.9	16.5	16.7	14.1	18.7	14.3	13.5	24.5
**Tanzania (N = 632 facilities)**										**Tanzania (N = 1,681 clients)**		
mean	71.4	71.0	70.2	75.9	71.8	74.7	61.2	62.8	64.1	50.8	58.0	40.4
SD	13.6	14.6	15.5	15.4	14.6	17.8	13.0	13.6	14.6	10.9	11.2	12.3
median	73.5	72.1	73.9	78.9	73.2	78.4	61.8	63.9	64.9	50.5	58.6	39.5
min	23.8	18.6	20.4	28.9	28.6	20.3	27.6	25.5	24.7	16.3	18.5	9.9
max	100.0	100.0	100.0	100.0	100.0	100.0	94.7	92.1	100.0	98.0	99.3	98.2
IQR	21.4	20.3	25.8	23.8	20.2	30.2	20.2	19.9	23.0	14.8	15.1	17.0

For peer review only - http://bmjopen.bmj.com/site/about/guidelines.xhtml

Table 2: Results from the Facility Readiness Indices and Provision of Care Indices Principal Component Analyses

Facility Readiness Indices									
**Item name**	**Haiti**			**Malawi**			**Tanzania**		
	**core set**	**expert set**	**maximum set**	**core set**	**expert set**	**maximum set**	**core set**	**expert set**	**maximum set**
Equipment and supplies									
Blood pressure apparatus	0.05	0.04	0.04	0.37	0.34	0.37	0.34	0.33	0.28
Examination light	0.08		0.14	0.44		0.49	0.34		0.38
Stethoscope	0.14	0.11	0.17	0.16	0.13	0.18	0.28	0.23	0.23
Adult weighing scale	0.22		0.19	0.25		0.32	0.43		0.39
Tape measure for fundal height	0.13		0.18	0.22		0.28	0.21		0.19
Examination bed	0.23	0.22	0.26	0.17	0.21	0.18	0.14	0.10	0.15
Latex gloves	0.24	0.52	0.49	0.14	0.32	0.39	0.08	0.50	0.51
Single use syringes		0.25	0.26		0.24	0.21		0.14	0.14
Soap and running water OR Alcohol based hand rub		0.41	0.41		0.38	0.40		0.52	0.57
Disinfectant		0.40	0.43		0.34	0.30		0.32	0.30
Appropriate storage of sharps waste (sharps box)			-0.12			0.09			0.11
Appropriate storage of infectious waste (pedal bin with lid)			0.01			0.03			0.17
Safe final disposal of sharps (incineration)			0.20			0.26			0.06
Safe final disposal of infectious wastes (incineration)			0.20			0.25			0.06
Medical masks			0.06			0.16			0.35
Gowns			0.31			0.21			0.38
Eye protection			0.29			0.28			0.23
Diagnostics									
Hemoglobin	0.63	0.61	0.60	0.75	0.75	0.70	0.72	0.71	0.69
Urine dipstick- protein	0.78	0.77	0.69	0.85	0.84	0.77	0.77	0.78	0.68
Urine dipstick- glucose	0.74	0.73	0.66	0.80	0.78	0.71	0.78	0.79	0.69
Grouping and Rhesus factor	0.18		0.19	0.53		0.47	0.29		0.37
Syphilis RDT / RPR	0.78	0.77	-0.04	0.68	0.68	-0.05	0.65	0.65	-0.04
HIV testing / RDT	0.78	0.79	0.72	0.18	0.23	0.21	0.41	0.41	0.35
Medicines									
Iron tablets	0.10	0.11	0.18	0.11	0.17	0.15	0.26	0.26	0.25
Folic acid tablets	0.15	0.16	0.19	0.16	0.24	0.22	0.20	0.21	0.21
TT vaccine	-0.05	-0.02	0.01	0.20	0.25	0.24	0.33	0.30	0.27
De-worming drugs	0.09		0.12	-0.12		-0.06	0.15		0.09
Basic Amenities									
Power		0.18	0.55		0.17	0.29		0.44	0.41
Improved water source		-0.08	0.23		0.10	0.14		0.03	0.39
Room with auditory and visual privacy	-0.10	0.46	-0.07	0.06	0.20	0.06	0.08	0.26	0.06
Sanitation facilities			0.42			0.10			0.25
Communication equipment			0.25			0.27			0.60
Computer with email/internet			0.62			0.71			0.66

For peer review only - http://bmjopen.bmj.com/site/about/guidelines.xhtml

Emergency transportation			0.38			0.01			0.33
Human resources									
Guidelines ANC	-0.06		0.00	0.15		0.20	0.38	0.33	0.36
Proportion of staff trained in ANC in last two years	0.43	0.39	0.39	0.47	0.46	0.44	0.40		0.34
Proportion of staff receiving supervision in the last six months	0.60		0.59	0.43		0.40	0.42		0.37
Proportion of staff reporting opportunities for promotion			0.17			-0.05			-0.07
**Eigenvalue**	3.61	3.87	3.61	3.74	3.42	3.74	3.66	3.70	3.66
**Percent of total variance**	17.21	20.38	17.21	17.80	17.98	17.80	17.44	19.49	17.44

Provision of Care Indices

**Item name**	**Haiti**	**Malawi**	**Tanzania**
History and counselling			
History-taking: Personal history: client age	0.03	0.24	0.28
History-taking: Personal history: medications client is taking	-0.02	0.05	0.22
History-taking: Personal history: date last menstrual period began	0.02	0.25	0.32
History-taking: Personal history: any prior pregnancy	-0.09	0.44	0.31
History-taking: Past medical history for prior pregnancies: still birth	-0.18	0.69	0.46
History-taking: Past medical history for prior pregnancies: infant died in the first week of life	-0.01	0.59	0.45
History-taking: Past medical history for prior pregnancies: heavy bleeding during or after delivery	0.03	0.73	0.50
History-taking: Past medical history for prior pregnancies: previous assisted delivery	-0.02	0.73	0.42
History-taking: Past medical history for prior pregnancies: previous spontaneous abortion	0.00	0.69	0.39
History-taking: Past medical history for prior pregnancies: previous multiple pregnancies	0.02	0.54	0.37
History-taking: Past medical history for prior pregnancies: previous prolonged labor	0.00	0.44	0.39
History-taking: Past medical history for prior pregnancies: pregnancy-induced hypertension	-0.13	0.73	0.35
History-taking: Past medical history for prior pregnancies: pregnancy related convulsions	0.00	0.68	0.37
History-taking: Past medical history for prior pregnancies: high fever or	-0.04	0.21	0.35

For peer review only

infection during prior pregnancy/pregnancies			
History-taking: History of complaints in current pregnancy: vaginal bleeding	0.01	0.31	0.58
History-taking: History of complaints in current pregnancy: fever	0.07	0.15	0.51
History-taking: History of complaints in current pregnancy: headache or blurred vision	0.03	0.18	0.56
History-taking: History of complaints in current pregnancy: swollen face or hands or extremities	0.03	0.25	0.48
History-taking: History of complaints in current pregnancy: tiredness or breathlessness	-0.02	0.17	0.51
History-taking: History of complaints in current pregnancy: fetal movement (loss of, excessive, normal)	0.04	0.10	0.47
History-taking: History of complaints in current pregnancy: cough or difficulty breathing for 3 weeks or longer	-0.04	0.21	0.36
Client education and counselling: Process of pregnancy and its complications	0.01	0.16	0.27
Client education and counselling: Diet and nutrition	0.03	0.20	0.28
Client education and counselling: Danger signs in pregnancy	-0.01	0.14	0.65
Client education and counselling: Voluntary counselling and testing for HIV	0.08	0.12	0.27
Client education and counselling: Breast feeding	-0.02	0.07	0.29
Client education and counselling: Plans of delivery (emergency preparedness, place of delivery, transportation, financial arrangements)	0.07	0.28	0.37
Examination			
Examination and observation: Edema (other than ankle-specify)	0.08	0.21	0.24
Examination and observation: Blood pressure	0.03	0.09	0.10
Examination and observation: Weight	0.06	0.11	0.16
Obstetric complications: Palpate the client's abdomen for fundal height	0.15	0.11	0.19
Diagnostics			
Laboratory investigations: Hemoglobin	0.03	-0.01	0.12
Laboratory investigations: Grouping and rhesus factor	0.04	0.15	0.16
Laboratory investigations: RPR (syphilis test)	0.03	0.04	0.16

For peer review only - http://bmjopen.bmj.com/site/about/guidelines.xhtml

For peer review only

Laboratory investigations: HIV testing	0.04	0.04	0.23
Laboratory investigations: Urine-protein, sugar, acetone	0.03	0.03	0.09
Preventative treatment			
Drug administration and immunization: Iron and or folic acid provided or prescribed	0.06	0.13	0.18
Drug administration and immunization: Tetanus toxoid provided or prescribed	0.06	0.03	0.09
Client experience			
No problem: discuss problems or concerns	0.65	-0.01	0.14
No problem: explanation you received	0.64	0.08	0.15
No problem: how the staff treated you	0.73	0.05	0.09
No problem: privacy from having others see	0.72	0.07	0.20
No problem: privacy from having others hear	0.74	0.10	0.20
No problem: time you waited	0.44	0.04	0.01
No problem: number of days services are available	0.73	0.01	0.11
No problem: hours of service	0.75	0.00	0.06
No problem: cost for services or treatments	0.65	0.10	0.08
No problem: availability of medicines	0.62	0.02	0.06
No problem: cleanliness of the facility	0.69	0.03	0.06
**Eigenvalue**	5.14	4.84	5.08
**Percent of total variance**	10.49	9.89	10.37

For peer review only

For peer review only - http://bmjopen.bmj.com/site/about/guidelines.xhtml

Table 3: Percent Agreement and Cohen's Kappa Coefficient Among Nine Facility Readiness Indices

Haiti

	Core simple		Core weighted		Core PCA		Expert simple		Expert weighted		Expert PCA		Maximum simple		Maximum weighted	
	% Agreement	Kappa	% Agreement	Kappa	% Agreement	Kappa	% Agreement	Kappa	% Agreement	Kappa	% Agreement	Kappa	% Agreement	Kappa	% Agreement	Kappa
Core simple																
Core weighted	67.91	0.52														
Core PCA	69.96	0.55	51.48	0.27												
Expert simple	79.85	0.70	65.60	0.48	74.45	0.62										
Expert weighted	74.97	0.62	65.60	0.48	66.37	0.50	77.41	0.66								
Expert PCA	71.89	0.58	53.53	0.30	86.01	0.79	81.77	0.73	67.01	0.51						
Maximum simple	77.02	0.66	62.64	0.44	63.54	0.45	75.87	0.64	68.68	0.53	68.29	0.52				
Maximum weighted	82.54	0.74	65.98	0.49	67.91	0.52	77.79	0.67	73.68	0.61	69.58	0.54	84.47	0.77		
Maximum PCA	68.68	0.53	51.73	0.28	79.46	0.69	75.48	0.63	65.08	0.48	81.90	0.73	76.77	0.65	74.58	0.62

Malawi

	Core simple		Core weighted		Core PCA		Expert simple		Expert weighted		Expert PCA		Maximum simple		Maximum weighted	
	% Agreement	Kappa	% Agreement	Kappa	% Agreement	Kappa	% Agreement	Kappa	% Agreement	Kappa	% Agreement	Kappa	% Agreement	Kappa	% Agreement	Kappa
Core simple																
Core weighted	75.58	0.63														
Core PCA	82.09	0.73	65.40	0.48												
Expert simple	78.04	0.67	65.28	0.48	71.90	0.58										
Expert weighted	71.66	0.57	66.01	0.49	66.38	0.50	74.48	0.62								
Expert PCA	76.69	0.65	62.70	0.44	79.51	0.69	81.10	0.72	70.18	0.55						
Maximum simple	73.13	0.60	59.88	0.40	74.85	0.62	70.67	0.56	63.68	0.45	70.18	0.55				

For peer review only - http://bmjopen.bmj.com/site/about/guidelines.xhtml

Maximum weighted	77.42	0.66	64.91	0.47	77.55	0.66	73.50	0.60	65.03	0.48	70.31	0.55	79.51	0.69		
Maximum PCA	79.02	0.69	63.19	0.45	86.63	0.80	78.40	0.68	66.75	0.50	80.86	0.71	82.58	0.74	80.61	0.71

Tanzania

	Core simple		Core weighted		Core PCA		Expert simple		Expert weighted		Expert PCA		Maximum simple		Maximum weighted	
	% Agreement	Kappa	% Agreement	Kappa	% Agreement	Kappa	% Agreement	Kappa	% Agreement	Kappa	% Agreement	Kappa	% Agreement	Kappa	% Agreement	Kappa
Core simple																
Core weighted	75.19	0.63														
Core PCA	88.28	0.82	66.57	0.50												
Expert simple	72.69	0.59	63.36	0.45	76.80	0.65										
Expert weighted	70.55	0.56	63.36	0.45	73.88	0.61	80.31	0.70								
Expert PCA	73.65	0.60	59.73	0.40	81.56	0.72	89.77	0.85	76.26	0.64						
Maximum simple	71.62	0.57	63.36	0.45	72.75	0.59	79.83	0.70	71.62	0.57	77.57	0.66				
Maximum weighted	77.33	0.66	65.91	0.49	77.51	0.66	75.01	0.63	71.92	0.58	74.30	0.61	84.47	0.77		
Maximum PCA	71.45	0.57	59.55	0.39	76.74	0.65	80.55	0.71	72.93	0.59	81.86	0.73	84.06	0.76	79.00	0.69

Legend	<0	0 — 0.20	0.21 — 0.40	0.41 — 0.60	0.61 — 0.80	0.81 — 1.0
	No agreement/ Worse than chance	Slight	Fair	Moderate	Good	Near perfect

For peer review only - http://bmjopen.bmj.com/site/about/guidelines.xhtml

Table 4: Variability and Distribution of Index Scores for the Nine Facility Readiness Indices

	Haiti			Malawi			Tanzania		
	CoV	Floor Effects	Ceiling Effects	CoV	Floor Effects	Ceiling Effects	CoV	Floor Effects	Ceiling Effects
Core simple	21.20	0.00	0.00	21.70	0.00	1.10	19.11	0.00	0.48
Core weighted	22.67	0.00	0.00	21.45	0.00	1.10	20.83	0.00	0.48
Core PCA	34.61	0.00	0.00	32.09	0.00	1.10	21.96	0.00	0.48
Expert simple	24.30	0.00	0.39	19.64	0.00	1.23	20.14	0.00	3.33
Expert weighted	26.01	0.00	0.39	19.82	0.00	1.23	20.24	0.00	3.33
Expert PCA	35.72	0.00	0.51	18.96	0.00	1.23	23.62	0.00	3.33
Maximum simple	21.12	0.00	0.00	19.33	0.00	0.00	21.31	0.00	0.00
Maximum weighted	24.07	0.00	0.00	18.98	0.00	0.00	21.69	0.00	0.00
Maximum PCA	26.02	0.00	0.00	20.67	0.00	1.10	22.73	0.00	0.36

For peer review only

For peer review only - http://bmjopen.bmj.com/site/about/guidelines.xhtml

Table 5: Inclusion of Items in an Index Across a Range of Frequencies for the Facility Readiness Indices

	Haiti			Malawi			Tanzania		
	No./% items <30% frequency	No./% items <40% frequency	No./% items >90% frequency	No./% items <30% frequency	No./% items <40% frequency	No./% items >90% frequency	No./% items <30% frequency	No./% items <40% frequency	No./% items >90% frequency
Core (21 items)	2 (10%)	4 (19%)	5 (24%)	4 (19%)	7 (33%)	6 (29%)	2 (10%)	2 (10%)	8 (38%)
Expert (19 items)	2 (11%)	3 (16%)	4 (21%)	2 (11%)	5 (26%)	7 (37%)	0 (0%)	0 (0%)	7 (37%)
Maximum (38 items)	8 (21%)	10 (26%)	5 (13%)	8 (21%)	12 (32%)	8 (21%)	5 (13%)	9 (24%)	9 (24%)

For peer review only

For peer review only - http://bmjopen.bmj.com/site/about/guidelines.xhtml

FIGURE TITLES AND LEGENDS

Figure 1: Process for Index Creation

- no legend

Figure 2: Distribution of Facility Readiness Scores

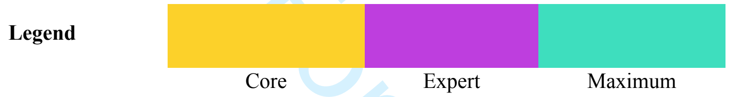

Figure 3: Distribution of Provision of Care Scores

- no legend

For peer review only

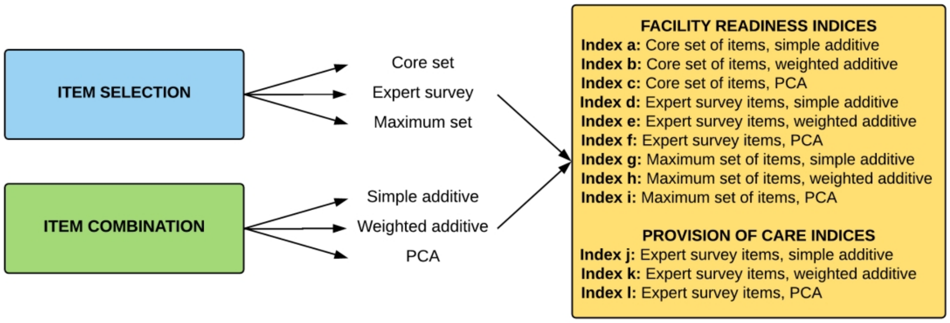
ITEM SELECTION
Core set
Expert survey
Maximum set
ITEM COMBINATION
Simple additive
Weighted additive
PCA
FACILITY READINESS INDICES
Index a: Core set of items, simple additive
Index b: Core set of items, weighted additive
Index c: Core set of items, PCA
Index d: Expert survey items, simple additive
Index e: Expert survey items, weighted additive
Index f: Expert survey items, PCA
Index g: Maximum set of items, simple additive
Index h: Maximum set of items, weighted additive
Index i: Maximum set of items, PCA
PROVISION OF CARE INDICES
Index j: Expert survey items, simple additive
Index k: Expert survey items, weighted additive
Index l: Expert survey items, PCA

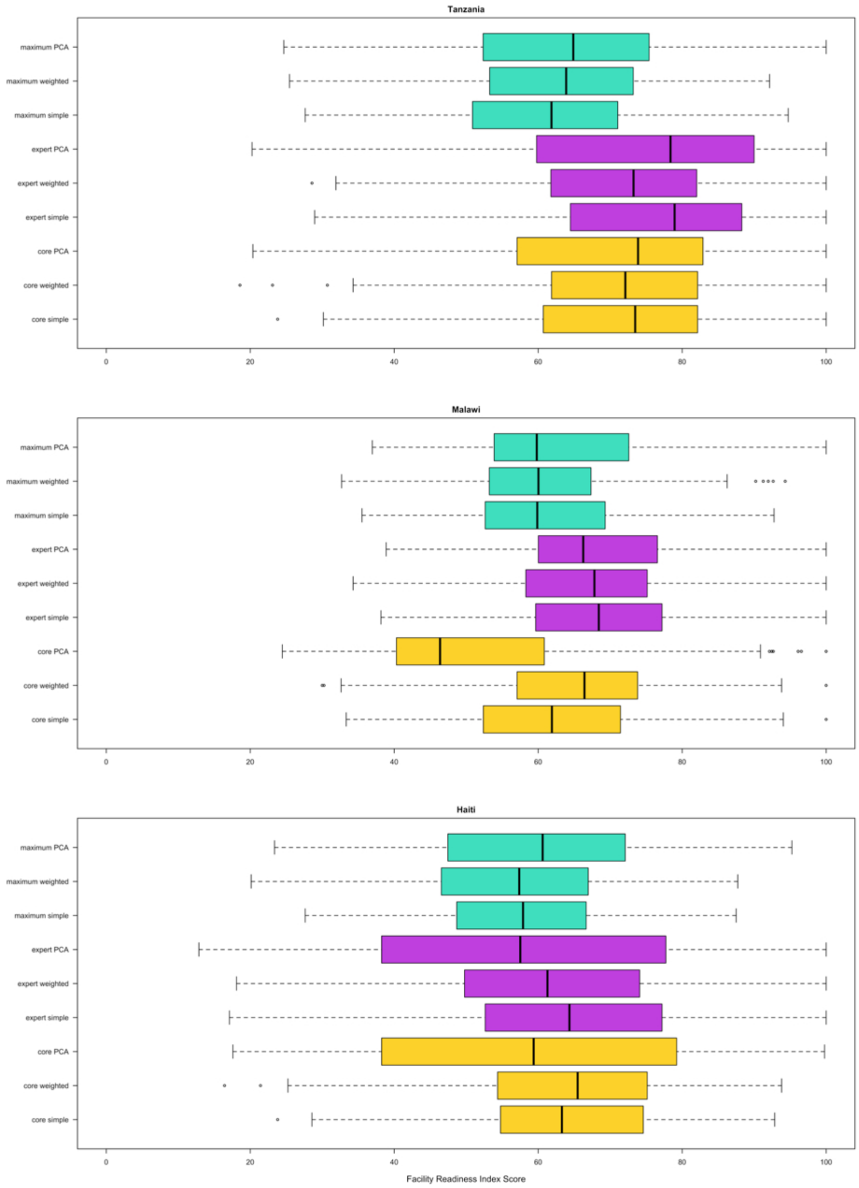
Tanzania
Malawi
Haiti
maximum PCA
maximum weighted
maximum simple
expert PCA
expert weighted
expert simple
core PCA
core weighted
core simple
0
20
40
60
80
100
Facility Readiness Index Score

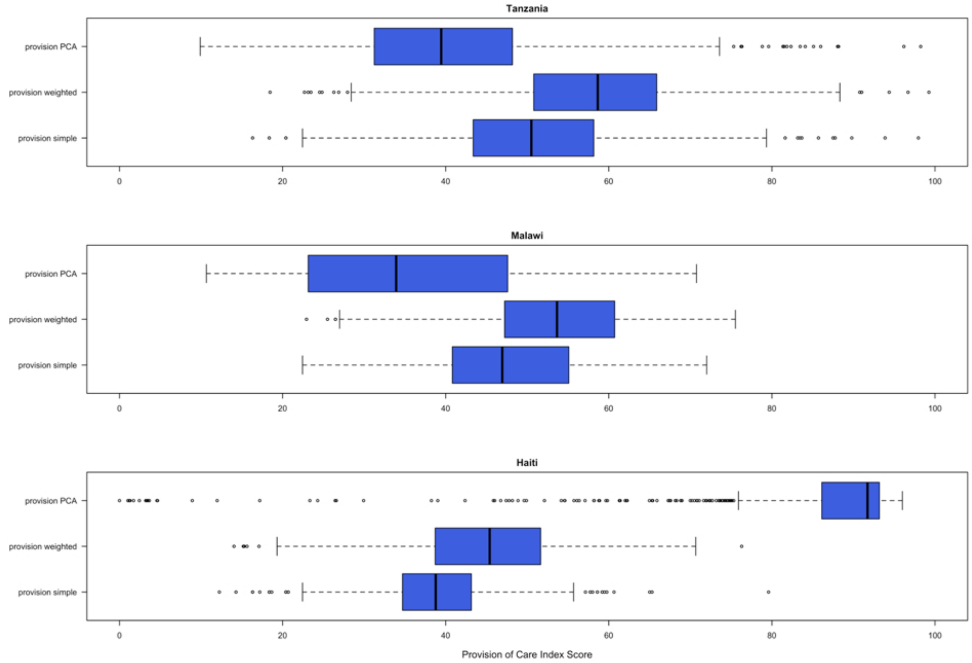
Tanzania
provision PCA
provision weighted
provision simple
0
20
40
60
80
100
Malawi
provision PCA
provision weighted
provision simple
0
20
40
60
80
100
Haiti
provision PCA
provision weighted
provision simple
0
20
40
60
80
100
Provision of Care Index Score

Supplementary Tables

Supplementary Table 1: Summary of Literature Using SPA Data to Create ANC Quality of Care Indices

Author	Year	Country	Type of index and number of items	Item selection	Item combination
Diamond-Smith et al	2016	Kenya, Namibia	Facility readiness: 8 items	DHS tracer indicators of ANC quality	Simple additive
Do et al	2017	Kenya, Namibia	Facility readiness: 14 items<br>Provision of care: 7 items	Donabedian framework and the WHO SARA indicators	Factor analysis
Kruk et al	2017	Kenya, Malawi, Namibia, Rwanda, Senegal, Uganda, Tanzania	Provision of care: 8 items	Clinical guidelines from the WHO	Simple additive
Lee et al	2016	Kenya	Facility readiness and provision of care: 14 items	WHO definition of quality of care	Weighted additive
Sharma et al	2017	Kenya	Facility readiness: 15 items<br>Provision of care: 40 items	Donabedian framework, IOM definition of quality of care, and Focused ANC Model Checklist	Simple additive
Sipsma et al	2012	Rwanda	Provision of care: 8 items	Recommended practices derived from clinical guidelines	Simple additive

For peer review only

Supplementary Table 2: List of Items Included in the Facility Readiness Indices, by Domain

#	Item Name	Core set	Expert survey	Maximum set
	EQUIPMENT AND SUPPLIES *Availability of...*			
1	Blood pressure apparatus	X	X	X
2	Examination light	X		X
3	Stethoscope	X	X	X
4	Adult weighing scale	X		X
5	Tape measure for fundal height	X		X
6	Examination bed	X	X	X
7	Latex gloves		X	X
8	Single use syringes	X	X	X
9	Soap and water OR alcohol-based hand rub		X	X
10	Disinfectant		X	X
11	Appropriate storage of sharps waste (sharps box)			X
12	Appropriate storage of infectious waste (pedal bin with lid and liner)			X
13	Safe final disposal of sharps (incineration)			X
14	Safe final disposal of infectious wastes (incineration)			X
15	Medical masks			X
16	Gowns			X
17	Eye protection			X
	DIAGNOSTICS *Availability on-site to test for...*			
18	Hemoglobin	X	X	X
19	Urine dipstick- protein	X	X	X
20	Urine dipstick- glucose	X	X	X
21	Grouping and Rhesus factor	X		X
22	Syphilis RDT / RPR	X	X	X
23	HIV testing / RDT	X	X	X
	MEDICINES AND COMMODITIES *Availability on-site of...*			
24	Iron tablets	X	X	X

For peer review only - http://bmjopen.bmj.com/site/about/guidelines.xhtml

#	Item Name	Core set	Expert survey	Maximum set
25	Folic acid tablets	X	X	X
26	Tetanus toxoid vaccine	X	X	X
27	De-worming drugs (mebendazole / albendazole)	X		X
	BASIC AMENITIES<br>*Availability of…*			
28	Power			X
29	Improved water source		X	X
30	Room with auditory and visual privacy	X	X	X
31	Sanitation facilities		X	X
32	Communication equipment			X
33	Computer with email/internet access			X
34	Emergency transportation			X
	HUMAN RESOURCES<br>*Availability of…*			
35	Guidelines for ANC available at the facility	X		X
36	Proportion of health facility staff providing ANC services trained in ANC in the last two years	X	X	X
37	Proportion of health facility staff providing ANC services who received supervision in the last six months	X		X
38	Proportion of health facility staff providing ANC services who report availability of opportunities for promotion in their current job			X

For peer review only - http://bmjopen.bmj.com/site/about/guidelines.xhtml

Supplementary Table 3: List of Items Included in the Provision of Care Indices, by Domain

#	Item Name	Corresponding Readiness Domain
	HISTORY-TAKING AND CLIENT EDUCATION/COUNSELLING *During ANC visit the provider asks about/counsels the client on…*	
1	Personal history: client age	Human Resources
2	Personal history: medications client is taking	Human Resources
3	Personal history: date last menstrual period began	Human Resources
4	Personal history: any prior pregnancy	Human Resources
5	Past medical history for prior pregnancies: still birth	Human Resources
6	Past medical history for prior pregnancies: infant died in the first week of life	Human Resources
7	Past medical history for prior pregnancies: heavy bleeding during or after delivery	Human Resources
8	Past medical history for prior pregnancies: previous assisted delivery	Human Resources
9	Past medical history for prior pregnancies: previous spontaneous abortion	Human Resources
10	Past medical history for prior pregnancies: multiple pregnancies	Human Resources
11	Past medical history for prior pregnancies: prolonged labor	Human Resources
12	Past medical history for prior pregnancies: pregnancy-induced hypertension	Human Resources
13	Past medical history for prior pregnancies: pregnancy related convulsions	Human Resources
14	Past medical history for prior pregnancies: high fever or infection during prior pregnancy	Human Resources
15	History of complaints in current pregnancy: vaginal bleeding	Human Resources
16	History of complaints in current pregnancy: fever	Human Resources
17	History of complaints in current pregnancy: headache or blurred vision	Human Resources
18	History of complaints in current pregnancy: swollen face or hands or extremities	Human Resources
19	History of complaints in current pregnancy: tiredness or breathlessness	Human Resources
20	History of complaints in current pregnancy: fetal movement (loss of, excessive, normal)	Human Resources
21	History of complaints in current pregnancy: cough or difficulty breathing for three weeks or longer	Human Resources
22	Client education/counselling: Process of pregnancy and its complications	Human Resources
23	Client education/counselling: Diet and nutrition	Human Resources
24	Client education/counselling: Danger signs in pregnancy	Human Resources

For peer review only - http://bmjopen.bmj.com/site/about/guidelines.xhtml

#	Item Name	Corresponding Readiness Domain
25	Client education/counselling: Voluntary counselling and testing for HIV	Human Resources
26	Client education/counselling: Breastfeeding	Human Resources
27	Client education/counselling: Plans of delivery (emergency preparedness, place of delivery, transportation, financial arrangements)	Human Resources
	EXAMINATION *During ANC visit the provider examines the client for…*	
28	Oedema	Human Resources
29	Blood pressure	Human Resources, Equipment
30	Weight	Human Resources, Equipment
31	Palpating the client's abdomen for fundal height	Human Resources, Equipment
	DIAGNOSTICS *During ANC visit the provider performs or refers the client to test for…*	
32	Hemoglobin	Human Resources, Diagnostics
33	Grouping and rhesus factor	Human Resources, Diagnostics
34	Syphilis / RPR	Human Resources, Diagnostics
35	HIV	Human Resources, Diagnostics
36	Urine – protein, glucose	Human Resources, Diagnostics
	PREVENTATIVE TREATMENT *During ANC visit the provider administers or prescribes…*	
37	Iron and/or folic acid	Human Resources, Medicines
38	Tetanus toxoid	Human Resources, Medicines
	CLIENT EXPERIENCE *During ANC visit…*	
39	Client is able to discuss problems or concerns with provider	Human Resources
40	Client satisfied with the amount of explanation received about the problem or treatment	Human Resources

For peer review only - http://bmjopen.bmj.com/site/about/guidelines.xhtml

#	Item Name	Corresponding Readiness Domain
41	Client satisfied with how the staff treated them	Human Resources
42	Privacy from having others see the consultation	Human Resources, Basic Amenities
43	Privacy from having others hear the consultation	Human Resources, Basic Amenities
44	Client satisfied with the wait time	Human Resources
45	Client satisfied with the number of days services are available at the facility	Human Resources
46	Client satisfied with the hours of service at the facility	Human Resources
47	Client satisfied with the cost for services or treatments	Human Resources
48	Client satisfied with the availability of medicines at the facility	Human Resources, Medicines
49	Client satisfied with the cleanliness of the facility	Human Resources

For peer review only

Supplementary Table 4: Expert Survey Results (mean, sd, min, max, n)

Item Name	Mean	Standard Deviation	Min.	Max.	N
EQUIPMENT AND SUPPLIES *Availability of...*					
Blood pressure apparatus	3.933	0.258	3	4	15
Examination light	2.600	0.828	1	4	15
Fetal stethoscope	3.733	0.594	2	4	15
Stethoscope	3.467	0.834	2	4	15
Adult weighing scale	3.000	0.845	2	4	15
Thermometer	3.533	0.834	1	4	15
Tape measure for fundal height	3.333	0.724	2	4	15
Height board	2.692	0.947	1	4	13
Speculum	3.429	0.646	2	4	14
Examination bed	3.429	0.938	1	4	14
Latex gloves	3.600	0.632	2	4	15
Single use syringes	3.643	0.745	2	4	14
Soap and water OR alcohol-based hand rub	3.733	0.704	2	4	15
Disinfectant	3.533	0.640	2	4	15
Appropriate storage of sharps waste (sharps box)	3.400	0.910	1	4	15
Appropriate storage of infectious waste (pedal bin with lid and liner)	3.333	0.976	1	4	15
Safe final disposal of sharps (incineration)	3.200	0.941	1	4	15
Safe final disposal of infectious wastes (incineration)	3.133	0.990	1	4	15
Medical masks	2.200	0.775	1	4	15
Gowns	2.000	0.845	1	4	15
Eye protection	2.067	0.884	1	4	15
Gum boots	1.667	0.724	1	3	15
DIAGNOSTICS *Availability on-site to test for...*					
Hemoglobin	3.800	0.414	3	4	15
Urine dipstick- protein	3.733	0.458	3	4	15

For peer review only - http://bmjopen.bmj.com/site/about/guidelines.xhtml

Item Name	Mean	Standard Deviation	Min.	Max.	N
Urine dipstick- glucose	3.467	0.743	2	4	15
Grouping and Rhesus factor	3.357	0.745	2	4	14
Syphilis RDT / RPR	3.667	0.488	3	4	15
HIV testing / RDT	3.667	0.488	3	4	15
MEDICINES AND COMMODITIES *Availability on-site of…*					
Iron tablets	3.800	0.414	3	4	15
Folic acid tablets	3.667	0.617	2	4	15
Tetanus toxoid vaccine	3.800	0.414	3	4	15
Intermittent Preventative Treatment (IPT) drug	3.667	0.488	3	4	15
Insecticide treated nets (ITNs) or vouchers	3.267	0.799	2	4	15
De-worming drugs (mebendazole / albendazole)	3.000	0.784	2	4	14
BASIC AMENITIES *Availability of…*					
Power	3.400	0.737	2	4	15
Improved water source	3.667	0.617	2	4	15
Room with auditory and visual privacy	3.533	0.640	2	4	15
Sanitation facilities	3.600	0.507	3	4	15
Communication equipment	3.267	0.884	1	4	15
Computer with email/internet access	2.267	1.033	1	4	15
Emergency transportation	3.400	0.986	1	4	15
HUMAN RESOURCES *Availability of…*					
Guidelines for ANC available at the facility	3.267	0.884	1	4	15
Proportion of health facility staff providing ANC services trained in ANC in the last two years	3.400	0.737	2	4	15
Proportion of health facility staff providing ANC services who received supervision in the last six months	3.267	0.799	2	4	15

For peer review only - http://bmjopen.bmj.com/site/about/guidelines.xhtml

Item Name	Mean	Standard Deviation	Min.	Max.	N
Proportion of health facility staff providing ANC services who report availability of opportunities for promotion in their current job	2.500	0.760	2	4	14
HISTORY-TAKING *During ANC visit the provider asks about…*					
Personal history: client age	3.533	0.640	2	4	15
Personal history: medications client is taking	3.733	0.458	3	4	15
Personal history: date last menstrual period began	3.933	0.258	3	4	15
Personal history: any prior pregnancy	3.867	0.352	3	4	15
Family history	3.200	0.862	2	4	15
Social history	3.067	0.704	2	4	15
Past medical history for prior pregnancies: still birth	3.867	0.352	3	4	15
Past medical history for prior pregnancies: infant died in the first week of life	3.600	0.632	2	4	15
Past medical history for prior pregnancies: heavy bleeding during or after delivery	3.733	0.458	3	4	15
Past medical history for prior pregnancies: previous assisted delivery	3.733	0.458	3	4	15
Past medical history for prior pregnancies: previous spontaneous abortion	3.467	0.834	2	4	15
Past medical history for prior pregnancies: multiple pregnancies	3.600	0.737	2	4	15
Past medical history for prior pregnancies: prolonged labor	3.600	0.632	2	4	15
Past medical history for prior pregnancies: pregnancy-induced hypertension	4.000	0.000	4	4	15
Past medical history for prior pregnancies: pregnancy related convulsions	4.000	0.000	4	4	15

For peer review only - http://bmjopen.bmj.com/site/about/guidelines.xhtml

Item Name	Mean	Standard Deviation	Min.	Max.	N
Past medical history for prior pregnancies: high fever or infection during prior pregnancy	3.533	0.640	2	4	15
History of complaints in current pregnancy: vaginal bleeding	4.000	0.000	4	4	15
History of complaints in current pregnancy: fever	3.867	0.352	3	4	15
History of complaints in current pregnancy: headache or blurred vision	4.000	0.000	4	4	15
History of complaints in current pregnancy: swollen face or hands or extremities	4.000	0.000	4	4	15
History of complaints in current pregnancy: tiredness or breathlessness	3.667	0.816	1	4	15
History of complaints in current pregnancy: fetal movement (loss of, excessive, normal)	3.933	0.258	3	4	15
History of complaints in current pregnancy: cough or difficulty breathing for three weeks or longer	3.733	0.458	3	4	15
History of complaints in current pregnancy: amniotic leakage	3.786	0.579	2	4	14
EXAMINATION *During ANC visit the provider examines the client for…*					
Head to toe (whole body)	2.600	1.121	1	4	15
Pallor	3.400	0.632	2	4	15
Oedema	3.733	0.458	3	4	15
Breast	2.533	0.990	1	4	15
Lungs and heart	2.600	1.056	1	4	15
OBSERVATION AND CLINICAL INVESTIGATION *During ANC visit the provider assesses the client's…*					
Blood pressure	4.000	0.000	4	4	15
Weight	3.133	0.834	2	4	15
Temperature	3.333	0.900	1	4	15
Pulse	3.357	0.929	1	4	14

For peer review only - http://bmjopen.bmj.com/site/about/guidelines.xhtml

Item Name	Mean	Standard Deviation	Min.	Max.	N
OBSTETRIC COMPLICATIONS<br>*During ANC visit the provider assesses the client for potential obstetric complications by…*					
Palpating the client's abdomen for fundal height	3.667	0.617	2	4	15
Palpating the client's abdomen for fetal presentation	3.600	0.632	2	4	15
Listening to the client's abdomen for fetal heartbeat	3.800	0.561	2	4	15
Conducting an ultrasound/ referring client for ultrasound/ look at recent ultrasound report	2.600	0.986	1	4	15
PELVIC EXAMINATION<br>*During ANC visit the provider conducts a pelvic exam with…*					
Soft tissue assessment/ Vaginal examination	2.154	1.214	1	4	13
Bony pelvic assessment	2.571	1.342	1	4	14
LABORATORY INVESTIGATIONS<br>*During ANC visit the provider performs or refers the client to test for…*					
Hemoglobin	3.733	0.458	3	4	15
Grouping and rhesus factor	3.357	0.633	2	4	14
Syphilis / RPR	3.667	0.488	3	4	15
HIV	3.867	0.352	3	4	15
Urine – protein	3.667	0.617	2	4	15
Urine – glucose	3.400	0.828	2	4	15
Urine – acetone	2.500	1.087	1	4	12
DRUG ADMINISTRATION AND IMMUNIZATION<br>*During ANC visit the provider administers or prescribes…*					
Iron and/or folic acid	3.800	0.414	3	4	15
Antimalarials	3.429	0.756	2	4	14
Tetanus toxoid	3.600	0.737	2	4	15
CLIENT EDUCATION AND COUNSELLING<br>*During ANC visit the provider counsels the client on…*					
Process of pregnancy and its complications	3.733	0.594	2	4	15
Diet and nutrition	3.600	0.632	2	4	15

For peer review only - http://bmjopen.bmj.com/site/about/guidelines.xhtml

Item Name	Mean	Standard Deviation	Min.	Max.	N
Rest and exercise in pregnancy	2.933	0.704	2	4	15
Personal hygiene	2.867	0.990	1	4	15
Danger signs in pregnancy	3.933	0.258	3	4	15
Use of drugs in pregnancy	3.400	0.828	2	4	15
Effects of STI/HIV/AIDS	3.467	0.834	2	4	15
Voluntary counselling and testing for HIV	3.667	0.617	2	4	15
Care of breasts	2.867	0.743	2	4	15
Breastfeeding	3.867	0.352	3	4	15
Symptoms/signs of labor	3.867	0.516	2	4	15
Plans of delivery (emergency preparedness, place of delivery, transportation, financial arrangements)	3.733	0.458	3	4	15
Plans for postpartum care	3.400	0.632	2	4	15
Family planning	3.533	0.640	2	4	15
Harmful habits (e.g. smoking, drug abuse, alcoholism)	3.533	0.640	2	4	15
Schedule of return visit	3.867	0.352	3	4	15
CLIENT EXPERIENCE *During ANC visit…*					
Client is able to discuss problems or concerns with provider	3.867	0.352	3	4	15
Client satisfied with the amount of explanation received about the problem or treatment	3.600	0.632	2	4	15
Client satisfied with how the staff treated them	3.667	0.617	2	4	15
Privacy from having others see the consultation	3.533	0.640	2	4	15
Privacy from having others hear the consultation	3.533	0.640	2	4	15
Client satisfied with the wait time	3.133	0.834	2	4	15
Client satisfied with the number of days services are available at the facility	3.000	0.756	2	4	15

For peer review only - http://bmjopen.bmj.com/site/about/guidelines.xhtml

Item Name	Mean	Standard Deviation	Min.	Max.	N
Client satisfied with the hours of service at the facility	3.067	0.704	2	4	15
Client satisfied with the cost for services or treatments	3.333	0.617	2	4	15
Client satisfied with the availability of medicines at the facility	3.467	0.516	3	4	15
Client satisfied with the cleanliness of the facility	3.533	0.516	3	4	15

For peer review only

Expert Survey: Antenatal Care Service Quality

INTRODUCTION:
We invite you to participate in a brief online survey as part of a study aiming to assess the relationship between structural quality and process quality for antenatal care (ANC) services in low- and middle-income countries being conducted at the Johns Hopkins Bloomberg School of Public Health. You have been identified as an expert in maternal health with experience working in low- and middle-income countries and as such we would greatly appreciate your contribution to this work.

In order to explore the relationship between structural quality and process quality for ANC services, we first need to develop ANC quality of care metrics. While there are many definitions and frameworks for defining quality of care, there is a lack of consensus on indicators for measuring the quality of antenatal care. The goal of this survey is to elicit expert opinion on the items that are important to include in measures of ANC service quality. We expect that the results of this work will be published in a dissertation, and eventually a journal article.

The survey can be accessed at the following link:
https://tinyurl.com/ExpertSurvey-ANC

The survey should take approximately 20-30 minutes to complete. We would greatly appreciate if you can respond to the survey by 10 September 2018. If you have any questions, please feel free to contact Ashley Sheffel (asheffel@jhu.edu) or Melinda Munos (mmunos@jhu.edu).

Many thanks in advance for your time and contribution.

CONSENT:
By checking "Yes" I agree to participate in the survey. I understand the purpose and nature of this activity and I am participating voluntarily. I understand that I can stop taking the survey at any time, without any penalty or consequences. I understand that any personal information collected through this survey will remain confidential.

YES □ → continue NO □ → end survey

BACKGROUND:
In order to create a comprehensive list of items related to ANC service quality, a review of the Service Availability and Readiness Assessment Indicators and the WHO Focused Antenatal Care Guidelines was conducted along with a review of the data available in the Service Provision Assessment questionnaire, which is the main data source for this research. The identified items have been organized according to the dimensions of quality of care as proposed by the WHO Quality of Care Framework for Maternal and Newborn Health: essential physical resources, competent and motivated human resources, provision of care, and experience of care. Brief descriptions of each dimension are provided below.

DIMENSION DESRIPTIONS:
Physical resources: This dimension includes items related to the availability of an appropriate physical environment, equipment, supplies, medicines, and diagnostics required to provide ANC services.

For peer review only - http://bmjopen.bmj.com/site/about/guidelines.xhtml

Human resources: This dimension includes items related to the availability of competent, motivated human resources to provide ANC services.

Provision of care: This dimension includes items related to use of evidence-based practices, information systems in which record- keeping allows review, and functioning systems for referral between different levels of care.

Experience of care: This dimension includes items related to effective communication with women and their families about the care provided, their expectations and their rights; care with respect and preservation of dignity; and access to the social and emotional support of their choice.

INSTRUCTIONS: For each dimension, a set of items has been identified that are related to delivery of ANC. The goal is to identify the items which are most important for delivery of a high-quality ANC service ***within each dimension***. For each item, please provide a rating of importance of the item to ANC service quality based on your expert opinion. Circle only one response option per item. If you are unfamiliar with an item or don't know how to rate the item, please select "Don't know".

RATINGS:
1 = Unimportant/Non-informative for measuring ANC service quality
2= Somewhat important for measuring ANC service quality
3 = Very important for measuring ANC service quality
4 = Essential for measuring ANC service quality
98 = Don't know

For peer review only

For peer review only - http://bmjopen.bmj.com/site/about/guidelines.xhtml

#	Item Name	Response options				
		Unimportant / Non-informative	Somewhat important	Very important	Essential	Don't Know
ESSENTIAL PHYSICAL RESOURCES						
EQUIPMENT AND SUPPLIES<br>*Availability of…*						
1	Blood pressure apparatus	1	2	3	4	98
2	Examination light	1	2	3	4	98
3	Fetal stethoscope	1	2	3	4	98
4	Stethoscope	1	2	3	4	98
5	Adult weighing scale	1	2	3	4	98
6	Thermometer	1	2	3	4	98
7	Tape measure for fundal height	1	2	3	4	98
8	Height board	1	2	3	4	98
9	Speculum	1	2	3	4	98
10	Examination bed	1	2	3	4	98
11	Latex gloves	1	2	3	4	98
12	Single use syringes	1	2	3	4	98
13	Soap and water OR alcohol based hand rub	1	2	3	4	98
14	Disinfectant	1	2	3	4	98
15	Appropriate storage of sharps waste (sharps box)	1	2	3	4	98
16	Appropriate storage of infectious waste (pedal bin with lid and liner)	1	2	3	4	98
17	Safe final disposal of sharps (incineration)	1	2	3	4	98
18	Safe final disposal of infectious wastes (incineration)	1	2	3	4	98
19	Medical masks	1	2	3	4	98
20	Gowns	1	2	3	4	98
21	Eye protection	1	2	3	4	98
22	Gum boots	1	2	3	4	98
DIAGNOSTICS<br>*Availability on-site to test for…*						
23	Hemoglobin	1	2	3	4	98
24	Urine dipstick- protein	1	2	3	4	98
25	Urine dipstick- glucose	1	2	3	4	98
26	Grouping and Rhesus factor	1	2	3	4	98
27	Syphilis RDT / RPR	1	2	3	4	98

For peer review only - http://bmjopen.bmj.com/site/about/guidelines.xhtml

#	Item Name	Response options				
		Unimportant / Non-informative	Somewhat important	Very important	Essential	Don't Know
28	HIV testing / RDT	1	2	3	4	98
MEDICINES AND COMMODITIES<br>*Availability on-site of…*						
29	Iron tablets	1	2	3	4	98
30	Folic acid tablets	1	2	3	4	98
31	Tetanus toxoid vaccine	1	2	3	4	98
32	Intermittent Preventative Treatment (IPT) drug	1	2	3	4	98
33	Insecticide treated nets (ITNs) or vouchers	1	2	3	4	98
34	De-worming drugs (mebendazole / albendazole)	1	2	3	4	98
BASIC AMENITIES<br>*Availability of…*						
35	Power	1	2	3	4	98
36	Improved water source	1	2	3	4	98
37	Room with auditory and visual privacy	1	2	3	4	98
38	Sanitation facilities	1	2	3	4	98
39	Communication equipment	1	2	3	4	98
40	Computer with email/internet access	1	2	3	4	98
41	Emergency transportation	1	2	3	4	98
COMPETENT, MOTIVATED HUMAN RESOURCES						
42	Guidelines for ANC available at the facility	1	2	3	4	98
43	At least one health facility staff member trained in ANC in the last two years	1	2	3	4	98
44	At least 75% of staff offering ANC at the facility have received supervision in the last six months	1	2	3	4	98
45	At least 75% of staff offering ANC at the facility report availability of opportunities for promotion in their current job	1	2	3	4	98
PROVISION OF CARE						
HISTORY TAKING<br>*During ANC visit the provider asks about…*						
46	Personal history: client age	1	2	3	4	98
47	Personal history: medications client is taking	1	2	3	4	98
48	Personal history: date last menstrual period began	1	2	3	4	98
49	Personal history: any prior pregnancy	1	2	3	4	98
50	Family history	1	2	3	4	98

For peer review only

For peer review only - http://bmjopen.bmj.com/site/about/guidelines.xhtml

#	Item Name	Response options				
		Unimportant / Non-informative	Somewhat important	Very important	Essential	Don't Know
51	Social history	1	2	3	4	98
52	Past medical history for prior pregnancies: still birth	1	2	3	4	98
53	Past medical history for prior pregnancies: infant died in the first week of life	1	2	3	4	98
54	Past medical history for prior pregnancies: heavy bleeding during or after delivery	1	2	3	4	98
55	Past medical history for prior pregnancies: previous assisted delivery	1	2	3	4	98
56	Past medical history for prior pregnancies: previous spontaneous abortion	1	2	3	4	98
57	Past medical history for prior pregnancies: multiple pregnancies	1	2	3	4	98
58	Past medical history for prior pregnancies: prolonged labor	1	2	3	4	98
59	Past medical history for prior pregnancies: pregnancy-induced hypertension	1	2	3	4	98
60	Past medical history for prior pregnancies: pregnancy related convulsions	1	2	3	4	98
61	Past medical history for prior pregnancies: high fever or infection during prior pregnancy	1	2	3	4	98
62	History of complaints in current pregnancy: vaginal bleeding	1	2	3	4	98
63	History of complaints in current pregnancy: fever	1	2	3	4	98
64	History of complaints in current pregnancy: headache or blurred vision	1	2	3	4	98
65	History of complaints in current pregnancy: swollen face or hands or extremities	1	2	3	4	98
66	History of complaints in current pregnancy: tiredness or breathlessness	1	2	3	4	98
67	History of complaints in current pregnancy: fetal movement (loss of, excessive, normal)	1	2	3	4	98
68	History of complaints in current pregnancy: cough or difficulty breathing for three weeks or longer	1	2	3	4	98
69	History of complaints in current pregnancy: amniotic leakage	1	2	3	4	98
EXAMINATION During ANC visit the provider examines the client for…						
70	Head to toe (whole body)	1	2	3	4	98
71	Pallor	1	2	3	4	98
72	Oedema	1	2	3	4	98
73	Breast	1	2	3	4	98
74	Lungs and heart	1	2	3	4	98
OBSERVATION AND CLINICAL INVESTIGATION During ANC visit the provider assesses the client's…						

For peer review only - http://bmjopen.bmj.com/site/about/guidelines.xhtml

#	Item Name	Response options				
		Unimportant / Non-informative	Somewhat important	Very important	Essential	Don't Know
75	Blood pressure	1	2	3	4	98
76	Weight	1	2	3	4	98
77	Temperature	1	2	3	4	98
78	Pulse	1	2	3	4	98
OBSTETRIC COMPLICATIONS<br>*During ANC visit the provider assesses the client for potential obstetric complications by…*						
79	Palpating the client's abdomen for fundal height	1	2	3	4	98
80	Palpating the client's abdomen for fetal presentation	1	2	3	4	98
81	Listening to the client's abdomen for fetal heartbeat	1	2	3	4	98
82	Conducting an ultrasound/ referring client for ultrasound/ look at recent ultrasound report	1	2	3	4	98
PELVIC EXAMINATION<br>*During ANC visit the provider conducts a pelvic exam with…*						
83	Soft tissue assessment/ Vaginal examination	1	2	3	4	98
84	Bony pelvic assessment	1	2	3	4	98
LABORATORY INVESTIGATIONS<br>*During ANC visit the provider performs or refers the client to test for…*						
85	Hemoglobin	1	2	3	4	98
86	Grouping and rhesus factor	1	2	3	4	98
87	Syphilis / RPR	1	2	3	4	98
88	HIV	1	2	3	4	98
89	Urine – protein	1	2	3	4	98
90	Urine -sugar	1	2	3	4	98
91	Urine- acetone	1	2	3	4	98
DRUG ADMINISTRATION AND IMMUNIZATION<br>*During ANC visit the provider administers or prescribes…*						
92	Iron and/or folic acid	1	2	3	4	98
93	Antimalarials	1	2	3	4	98
94	Tetanus toxoid	1	2	3	4	98
CLIENT EDUCATION AND COUNSELING<br>*During ANC visit the provider counsels the client on…*						
95	Process of pregnancy and its complications	1	2	3	4	98
96	Diet and nutrition	1	2	3	4	98
97	Rest and exercise in pregnancy	1	2	3	4	98
98	Personal hygiene	1	2	3	4	98

For peer review only

For peer review only - http://bmjopen.bmj.com/site/about/guidelines.xhtml

#	Item Name	Response options				
		Unimportant / Non-informative	Somewhat important	Very important	Essential	Don't Know
99	Danger signs in pregnancy	1	2	3	4	98
100	Use of drugs in pregnancy	1	2	3	4	98
101	Effects of STI/HIV/AIDS	1	2	3	4	98
102	Voluntary counselling and testing for HIV	1	2	3	4	98
103	Care of breasts	1	2	3	4	98
104	Breastfeeding	1	2	3	4	98
105	Symptoms/signs of labor	1	2	3	4	98
106	Plans of delivery (emergency preparedness, place of delivery, transportation, financial arrangements)	1	2	3	4	98
107	Plans for postpartum care	1	2	3	4	98
108	Family planning	1	2	3	4	98
109	Harmful habits (e.g. smoking, drug abuse, alcoholism)	1	2	3	4	98
110	Schedule of return visit	1	2	3	4	98
EXPERIENCE OF CARE						
111	Client is able to discuss problems or concerns with provider	1	2	3	4	98
112	Client satisfied with the amount of explanation received about the problem or treatment	1	2	3	4	98
113	Client satisfied with how the staff treated them	1	2	3	4	98
114	Privacy from having others see the consultation	1	2	3	4	98
115	Privacy from having others hear the consultation	1	2	3	4	98
116	Client satisfied with the wait time	1	2	3	4	98
117	Client satisfied with the number of days services are available at the facility	1	2	3	4	98
118	Client satisfied with the hours of service at the facility	1	2	3	4	98
119	Client satisfied with the cost for services or treatments	1	2	3	4	98
120	Client satisfied with the availability of medicines at the facility	1	2	3	4	98
121	Client satisfied with the cleanliness of the facility	1	2	3	4	98

Are there any items you feel are essential for delivering a high-quality ANC service that do not appear in this questionnaire?

____

____

For peer review only - http://bmjopen.bmj.com/site/about/guidelines.xhtml

____
____
____

RESPONDENT INFORMATION:

Please answer the following question about yourself and your current role. This information will only be used for internal purposes and will not be disseminated.

1. Name: ___________________________
2. Email address: ___________________________
3. Organization: ___________________________
4. Position title: ___________________________
5. Years of experience in maternal health: ___________________________

The survey is now complete. Thank you for helping us with our research. We greatly appreciate your time and insights.

For peer review only

For peer review only - http://bmjopen.bmj.com/site/about/guidelines.xhtml
